# YBX1 as an adaptive RNA hub in cancer: linking state-dependent RNA regulation to tumor immunity, metabolic reprogramming, and therapy resistance

**DOI:** 10.3389/fimmu.2026.1926363

**Published:** 2026-08-21

**Authors:** Zhang XiaoTian, Wang YiTong, Feng Chongchong

**Affiliations:** Department of Clinical Laboratory, The Second Hospital of Jilin University, Changchun, Jilin, China

**Keywords:** adaptive RNA hub, cancer metabolism, therapy resistance, tumor immunity, YBX1

## Abstract

Y-box binding protein 1 (YBX1) has been implicated across an unusually broad range of malignancies and processes: immune remodeling, epithelial plasticity, metabolic rewiring, epitranscriptomic reading, and resistance to chemotherapy, targeted agents, and checkpoint blockade. A linear one-gene/one-pathway oncogene model does not readily accommodate this breadth. We argue that the apparent diffuseness reflects a context-dependent regulatory node rather than experimental noise, and develop the hypothesis that YBX1 acts as an *adaptive RNA/transcriptional hub*, a regulator whose transcript outputs are set by cellular state rather than by a fixed binding program, which acts in both the transcriptional and post-transcriptional compartments, and which sits inside feedback loops linking downstream metabolic states back to its own activity. We organize the literature into three coupled layers: a state code, in which post-translational modifications, ubiquitin balance, localization, and phase separation determine which YBX1 is active (Layer 1); an RNA program, in which m5C reading and non-coding-RNA scaffolds are associated with a restricted survival transcriptome (Layer 2); and the immune, metabolic, and plasticity phenotypes these outputs generate (Layer 3). Evidence further suggests that tumor-specific dependency is carried by the configuration of the YBX1/YBX2/YBX3 family rather than by any single member. We specify what would falsify the framework: if state-resolved readouts do not predict downstream circuit activity better than total YBX1 abundance, the hub reduces to a promiscuous, abundant RNA-binding protein whose correlations are epiphenomenal. We give explicit weight to evidence resisting an oncogenic reading: circuits in which restraining YBX1 is tumor-suppressive, non-coding-RNA and family-level interactions running in opposite directions, and effectors regulated divergently between tumors, treating these as boundary conditions rather than exceptions. This reframing shifts the actionable question from whether YBX1 is high to which YBX1-dependent circuit a tumor uses; its value remains contingent on prospective, state- and circuit-level validation.

## 1 Introduction

Cancer progression reflects a continuously evolving state of transcriptional and metabolic plasticity, shaped at every turn by microenvironmental stressors and the selective pressure imposed by therapy itself, and driving the ongoing adaptation of malignant cells (1–5). Genomic alterations establish much of the oncogenic landscape (6, 7), yet it has become increasingly clear that post-transcriptional regulatory programs, governed by microenvironmental signaling and RNA-level control, are also important determinants of transcript stability, translation efficiency, and degradation dynamics within cancer cells (8–11). RNA-binding proteins (RBPs) have, as a consequence, emerged as central players in cancer cell plasticity, given their capacity to coordinate RNA fate without requiring any permanent change to the genome (12, 13). Rather than serving as passive chaperones of RNA, many RBPs instead integrate intracellular signaling with selective transcript regulation (14–16), a property that likely accounts for their ability to support rapid adaptation to environmental and therapeutic stress.

**Figure 1:**
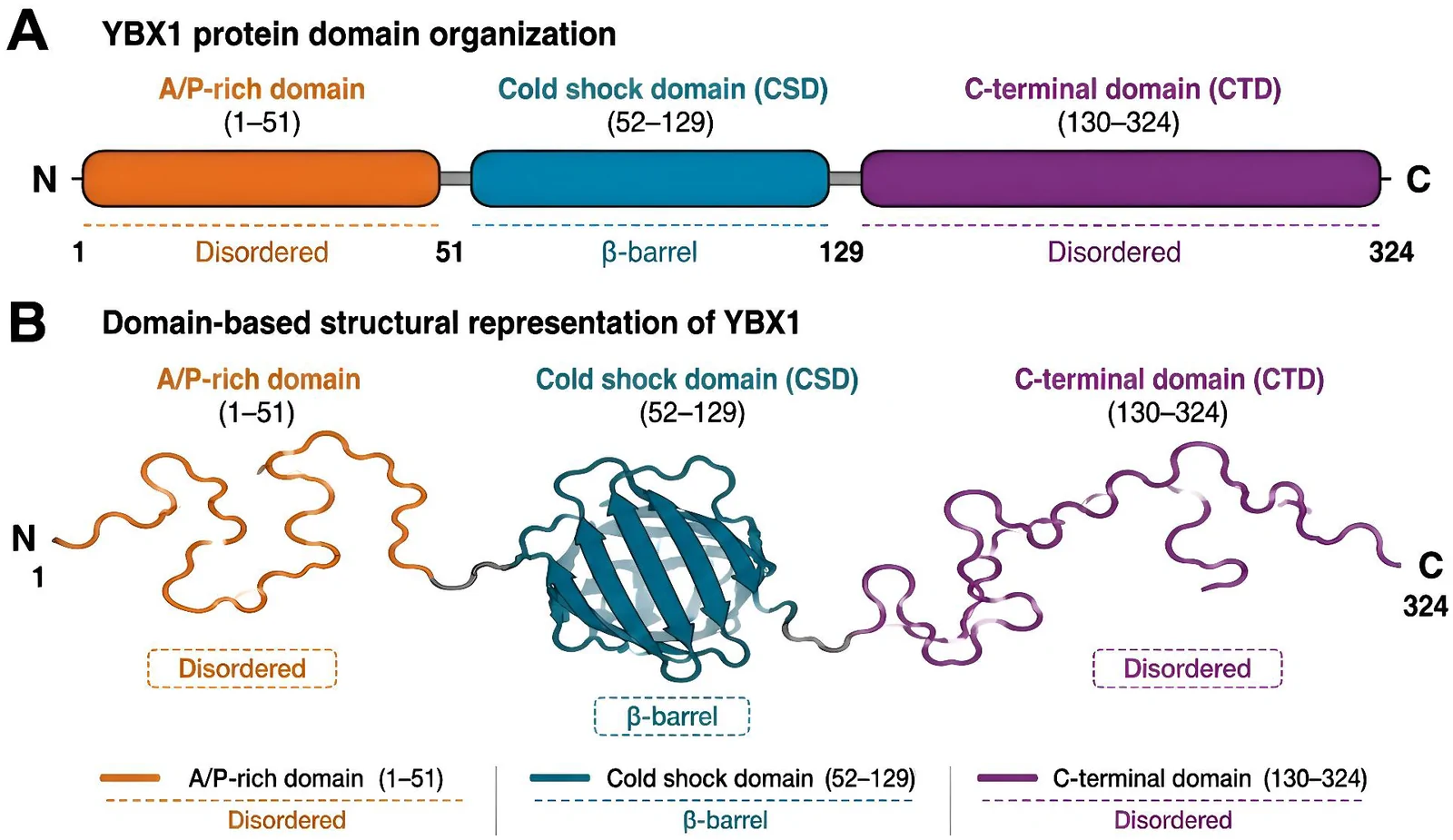
Domain architecture and structural features of human YBX1. **(A)** Schematic organization of YBX1 into three major regions: an N-terminal alanine/proline-rich domain (A/P-rich domain; residues 1–51), a central cold shock domain (CSD; residues 52–129), and a C-terminal domain (CTD; residues 130–324). **(B)** Structural representation showing the compact, folded CSD, which adopts a conserved OB-fold β-barrel architecture involved in RNA recognition, flanked by the predominantly intrinsically disordered A/P-rich and C-terminal regions. This modular arrangement supports sequence-selective RNA binding through the CSD while allowing the flanking domains to modulate YBX1 interactions, localization, and RNA-associated regulatory functions.

Among the RBPs, Y-box binding protein-1 (YBX1) has attracted particular interest, in part because it is overexpressed across a striking range of human cancers, and in part because of its multifunctional involvement in DNA- and RNA-dependent processes that drive tumor progression and portend poor outcome (17, 18). YBX1 is a modular RNA-binding protein composed of three functionally distinct regions: an N-terminal alanine/proline-rich (Ala/Pro-rich) low-complexity domain, a central cold shock domain (CSD), and a large, intrinsically disordered C-terminal domain (CTD) composed of alternating clusters of basic and acidic residues, the so-called charged-zipper or B/A repeats, which mediate protein–protein interaction, homo-multimerisation, and the liquid–liquid phase-separation behaviour discussed in Layers 1 and 2 (**Figure 1**) (19, 20). The CSD represents the principal RNA-recognition module and adopts an evolutionarily conserved oligonucleotide/oligosaccharide-binding (OB)-fold characterized by a five-stranded antiparallel β-barrel structure (21). While the CSD provides RNA-binding specificity, the flanking A/P-rich and C-terminal regions contribute to RNA-binding stability and mediate interactions with regulatory partners (22–24).

Depending on cellular context, YBX1 has been implicated in transcriptional regulation (25, 26), mRNA stabilization (27), translational control (28), the DNA-damage response (29), stress-granule dynamics (30), and epigenetic or epitranscriptomic regulation (31). It is perhaps not surprising, then, that aberrant YBX1 expression or activation associates with aggressive tumor behavior (32), metastatic progression (33), unfavorable prognosis (34), and resistance to a broad range of therapeutic modalities (25, 35). What is more difficult to reconcile is the sheer breadth of phenotypes attributed to a single protein: YBX1 has been reported to promote glycolytic adaptation (36), suppress ferroptosis through an m5C-dependent RNF115–DHODH regulatory axis (37), epithelial–mesenchymal transition (38), stemness (39), immune evasion (40), checkpoint regulation (41), and resistance to chemotherapy (25), targeted therapy (42), endocrine therapy (43), and immunotherapy (41). How so many apparently unrelated processes could converge on one RNA-binding protein remains, at present, an open question.

Existing reviews have tended to approach YBX1 from a pathway-centered or disease-specific vantage point (17, 25, 44), typically emphasizing a single signaling cascade or a particular tumor type. While these efforts have done much to advance the field, they leave two questions largely unanswered: how the many upstream signals converging on YBX1 are translated into a coherent, selective RNA regulatory program, and why YBX1, despite binding RNA broadly, ultimately controls only a limited subset of transcripts. A related body of evidence, spanning post-translational modification, ubiquitin-dependent turnover, subcellular trafficking, m5C-dependent RNA recognition, and non-coding RNA interactions, has likewise accumulated in largely separate literatures, with the result that no unifying framework has yet emerged.

Here, we attempt to bring these threads together by proposing a state-dependent framework in which YBX1 behaves not as a linear oncogenic effector but as an adaptive RNA hub, one whose output depends on which functional state it currently occupies. We organize the evidence into three interconnected layers. The first considers how post-translational modification, ubiquitin balance, and regulated intracellular trafficking define distinct functional states of YBX1. The second turns to transcript selection, asking how epitranscriptomic recognition and non-coding RNA-mediated regulation might explain why YBX1 favors certain adaptive RNA programs over others. The third, finally, considers how these selective RNA networks converge on the major phenotypic outputs of tumor adaptation, metabolic reprogramming, immune remodeling, cellular plasticity, and therapeutic resistance. In bringing together what has so far been a fragmented set of observations, we hope this Review offers not only a mechanistic account of YBX1-driven tumor adaptation but also a starting point for future biomarker discovery and therapeutic intervention.

## 2 Defining the adaptive RNA hub: criteria and boundary conditions

RNA-binding proteins are increasingly described as multifunctional, context-dependent regulators of RNA fate in cancer (12, 13), but the term “hub” is used loosely to denote functional breadth, which no observation could contradict. **Box 1** therefore constrains the term to four criteria, proposed as jointly necessary, and applies them to YBX1 alongside four other multifunctional RNA-binding proteins: IGF2BP1, HuR (ELAVL1), LIN28A and RBM39 (CAPERα).

Box 1

**Table:** Membership criteria for an adaptive RNA hub, applied to YBX1 and four comparator RNA-binding proteins (IGF2BP1, HuR/ELAVL1, LIN28A, RBM39/CAPERα).

Property	YBX1	IGF2BP1	HuR (ELAVL1)	LIN28A	RBM39 (CAPERα)
Criterion i — Combinatorial state encoding: several independent input classes set functional state					
Kinase-dependent phosphorylation	S102 (AKT/p70S6K/p90RSK) (46, 47); S165 (RIOK1) (49); PLK1-dependent phosphorylation affecting YBX1 nuclear localization (S174/S176) (48); S314 dephosphorylation (PPM1B) (50)	No comparably resolved multi-input code reported	Chk2, PKCα/δ, and p38-mediated phosphorylation, including p38-dependent Ser197 phosphorylation, regulates HuR shuttling (85–87)	No comparably resolved code reported	Primarily regulated through ligand-induced DCAF15-mediated degradation rather than reversible modification (92)
Metabolite-derived modification	K92 lactylation (p300/SIRT2) (51); K137 lactylation (53); T57 O-GlcNAc (36); GFPT2-driven O-GlcNAc (52)	Not established	Not established	Not established	Not established
Other covalent inputs	KAT7/PCAF acetylation (54, 55); R247 methylation relay (PRMT3/KDM4A) regulating YBX1 phase-separation properties (56); K26 deSUMOylation (SENP1) (57)	Not established	Not established	Not established	Not established
Ubiquitin chain editing setting abundance	K48 (TRIM29, FBXW11) (58, 59); K63 stabilizing (TRIM31) (60); K63 removal (BRCC36) (61)	Not established as a state code	Not established as a state code	Not established	Ligand-induced degradation via DCAF15 (92)
Identical abundance, different output?	Yes, K92 lactylation alters binding preference without changing abundance or localization (51); phosphorylation state is more prognostic than total protein (45)	Not established	Partially, localization-dependent activity (88)	Not established	Not established
Criterion ii — Extrinsic, conditional transcript selection					
Principal selection rule	Extrinsic: m5C marks deposited in trans by NSUN-family writers (62–68); plus ncRNA-mediated recruitment or interaction platforms (69–75)	Extrinsic: m6A marks, recognized through KH domains (89)	Intrinsic: AU-rich elements in 3′UTRs (11)	Intrinsic: structural elements and GGAG motif within let-7 precursor terminal loops (90)	Intrinsic: pre-mRNA splicing context and associated splice regulatory elements (92, 93)
Repertoire changes with cellular context?	Yes, reader output varies by tumor and by available writer/scaffold (41, 63, 94, 111, 113, 114)	Yes, m6A-dependent (89)	Limited, constitutive ARE recognition (11)	No, relatively switch-like inhibition of let-7 biogenesis (91)	Limited
Criterion iii — Dual-modality output across compartments					
Transcriptional output at DNA promoters	Yes, LDHA (80, 81); LDHB (82); HKDC1 (76); PD-L1 (43, 77, 78); HLA-E (40); CCL2 (79); YBX1-associated regulation of NOS2/ARG2 programs (153); RRM2 (54, 71)	Not established	Not established	Not established	Yes, coactivator for steroid hormone receptors (93)
Post-transcriptional output	Yes, stability and translation of STAT1 (41), RNF115 (31), QSOX1 (66), ATG9A (27), PFKFB4 (159)	Yes, m6A-dependent stabilization and storage (89)	Yes, ARE-dependent stability and translation (11)	Yes, let-7 biogenesis blockade (90, 91)	Yes, alternative pre-mRNA splicing (92, 93)
Both modalities documented?	Yes	No	No	No	Yes
Criterion iv — Closed-loop coupling to the phenotype generated					
Downstream product feeds back onto the regulator’s own state	Yes, lactate-associated metabolic signaling promotes YBX1 activation and LDHB expression (82); H3K18 lactylation is associated with increased YBX1 transcription (83); O-GlcNAc–glycolysis–H3K18la feedback (36); TROP2–YBX1–HIF-1α–lactate loop (84); uridine-derived K137 lactylation stabilizes YBX1 (53)	Not established	Not established	Not established	Not established
Summary characterization					
Best current description	State-dependent adaptive hub: conditional repertoire, dual modality, positioned inside the loop it regulates	Epitranscriptomic reader of fixed post-transcriptional modality	Constitutive ARE-directed stability regulator with signal-regulated localization	Specialized inhibitor of let-7 biogenesis; shares the cold-shock fold with YBX1 (90)	Splicing regulator and coactivator, defined pharmacologically by molecular-glue degradation

Four criteria are proposed as jointly necessary. No single criterion is unique to YBX1; the distinction rests on their conjunction, and in particular on the co-occurrence of criteria (iii) and (iv). Entries marked “not established” or “not reported” denote an absence of evidence and are deliberately uncited, since a negative cannot be sourced; in several cases this absence reflects the questions that have been asked of each protein rather than a demonstrated negative, and other RNA-binding proteins may satisfy additional criteria as their regulation is examined at comparable resolution. Two features of this comparison should be acknowledged. The criteria were derived inductively from the YBX1 literature and then applied to comparator proteins, which biases the exercise toward the protein that generated them; and the comparator set is small and chosen for breadth of function rather than sampled systematically. The claim is therefore not that YBX1 is unique among RNA-binding proteins, but that the conjunction of (iii) and (iv) is documented for YBX1 and not, at present, for the comparators examined — a claim that further work on any of these proteins could overturn.

Criterion (i) is combinatorial state encoding, in which several independent input classes set functional state: kinase- and phosphatase-controlled phosphorylation (45–50), metabolite-derived lactylation and O-GlcNAcylation (36, 51–53), acetylation, arginine methylation and deSUMOylation (54–57), and ubiquitin chain editing that sets abundance (58–61). Criterion (ii) is extrinsic, conditional transcript selection, in which targets are specified by m5C marks deposited in trans by NSUN-family writers (62–68) and by non-coding RNA scaffolds that reposition YBX1 onto particular promoters and transcripts (69–75). Criterion (iii) is dual-modality output, spanning transcriptional activity at DNA promoters (40, 43, 76–81) and post-transcriptional control of transcript stability and translation (27, 31, 41). Criterion (iv) is closed-loop coupling, in which lactate- and uridine-derived acyl modifications generated downstream of YBX1 return to set its own state (36, 53, 82–84).

No single criterion is unique to YBX1 (**Box 1**). HuR is regulated by several kinases (85–88) and by signal-dependent nucleocytoplasmic shuttling (88), but selects transcripts intrinsically, through AU-rich elements (11). IGF2BP1 selects extrinsically, through m6A rather than m5C, yet operates within a single post-transcriptional modality (89). LIN28A acts as a specialized inhibitor of let-7 biogenesis (90, 91), and RBM39 acts in both compartments but is set chiefly by ligand-induced degradation rather than by reversible modification (92, 93). Two features of this comparison should be acknowledged. The criteria were derived inductively from the YBX1 literature and then applied to comparator proteins, which biases the exercise toward the protein that generated them; and the comparator set is small and chosen for breadth of function rather than sampled systematically. The claim is therefore not that YBX1 is unique among RNA-binding proteins, but that the conjunction of (iii) and (iv) is documented for YBX1 and not, at present, for the comparators examined, a claim that further work on any of these proteins could overturn.

Two boundaries qualify this comparison: entries marked “not established” or “not reported” in **Box 1** denote an absence of evidence and are deliberately uncited, since a negative cannot be sourced, and in several cases that absence reflects the questions asked of each protein rather than a demonstrated negative (**Box 1**). The framework is nonetheless falsifiable, predicting that state-resolved readouts, phosphorylation (45), subcellular localization (47, 48), m5C-reader context (94) and dominant scaffold (74) will predict downstream circuit activity better than total YBX1 abundance. Should abundance prove equally informative, consistent with evidence that YBX1 expression chiefly marks proliferative state (32), the hub reduces to a promiscuous RNA-binding protein and the criteria assembled in **Box 1** describe a coincidence rather than an architecture.

## 3 The YBX1 paradox: one protein, many cancers, recurring biology

Few proteins in cancer biology are as conspicuous, or as conceptually unsettled, as YBX1. It surfaces in studies of head and neck epithelial plasticity (45), hepatocellular and pancreatic chemoresistance (95–97), glioblastoma myeloid regulation (98), pan-cancer immune infiltration (99, 100), and m5C-mediated post-transcriptional control (94, 98). Each report places YBX1, or its less-studied family members YBX2 and YBX3, at the center of its own story, yet the stories span domains that are not obviously connected: EMT, PI3K/mTOR signaling, PD-L1 biology, lipid metabolism, epitranscriptomic reading, myeloid polarization, and resistance to chemotherapy, targeted agents, and immune-checkpoint blockade. This recurrence is the paradox the review takes as its starting point.

Read together, the evidence converges. A pan-cancer analysis across 28 tumour types identifies YBX-family overexpression as an adverse prognostic marker in a majority of them (99), with tumour-specific analyses adding depth in hepatocellular carcinoma (95), renal (80) and brain (98) malignancies. Whether the association is genuinely strongest in these three lineages, or simply best studied in them, is not established by the available data. In hepatocellular carcinoma, this overexpression tracks with disrupted lipid and fatty-acid metabolism (95), T-cell dysfunction and immune exclusion (100), and persistent oncogenic kinase activity that drives chemoresistance (25). In head and neck cancer, however, total YBX1 is not the variable that matters: its phosphorylation state, set downstream of PI3K/mTOR, switches it between restraining basal-like proliferation and safeguarding mesenchymal-like cells against invasion, making phospho-YBX1 a more informative prognostic marker than total protein (45). Single-cell and spatial analyses in glioblastoma extend this state-dependence to the microenvironment, where YBX1 exerts opposing effects in distinct myeloid populations and shapes tumor–immune crosstalk through the NF-κB and TNF axes (98). Multi-omic studies of m5C readers reinforce the same logic at the molecular level: YBX1 and ALYREF act as transcript-selective readers that connect epitranscriptomic marks to immune-microenvironment composition and drug sensitivity across cancers (94). In pancreatic ductal adenocarcinoma, where chemotherapy remains the only option for most patients, YBX1 emerges as the convergence node where stemness, EMT, and drug-tolerance programs are coordinated (96).

**Figure 2:**
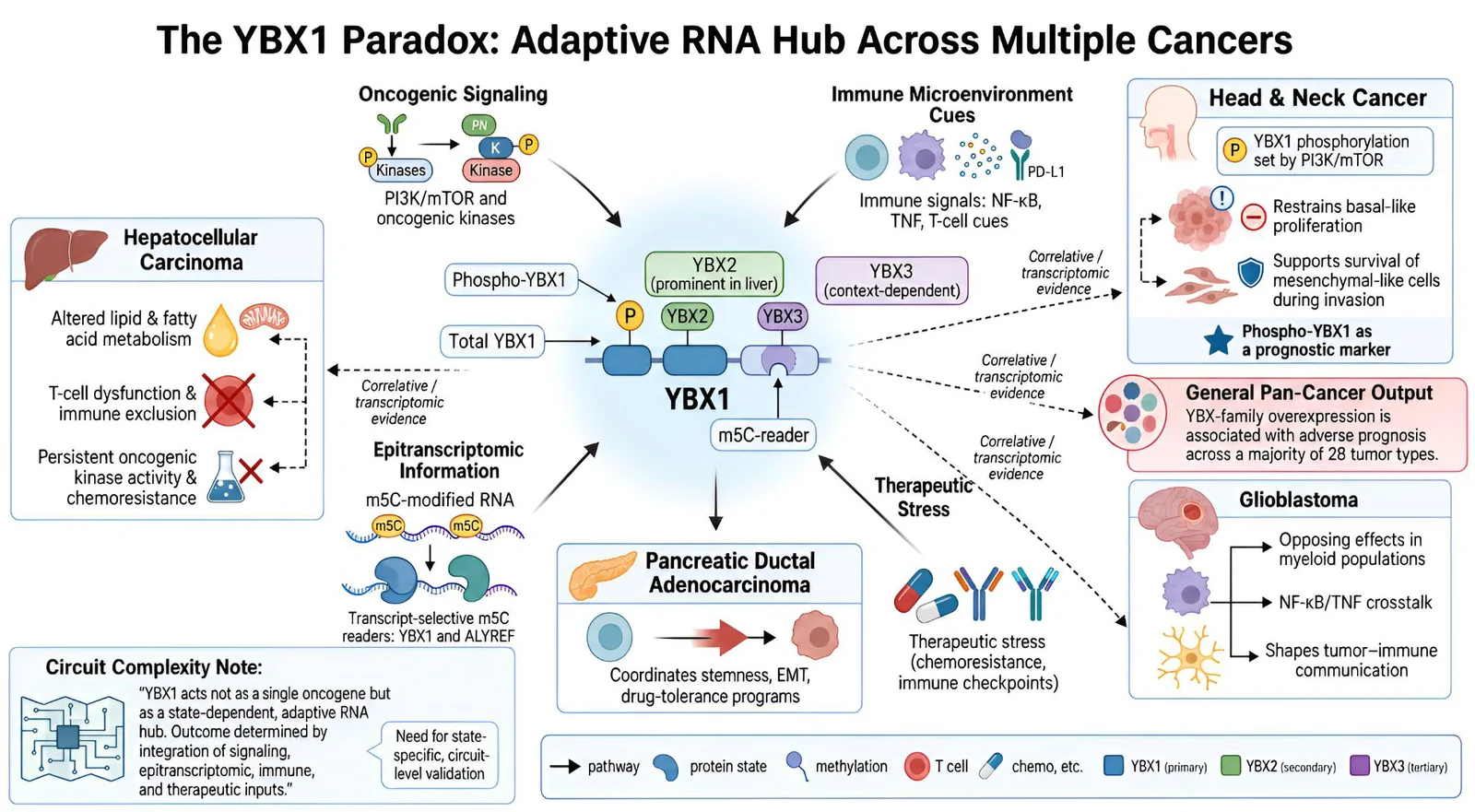
The YBX1 paradox: a context-dependent adaptive RNA hub across cancers. YBX1 integrates oncogenic signaling, m5C-associated epitranscriptomic information, immune microenvironment cues, and therapeutic stress into state-specific RNA regulatory programs. Depending on tumor context, YBX1 family members (YBX1–3) coordinate distinct outputs including metabolic rewiring, immune modulation, epithelial plasticity, stemness, and therapy resistance. Current evidence supports a circuit-based model in which YBX activity and clinical impact depend on cellular state, tumor type, and regulatory context, highlighting the need for state-specific functional validation.

These observations resist a conventional oncogene framing in which one gene drives one pathway towards one phenotype. YBX1 is too multifunctional, too context-sensitive, and too tightly state-regulated for that model. We propose instead that YBX1 is best understood as an adaptive RNA hub, in the restricted sense defined in Section 2 (**Box 1**): a convergence point at which oncogenic signalling (45, 97), epitranscriptomic information (94, 98), immune-microenvironment cues (98–100), and therapeutic stress (96, 97) are integrated into transcript-selective decisions that produce coordinated metabolic, immune, and resistance phenotypes (**Figure 2**). Read this way, the apparent diffuseness of the YBX1 literature is not noise; it is the expected signature of a hub whose outputs vary with which inputs are active.

Two caveats temper this synthesis. First, much of the pan-cancer and immune-infiltration evidence rests on bulk-transcriptomic correlation and database-derived inference (94, 95, 100), which establish association rather than directionality. Second, tumor-type specificity is genuine and extends to family-member identity: YBX2 emerges as the more informative family member in liver cancer (99), and few studies yet resolve which post-translational state of YBX1 is active in which patient. State-specific, circuit-level functional validation therefore remains the field’s central methodological gap. The clinically actionable question is not whether YBX1 is high or low, but which YBX1-dependent circuit a given tumor is using, provided that question can be answered with state-resolved rather than abundance-based measurements. To begin answering that question, we must first ask how YBX1 itself is regulated and switched between distinct functional states, the subject of Layer 1.

## 4 Layer 1: the YBX1 state code: post-translational modifications and partner-driven regulation

If YBX1 is best understood as an adaptive RNA hub, the first mechanistic question is how its functional state is set. The evidence assembled here supports the view that YBX1 is not a single molecule with a fixed activity but a state-switching protein, whose behavior in a given tumor depends on a layered set of regulatory inputs: covalent post-translational modifications (PTMs) that adjust intrinsic activity, a ubiquitin-balance system that sets abundance, and a localization and partner network that determines where YBX1 acts. We summarize these regulatory inputs as Layer 1 of the framework (**Table 1**).

**Table 1:** Layer 1: the YBX1 state code.

Specific event/modification	Writer · eraser · enzyme/partner	Effect on YBX1 (activity · abundance · localization) and reported consequence	Tumor type (experimental context)	Ref.
1A: Covalent post-translational modifications (set intrinsic activity and RNA-binding)				
Phosphorylation of YBX1 (ROS-induced)	AKT, ERK, RSK; reversed by RSK inhibitor BI-D1870	Activity — phospho- YBX1 is the proximal driver of ↑ migration and ↑ PD-L1/PD-L2	Pleural mesothelioma (cell models)	(101)
Lactylation at K92	Writer p300; eraser SIRT2; partner G3BP2	RNA-binding — ↑ association with m5C-modified transcripts; little change in abundance or localization	Colorectal cancer	(51)
O-GlcNAcylation at T57	O-GlcNAc modification (hexosamine pathway–linked)	Abundance + Localization — stabilizes YBX1 and promotes S102 phosphorylation → nuclear translocation; feeds glycolysis–H3K18la loop	Hepatocellular carcinoma	(36)
O-GlcNAcylation	GFPT2 (rate-limiting hexosamine-pathway enzyme)	Localization — nuclear translocation; YBX1 acts as TF for IL-18 → M2 macrophage polarization	Pancreatic cancer	(52)
Acetylation	KAT7 (E508 required for binding)	Activity, ↑ transcriptional activity at RRM2 and TK1 (nucleotide-metabolism reprogramming)	Hepatocellular carcinoma	(54)
Acetyltransferase route (indirect): PCAF → Twist1-dependent YBX1 expression	PCAF (acetylates Twist1)	Abundance, regulates YBX1 expression; knockdown re-sensitizes to cisplatin/doxorubicin but not 5-FU	Urothelial cancer (drug-resistant)	(55)
Arginine methylation at R247 (write–erase relay)	Writer PRMT3; eraser KDM4A; Acipimox, a candidate PRMT3 inhibitor (tool hit; PRMT3 selectivity not independently validated)	Localization via phase separation, methylation suppresses cytoplasmic LLPS → nuclear import; nuclear demethylation restores LLPS → transcription of ABC transporters & HR-repair genes	Rectal cancer	(56)
deSUMOylation at K26 (SUMO removal)	SENP1 (SUMO protease); partner DDX5	Activity — preserves the YBX1–DDX5 interaction and AKT activation	Colorectal cancer	(57)
1B: Ubiquitin balance (sets abundance)				
Ubiquitination → proteasomal degradation	E3 ligase TRIM29	Abundance ↓ — inhibits PI3K/AKT and restores lenvatinib sensitivity; higher TRIM29 = longer OS	Hepatocellular carcinoma	(58)
K48-linked polyubiquitination → degradation	E3 ligase FBXW11 (binds cold-shock domain)	Abundance ↓ — suppresses the AKT/mTOR axis	Hepatocellular carcinoma	(59)
K63-linked polyubiquitination at K81 (non-degradative)	E3 ligase TRIM31	Abundance ↑ — stabilizes YBX1 (NF-κB/P65 feedback); chain type, not ubiquitination per se, sets the outcome	Colorectal cancer	(60)
ncRNA-guided ubiquitination → degradation	circNEIL3 recruits E3 ligase Nedd4L	Abundance ↓ — anti-metastatic across several cancer types *in vitro*/*in vivo*	Colorectal cancer (multi-cancer models)	(102)
ncRNA-mediated suppression of poly-ubiquitination	lnc-SOX9-4 (nuclear)	Abundance ↑ — stabilizes YBX1; supports proliferation and migration	Colorectal cancer	(103)
Partner-mediated inhibition of ubiquitination	Paxillin (PXN); STAT3–PXN–SRC feedback loop	Abundance ↑ — stabilizes YBX1 by withholding ubiquitin marks	Glioblastoma, IDH-wildtype	(104)
Deubiquitination → stabilization	DUB USP21; inhibitor BAY-805	Abundance ↑ — drives HIF1A transcription	Prostate cancer	(105)
Deubiquitination → stabilization (acts on YBX3, not YBX1)	DUB USP18	Abundance ↑ (YBX3) — activates PI3K/AKT; family-member evidence, not YBX1 itself	Clear cell renal cell carcinoma	(106)
1C: Localization and partner-driven trafficking (set where YBX1 acts)				
Phosphorylation at S102	AKT, p70S6K, p90RSK; inhibitors TAS0612 + everolimus	Localization — nuclear pYBX1 drives drug-resistance genes; pYBX1 enriched in TNBC	Breast cancer (antiestrogen-resistant; TNBC)	(46)
S102 phosphorylation facilitated by an RNA scaffold	circIPO7 (cytoplasmic); AKT	Localization — nuclear translocation → FGFR1/TNC/NTRK1; cisplatin resistance and metastasis	Nasopharyngeal carcinoma	(47)
Phosphorylation at S174/S176 (distinct from the AKT axis)	PLK1	Localization — nuclear translocation; PLK1 inhibition → ↓ nuclear YBX1, DNA damage, apoptosis	Glioma stem cells	(48)
Partner-mediated nuclear import	Karyopherin KPNB1 (stabilized by DUB USP7)	Localization — nuclear import → transactivates NLGN3 (USP7 → KPNB1 → YBX1 → NLGN3)	Glioblastoma	(107)
Partner-mediated nuclear translocation	Menin	Localization — nuclear translocation → transactivates HKDC1 (glycolysis)	Pancreatic ductal adenocarcinoma	(76)

Regulatory inputs that set YBX1 functional state, presented in three panels by mechanism class. (1A) Covalent post-translational modifications, which set intrinsic activity and RNA-binding preference. (1B) Ubiquitin balance, which sets abundance. (1C) Localization and partner-driven trafficking, which set where YBX1 acts. Arrows denote direction of change (↑ increase, ↓ decrease).

### 4.1 Covalent modifications set YBX1’s intrinsic activity

The most consistent observation across this group of studies is that PTMs change *what YBX1 can do*, not only how much of it is present. Phosphorylation remains the best-characterized example. In pleural mesothelioma, xanthine/xanthine-oxidase-induced reactive oxygen species (ROS) increase AKT, ERK and RSK activity together with YBX1 phosphorylation, and these changes are accompanied by elevated PD-L1 and PD-L2 expression and enhanced migration; the RSK inhibitor BI-D1870, which reduces YBX1 S102 phosphorylation indirectly, reverses these effects more completely than AKT or MEK inhibition alone (101). This is consistent with the possibility that the phosphorylation state of YBX1, rather than ROS or upstream kinase activity per se, may be the proximal regulator of these phenotypes, although this rests on a single pharmacological model.

A second class of modifications appears to fine-tune RNA recognition rather than abundance. Lysine 92 (K92) lactylation of YBX1, installed by p300 and removed by SIRT2, has little effect on YBX1 stability or localization but significantly enhances its association with m5C-modified transcripts, with G3BP2 acting as a preferential partner of the lactylated form in colorectal cancer (51). This implies that PTMs can alter YBX1’s binding preferences without changing its abundance, raising the possibility that two tumors with similar YBX1 levels may handle the m5C-marked transcriptome differently.

O-GlcNAcylation links metabolism to YBX1 state. In hepatocellular carcinoma, O-GlcNAcylation at threonine 57 (T57) stabilizes YBX1 and also promotes its serine 102 (S102) phosphorylation and nuclear translocation, supporting the transcription of glycolysis-related genes; the resulting lactate output feeds back via H3K18 lactylation onto YBX1 transcription itself, generating a positive feedback loop (36). In pancreatic cancer, GFPT2, the rate-limiting enzyme of the hexosamine biosynthesis pathway, drives O-GlcNAcylation and nuclear translocation of YBX1, which then acts as a transcription factor for IL-18 and contributes to M2 macrophage polarization (52). Read together, these studies position O-GlcNAcylation as a metabolic input that places YBX1 in the nucleus.

Acetylation provides a third route. KAT7 acetylates YBX1 in HCC and enhances its transcriptional activity at the promoters of ribonucleotide reductase M2 (RRM2) and thymidine kinase 1 (TK1), linking YBX1 to nucleotide metabolism reprogramming (54). Independently, P300/CBP-associated factor (PCAF) regulates YBX1 expression in a Twist1-dependent manner in cisplatin- and doxorubicin-resistant urothelial cancer cells, and PCAF knockdown re-sensitizes cells to these agents but not to 5-fluorouracil (55). These observations support the possibility that distinct acetyltransferases shape different YBX1-dependent transcriptional programs.

Arginine methylation introduces an additional layer of compartmental control. In rectal cancer, PRMT3 methylates YBX1, which suppresses its cytoplasmic phase separation and is required for nuclear translocation; KDM4A then demethylates YBX1 at the R247 residue inside the nucleus, where phase separation re-emerges and supports transcription of ABC transporters and homologous-recombination repair genes implicated in chemoradiotherapy resistance (56). In that study, the anti-lipolytic agent acipimox was identified by repurposing screen as a candidate PRMT3 inhibitor and chemoradiotherapy sensitiser; its selectivity for PRMT3 over other arginine methyltransferases has not been independently established, and it should be regarded as a tool hit rather than a validated PRMT3 probe. The write/erase cycle is itself the regulatory unit, not any single methylation event.

Finally, SUMOylation acts in the opposite direction from many of these modifications: removal, not addition, is the key regulated event. SENP1-mediated deSUMOylation of YBX1 at K26 preserves the YBX1–DDX5 interaction and supports AKT activation in colorectal cancer, with elevated SENP1 and YBX1 levels associated with poorer outcomes (57).

Taken together, these eight studies suggest that PTMs modulate YBX1 along several distinct axes: RNA binding preference (51), metabolic responsiveness (36, 52), transcriptional activity at specific promoters (54, 55), compartmentalization through phase separation (56), and partner-mediated kinase coupling (57, 101). Different tumor types appear to emphasize different PTM combinations rather than converge on a single dominant modification (**Table 1A**).

### 4.2 Ubiquitin balance determines YBX1 abundance

Beyond covalent modification, YBX1 abundance is set by a competition between E3 ubiquitin ligases that direct it for degradation and deubiquitinases (DUBs) that protect it. The evidence does not support a single dominant ligase or DUB; instead, the balance appears tumor-type-specific and, in some cases, context-dependent within a single tumor type. Among the E3 ligases, the TRIM family illustrates the heterogeneity well. In hepatocellular carcinoma, TRIM29 and FBXW11 both direct YBX1 to proteasomal degradation, suppressing PI3K/AKT and AKT/mTOR signaling, respectively, and, for TRIM29, restoring lenvatinib sensitivity (58, 59). In contrast, TRIM31 in colorectal cancer catalyzes K63-linked polyubiquitination at K81, which stabilizes rather than degrades YBX1 and drives inflammation-linked carcinogenesis through an NF-κB/P65 feedback loop (60). These observations are consistent with the broader principle that ubiquitin chain type, not ubiquitination per se, determines functional outcome, and they argue against treating any single TRIM protein as a universal regulator. This principle requires one qualification, since chain type alone is not fully determining: the same K63 linkage can stabilize YBX1 here yet, elsewhere, mark it for clearance; in hepatocellular carcinoma, BRCC36-mediated removal of K63 chains shields YBX1 from p62-dependent selective autophagy (61). The two outcomes are reconcilable once chain type is read together with the modified site (K81 versus alternative lysines), the availability of chain-recognizing receptors such as p62, and the prevailing metabolic state (for example, AMPK/mTOR signaling); it is this combination, rather than the K63 linkage in isolation, that sets whether a given chain stabilizes or degrades YBX1.

Non-coding RNAs participate directly in this balance. circNEIL3 binds YBX1 in colorectal cancer and recruits Nedd4L to promote YBX1 proteasomal degradation, with anti-metastatic effects across multiple cancer types *in vitro* and *in vivo* (102). lnc-SOX9-4, also in colorectal cancer, acts in the opposite direction: it binds YBX1 in the nucleus and suppresses its poly-ubiquitination, stabilizing the protein and supporting proliferation and migration (103). The same disease therefore contains ncRNAs that shift the ubiquitin balance in opposite directions, depending on cellular context.

Partner proteins can also stabilize YBX1 independently of E3 ligases. In IDH-wildtype glioblastoma, paxillin (PXN) stabilizes YBX1 by inhibiting its ubiquitination, and PXN itself is regulated by a STAT3-PXN positive feedback loop in which STAT3 transcriptionally upregulates PXN and PXN reciprocally activates STAT3 through SRC (104). This raises the possibility that, in some tumors, YBX1 abundance is maintained primarily by withholding ubiquitin marks rather than by adding them.

DUBs complete the picture. USP21 in prostate cancer deubiquitinates and stabilizes YBX1, with downstream upregulation of HIF1-α transcription; and the selective USP21 chemical probe BAY-805 has been used to interrogate this axis. BAY-805 is characterised as an *in vitro* probe rather than a lead compound, and any *in vivo* effect reported in this setting (105) should be interpreted as target-validation evidence rather than as pharmacological proof of concept. USP18 in clear-cell renal cell carcinoma operates on YBX3 rather than YBX1, deubiquitinating and stabilizing YBX3 with downstream activation of PI3K/AKT (106), a useful reminder that the Y-box family is broader than YBX1 alone and that ubiquitin regulation extends to other family members in selected contexts.

Across these studies, three patterns emerge. First, opposing E3 ligases act on YBX1 in different tumors, and even within a single tumor type the direction of regulation can differ depending on the ligase or chain type (58–60). Second, ncRNAs and partner proteins are integral to ubiquitin balance, not peripheral (102–104). Third, deubiquitinases provide an independent stabilization route that may dominate in selected contexts (105, 106). Total YBX1 abundance in any one tumor is therefore a net output of these competing inputs (**Table 1B**).

### 4.3 Localization and partner-driven trafficking control where YBX1 acts

Activity and abundance are not sufficient to define YBX1’s functional state; localization is the final input. These studies describe how phosphorylation by specific kinases and partner-mediated nuclear import together govern the cytoplasmic-versus-nuclear distribution of YBX1 and, through that distribution, which downstream programs YBX1 can engage.

Phosphorylation routes to the nucleus are well represented. S102 phosphorylation, set downstream of AKT, p70S6K and p90RSK, is the canonical signal: in antiestrogen-resistant breast cancer, phospho-S102 YBX1 accumulates in the nucleus and supports drug-resistance gene programs, and combined treatment with the multikinase inhibitor TAS0612 and the mTORC1 inhibitor everolimus suppresses phospho-YBX1 and overcomes resistance both *in vitro* and *in vivo*; phospho-YBX1 is also enriched in triple-negative breast cancer compared with other subtypes (46). RNA scaffolds can facilitate phosphorylation at S102: in nasopharyngeal carcinoma, circIPO7 binds YBX1 in the cytoplasm and promotes its AKT-mediated S102 phosphorylation, after which nuclear YBX1 activates FGFR1, TNC and NTRK1 transcription and contributes to cisplatin chemoresistance and distant metastasis (47). Phosphorylation is not restricted to S102. In glioma stem cells, PLK1 phosphorylates YBX1 at serines 174 and 176, distinct from the AKT axis, and these modifications also drive nuclear translocation; PLK1 inhibition reduces nuclear YBX1, induces DNA damage, and triggers apoptosis (48). The same outcome (nuclear translocation) can therefore be reached through more than one phosphorylation site and kinase, depending on cell state.

Partner-mediated import provides a parallel route. In glioblastoma, KPNB1 interacts with YBX1 and promotes its nuclear translocation. Nuclear YBX1 subsequently binds the NLGN3 promoter and increases NLGN3 expression, thereby supporting glioma cell growth. USP7 stabilizes KPNB1 through deubiquitination, collectively establishing a USP7–KPNB1–YBX1–NLGN3 regulatory pathway (107). In pancreatic ductal adenocarcinoma, Menin interacts with YBX1 and facilitates its nuclear translocation, where YBX1 transactivates HKDC1 and contributes to glycolysis (76). These two examples are consistent with a broader pattern in which partner proteins serve as nuclear-import cofactors and link YBX1’s nuclear function to specific transcriptional programs.

Cross-talk with the modifications described in Section 4.1 is also evident. O-GlcNAcylation of YBX1 at T57 not only stabilizes the protein but promotes S102 phosphorylation and nuclear translocation (36), and PRMT3-mediated arginine methylation suppresses cytoplasmic phase separation as a prerequisite for nuclear entry, with KDM4A enabling phase separation in the nucleus (56). Different PTM and partner inputs therefore appear to converge on nuclear translocation as a shared decision point.

Across these studies, with additional evidence from (36) and (56), localization control emerges as the funnel through which Layer 1 outputs commit YBX1 to specific nuclear transcriptional programs. Two implications follow. First, measurements of total YBX1 abundance may misrepresent its functional state when nuclear-cytoplasmic distribution is not assessed in parallel. Second, distinct upstream inputs, kinases, karyopherins, scaffold RNAs, and partner proteins can produce convergent nuclear YBX1 outputs in different tumors, consistent with the framing of YBX1 as a state-dependent integrator rather than a linear oncogene (**Table 1C**).

## 5 Layer 2: the YBX1 RNA program: epitranscriptomic reading and ncRNA-coordinated transcript fate

### 5.1 The transcript-selection problem: why YBX1 does not regulate all RNAs equally

Once YBX1 is placed into a particular functional state by the Layer 1 inputs discussed above, the next question is what it then does. In principle, an abundant RNA-binding protein could engage transcripts broadly; in practice, the evidence supports a more restricted picture, in which YBX1-associated RNA regulation appears transcript-selective rather than uniform; the mechanisms by which YBX1, YBX3, and ALYREF are connected to a restricted transcript set are summarized in **Table 2**, whose three panels correspond to the mechanisms developed in Sections 5.2, 5.3 and 5.5: m5C writer–reader recognition (**Table 2A**), ncRNA control of YBX1 itself (**Table 2B**), and ncRNA scaffolding of YBX1 onto targets (**Table 2C**). Across cancer contexts, the same protein is reported to stabilize, translate, or scaffold a discrete subset of mRNAs whose products concentrate in a small number of adaptive programs.

**Table 2:** Layer 2: the YBX1 RNA program.

Reader (Y-box/m5C reader)	Writer/ncRNA	Target/regulated molecule	Fate of target	Tumor type (context)	Ref.
2A: m5C writer–reader recognition (reader interprets m5C-marked transcripts)					
YBX1	m5C (writer not specified)	STAT1 mRNA	↑ stability/translation → sustains STAT1/PD-L1 pathway	Intrahepatic cholangiocarcinoma	(41)
YBX1	Writer NSUN2	PHGDH mRNA (3′UTR)	↑ mRNA stability/half-life	High-grade B-cell lymphoma (double-hit)	(62) P
ALYREF + YBX1 (ALYREF first; YBX1 binds transitive m5C)	Writer NSUN2	YAP mRNA	↑ stability + translation (impedes AGO2 binding)	NSCLC	(63)
YBX1	m5C (writer not specified)	RNF115 mRNA (3′UTR)	↑ stability + translation	Hepatocellular carcinoma	(31, 37)
YBX1	m5C (writer not specified)	TM4SF1 mRNA	↑ stability	Bladder cancer	(108)
YBX1 + ALYREF	m5C (writer not specified)	No defined target †	Associative only, expression correlated with HIF1-α and migration †	Gastric cancer	(109)
m5C regulators incl. YBX1/ALYREF †	m5C-regulator set (cohort)	No defined target — cohort signature †	Prognostic/therapy-response signature; no direct mechanism †	Pancreatic ductal adenocarcinoma	(110)
YBX3 (recruits PABPC1)	Writer NSUN2 (LMP1/NF-κB-induced)	ICAM-1 mRNA	↑ translation	Nasopharyngeal carcinoma (EBV+)	(64)
YBX1	Writer NSUN2	CCT5 mRNA	↑ stability	Lung adenocarcinoma	(65)
YBX1	Writer NSUN2	QSOX1 mRNA (CDS)	↑ translation → intrinsic gefitinib resistance	EGFR-mutant NSCLC	(66)
YBX1	Writer NSUN2	HGH1 mRNA	↑ stability + protein synthesis	Breast cancer	(67)
YBX1	Exosomal NSUN2 (m5C)	PD-L1 (PD-L1) mRNA	↑ stability	Diffuse large B-cell lymphoma	(68)
YBX1 (partner MATR3)	m5C (writer not specified)	CCNL2 mRNA	↑ stability	Ovarian cancer (cisplatin resistance)	(113)
YBX1 (recruits PABPC1)	m5C (writer not specified)	CHD3 mRNA	↑ stability → homologous-recombination repair	Ovarian cancer (platinum-resistant)	(114)
YBX1 (W65 residue critical)	m5C (writer not specified)	SESN2 mRNA	↑ stability	Multiple myeloma (PI-resistant)	(111) P
ALYREF (recruits NSUN2)	Writer NSUN2	lncRNA CASC19 (3′UTR)	↑ stability (→ HNRNPC/SCD)	Colorectal cancer	(115)
2B: Non-coding and viral RNAs that regulate, or are engaged by, YBX1 outside the m5C-on-mRNA code					
YBX1 (regulated)	circRNA hsa_circ_0007990	YBX1 protein (the reader itself)	↑ abundance — blocks ubiquitin/proteasome degradation	Breast cancer	(116)
YBX1 (regulated)	circRNA-SORE (exosome-transmitted)	YBX1 protein	↑ abundance — blocks PRP19-mediated ubiquitination	Hepatocellular carcinoma (sorafenib resistance)	(117)
YBX1 (regulated)	circZNF79(5) (recruits K63-DUB BRCC36)	YBX1 protein	↑ abundance — shields from p62 autophagic degradation (AMPK/mTOR)	Hepatocellular carcinoma	(61)
YBX1 (regulated)	piRNA piR-YBX1	YBX1 mRNA	↓ abundance — tumor-suppressive counterexample (↓ MAPK)	Triple-negative breast cancer	(118)
— (no direct YBX1 mechanism) ‡	tRF-3017b	Not established ‡	No direct YBX1-regulatory mechanism shown; cited as parallel (cytokine/STAT3) ‡	Prostate cancer	(119)
YBX1 (m5C reader of ncRNA)	Y3-derived ysRNA (m5C; exosome-transmitted)	Y3-derived ysRNA (non-coding)	Localization at DNA double-strand breaks; modulates PARP1 residency	DNA-damage/radioprotection model (not tumor-type-specific)	(120)
YBX1 (cold-shock domain W65/F74/F85)	Oncolytic virus M1 RNA (viral)	OVM viral RNA (NSP4–capsid junction, 3′UTRs)	Sustains viral RNA synthesis; YBX1 as host proviral factor/efficacy biomarker	Multiple tumor models (oncolytic-virus context)	(121)
2C: ncRNA scaffolds directing YBX1 to target transcripts					
YBX1	circEPHB4	MRPS16 mRNA	↑ stability via YBX1-mediated m5C (also limits YBX1 ubiquitination — dual)	Glioma	(70)
YBX1 (nuclear)	Super-enhancer-driven circPVT1	RRM2	Transcriptional activation (YBX1 recruited to nucleus)	Hepatocellular carcinoma	(71)
YBX1	circPOLD1	No defined target — AKT/mTOR/HIF-1α pathway †	Biomarker-level association (AUC 0.951 LSIL+); mechanism warrants validation †	Cervical lesions/cervical cancer	(72)
YBX1	lncRNA USP2-AS1 (hypoxia)	HIF1-α mRNA	↑ translation (↑ HIF1-α protein)	Hepatocellular carcinoma	(73)
YBX1	lncRNA DARS1-AS1	E2F1, CCND1, FOXM1 mRNAs	↑ stability (G1–S, HR repair, radioresistance)	Glioblastoma (stem-like cells)	(74)
YBX1	lncRNA SBF2-AS1	SBF2-AS1 (reciprocal feedback)	YBX1–SBF2-AS1 positive feedback sustaining PI3K/AKT/mTOR	Breast cancer (tamoxifen)	(75)
YBX1 (with IGF2BP1)	m6A-modified LINC02418 (METTL3) — m6A on lncRNA, not m5C-on-mRNA	CTNNB1 (promoter + mRNA)	Transcriptional activation (YBX1→promoter) + mRNA stability (IGF2BP1)	Colorectal cancer	(69)

Transcript-selection mechanisms, presented in three panels. (2A) m5C writer–reader recognition, in which YBX1, YBX3, or ALYREF interpret NSUN-deposited marks on target transcripts. (2B) Non-coding RNAs acting on YBX1 itself to set its abundance or availability. (2C) Non-coding RNAs acting as scaffolds that direct YBX1 to specific targets or promoters. Symbols: † association- or signature-level evidence without a defined direct target; ‡ cited as parallel evidence, no direct YBX1 mechanism established; ↑ increase, ↓ decrease. P denotes preliminary evidence reported only in a conference abstract and not yet supported by a full peer-reviewed publication.

Three recurrent themes are visible across the studies assembled here. The first is immune escape: in intrahepatic cholangiocarcinoma, YBX1 acts as an m5C reader that stabilizes STAT1 transcripts and supports PD-L1 expression, with measurable effects on antitumor T-cell responses (41); in high-grade B-cell lymphoma, NSUN2 and YBX1 have been reported to co-stabilise PHGDH mRNA, with proposed downstream effects on PD-L1 expression via PAX5 and on macrophage recruitment via CCL2 (62). However, this evidence is currently limited to a conference abstract, and the proposed mechanistic relationship linking PHGDH, PAX5, and PD-L1 awaits confirmation in a peer-reviewed study. The second is growth and metastasis signaling: in non-small-cell lung cancer, an ALYREF–YBX1 module reads m5C marks on YAP mRNA and feeds a NSUN2–YAP positive feedback loop (63); in hepatocellular carcinoma, YBX1 binds m5C-modified motifs in the 3′UTR of RNF115, enhancing its stability and translation and supporting PI3K/AKT activation (31). The third is metabolic and migratory rewiring: m5C-dependent YBX1 stabilization of TM4SF1 has been linked to bladder cancer proliferation and glycolysis (108), and YBX1/ALYREF expression correlates with HIF1-α and with migratory phenotype in gastric cancer (109). At the cohort level, an m5C-regulator-associated transcriptional signature predicts prognosis, therapy response, and immune-exclusion status in pancreatic cancer (110).

These observations support the possibility that YBX1 does not regulate all RNAs equally. Its reported preferred targets cluster around immune-checkpoint stabilization, proliferation-and-metastasis signaling, metabolic adaptation, and treatment response, the same phenotypic outputs that Layer 3 will later address. The strength of evidence is uneven: direct binding and stability data in some studies, signature-level or correlational inference in others (see evidence markers in **Table 2A**), and the molecular rules underlying selection are unlikely to be identical across contexts. What the data do support is that selection, not abundance, not activity in the abstract, is the central problem of Layer 2, and that any account of YBX1 in cancer must explain how, among many possible RNAs, a particular subset is preserved. This selective regulation encompasses three mechanistically non-equivalent outputs, which are not interchangeable: (i) post-transcriptional stabilization of existing mRNAs through cytoplasmic m5C reading; (ii) translational regulation via reader–ribosome interactions; and (iii) nuclear transcriptional activation at DNA promoters, for example at LDHB (82), LDHA (80, 81) and HKDC1 (76), which increases nascent RNA output and is not attributable to cytoplasmic m5C reading. Only (i) and (ii) follow from the m5C writer–reader code developed below. Where this review refers to YBX1’s outputs collectively as transcript-selective, the term therefore denotes a composite of post-transcriptional and transcriptional RNA-fate decisions, not a single post-transcriptional mechanism. The next subsection examines the best-characterized component of that selection logic: the m5C writer–reader system, in which NSUN-family methyltransferases and YBX1/ALYREF readers appear to constitute one major rule by which YBX1-dependent transcripts are picked out.

### 5.2 The m5C writer–reader axis: NSUN-family writers, ALYREF, YBX1 and YBX3

YBX1 is an abundant RNA-binding protein, but the cancer phenotypes attributed to it imply selective rather than uniform RNA engagement. The evidence assembled here supports the view that 5-methylcytosine (m5C) provides one major selection rule: NSUN-family writers install or shape m5C marks on a defined subset of transcripts, and ALYREF, YBX1, and YBX3 then interpret those marks. This writer–reader system does not exhaust YBX1 biology, but it offers one mechanistically tractable code by which malignancy-relevant RNAs are picked out for downstream handling. It should be emphasized, however, that the designation of YBX1 as a bona fide m5C reader rests on indirect evidence. The available data, RIP/MeRIP co-enrichment of m5C-marked transcripts, and W65-dependent binding (for example, of SESN2 (111)), are consistent with selective m5C recognition but do not establish it: no crystal structure, ITC, or SPR measurement yet demonstrates that the YBX1 cold-shock domain binds m5C-modified RNA with higher affinity than the identical unmodified sequence. The m5C “selection rule” is therefore best read as a working hypothesis with strong functional support rather than a biochemically resolved mechanism, particularly by contrast with ALYREF, for which m5C reading has more direct structural backing (112). On the writer side, NSUN2 is the most consistently implicated enzyme, upregulated across nasopharyngeal, lymphoid, lung, and breast malignancies, and in one case transferred exosomally, a route that extends m5C deposition beyond cell-autonomous regulation (63–68). The marked transcripts are heterogeneous across these settings: ICAM-1 (64), PHGDH (62), YAP (63), CCT5 (65) and QSOX1 (66), HGH1 (67), and PD-L1 via exosomal NSUN2 (68), yet they cluster within a defined set of malignancy-relevant programs rather than spreading across the transcriptome at large.

The reader side is functionally heterogeneous. YBX1 acts as a cytoplasmic m5C reader that stabilizes STAT1 in cholangiocarcinoma (41), RNF115 in HCC (31), CCNL2 in ovarian cancer (113), TM4SF1 in bladder cancer (108), CHD3 in platinum-resistant ovarian cancer (114), and SESN2 in proteasome-inhibitor-resistant multiple myeloma (111). YBX3 broadens the model beyond YBX1: in EBV-driven NPC, it recognizes NSUN2-modified ICAM-1 and recruits PABPC1 to enhance translation (64). ALYREF operates both alongside YBX1 and independently; in NSCLC, NSUN2 installs m5C on YAP mRNA; ALYREF engages the marked transcript first, and YBX1 is subsequently recruited to the ALYREF-bound complex, where its association is reported to impede AGO2 loading (63) Whether YBX1 actively displaces pre-bound AGO2 or occludes its recruitment is not resolved. In colorectal cancer, ALYREF stabilizes the lncRNA CASC19 to support a stemness program (115). The axis is best described as a small cooperative module whose composition can shift between tumors, rather than a fixed pair.

Across these examples, marked transcripts converge on a few fates: enhanced mRNA stability (31, 41, 65, 67, 108, 111, 113, 114), increased translation (63, 64), and exosomally delivered transcript protection (68). These outputs in turn feed proliferation and metastatic signaling (63–65, 67), immune-checkpoint maintenance (41, 62, 68), metabolic and migratory rewiring (108, 109, 115), homologous-recombination repair (114), and resistance to platinum, proteasome inhibitors, EGFR-TKIs, and 5-fluorouracil-associated autophagy (27, 66, 111, 113, 114). Selective preservation, not a shared downstream output, is what unifies the literature.

The strength of evidence is uneven. Mechanistic studies that include direct m5C mapping, RIP or MeRIP profiling, stability assays, mutagenesis, and rescue experiments (62, 64–68) are stronger than cohort-level signature analyses (110) or correlational reader-expression studies (109), which support clinical relevance without establishing direct causality. Tumor-type specificity is genuine, mechanistic depth is uneven, and many axes lack clinical validation; m5C marks likely cooperate with other RBPs and RNA modifications *in vivo*, and there is no evidence that m5C deposition universally favors cancer progression; its effects depend on which transcripts are marked and which readers are available. The m5C writer–reader axis therefore explains one major rule of YBX1 transcript selection, but not its full context specificity. The next subsection examines how non-coding RNAs, including circRNAs, lncRNAs, small RNAs, and damage-associated RNA fragments, provide a second layer of tuning that determines which YBX1 state and which targets dominate in a given cellular context.

### 5.3 Non-coding RNAs as regulators of YBX1 abundance and activity

Where the previous subsection examined how m5C marks select the RNAs YBX1 acts on, this subsection turns to the reverse direction: ncRNAs that act on the reader itself, modulating YBX1 abundance, activity, or functional availability rather than serving as targets.

Three circRNAs converge on a shared logic: protection of YBX1 from degradation. In breast cancer, hsa_circ_0007990 binds YBX1 and blocks its ubiquitin/proteasome-dependent turnover, increasing E2F1-driven proliferation (116). In hepatocellular carcinoma, circRNA-SORE binds cytoplasmic YBX1 and prevents PRP19-mediated ubiquitination, and is transferred via exosomes to spread sorafenib resistance (117); circZNF79(5) recruits the K63 deubiquitinase BRCC36, shielding YBX1 from p62-mediated selective autophagic degradation through AMPK/mTOR signaling (61). These reports support the possibility that circRNAs amplify oncogenic YBX1 outputs by extending its half-life.

Other ncRNA classes broaden this picture. The piRNA piR-YBX1 binds YBX1 mRNA directly and downregulates both transcript and protein levels in triple-negative breast cancer, suppressing MAPK signaling and tumor growth (118), an important counterexample showing that ncRNA–YBX1 interactions are not uniformly oncogenic. tRF-3017b is associated with prostate cancer progression and STAT3-linked immune modulation (119); the available evidence emphasizes its proliferative and immunomodulatory effects rather than a direct YBX1 regulatory mechanism.

Beyond chronic regulation, YBX1 is also engaged by damage- and infection-associated RNAs. Y3-derived ysRNAs are bound by YBX1 as an m5C reader; the complex localizes at DNA double-strand breaks and modulates PARP1 residency via ADP-ribosylation, with radioprotective effects transmitted by exosomes (120). In a separate setting, YBX1 is identified as an essential host RNA-binding protein for oncolytic virus M1 replication, binding viral 3′ UTR and the NSP4–capsid junction; here, YBX1 expression supports oncolytic efficacy and may serve as a predictive biomarker rather than a target to inhibit (121). Together, these mechanisms are heterogeneous, tumor-type specific, and unevenly validated *in vivo*; they help explain how YBX1 activity becomes context-specific nd prepare the scaffold mechanisms developed in Section 5.5, in which ncRNAs act not on YBX1 itself but as scaffolds directing YBX1 toward specific RNA targets.

### 5.4 YBX1 across the epitranscriptome: reading, scaffolding, and the limits of mark-specificity

The selection rules developed above are dominated by one mark, and this is not an artefact of emphasis but a feature of the evidence. It is worth stating explicitly how YBX1 relates to each of the major mRNA modifications, because the differences are informative for the hub model: YBX1 is not a general epitranscriptomic reader but a mark-specific one, and its involvement with modifications other than m5C is of a different and weaker kind. For 5-methylcytosine, as qualified in Section 5.2, YBX1 is best regarded as a functional m5C-dependent effector across the sixteen reader–target relationships compiled in **Table 2A**, fourteen of which involve a defined transcript. The writers are NSUN-family enzymes, principally NSUN2 with NSUN5 (122), NSUN6 (123) and DNMT2 (124) acting in restricted contexts outside the circuits tabulated in **Table 2A** (see Section 5.7). This is the axis on which the transcript-selection logic of Layer 2 rests, and, as noted in Section 5.2, its designation as a reader relationship rests on functional co-enrichment and W65-dependent binding rather than on a resolved structural mechanism.

For N6-methyladenosine, YBX1’s involvement is real but indirect, and of a categorically different kind. Rather than reading m6A itself, YBX1 participates in mixed epitranscriptomic scaffolds in which an m6A mark is deposited on a partner transcript. In colorectal cancer, LINC02418 is m6A-modified by METTL3 and interacts simultaneously with YBX1, which it directs to the CTNNB1 promoter, and with the canonical m6A reader IGF2BP1, which stabilizes CTNNB1 mRNA (69); the m6A mark is read by IGF2BP1, not by YBX1. Similarly, METTL3-dependent m6A upregulation of the lncRNA TUG1 engages YBX1 to activate PD-L1 and CD47 transcription (125), with the mark again residing on the scaffold rather than on a YBX1 target. YBX1 is therefore an m6A-adjacent factor, recruited within complexes that other proteins nucleate through m6A, rather than an m6A reader in its own right.

For N4-acetylcytidine, the single available link is of the same scaffold type. In breast cancer, the lncRNA CD2BP2-DT is stabilized by NAT10-mediated ac4C modification and, once stabilized, promotes YBX1 liquid–liquid phase separation to stabilize CDK1 mRNA and drive proliferation (126). As with m6A, the ac4C mark is deposited on the scaffold, not on a YBX1 target, and YBX1 is recruited to a phase-separated assembly rather than reading the modification directly. This is nonetheless a mechanistically informative circuit, because it connects an ac4C-stabilized lncRNA to the same phase-separation behavior of YBX1 that arginine methylation controls in Layer 1 (Section 4.1), suggesting that phase separation may be a common downstream node through which structurally unrelated inputs converge on YBX1 function.

For pseudouridine, no reader relationship with YBX1 has been demonstrated, and the connection that exists is at the level of crosstalk between modifications rather than direct recognition. Pseudouridine synthases, principally PUS7, are increasingly implicated in cancer (127–129). Pseudouridine synthase depletion has been reported to reshape the global m5C and m6A modification landscape, suggesting regulatory crosstalk between RNA modification systems (130). This situates pseudouridylation upstream of the m5C system that YBX1 reads, but provides no evidence that YBX1 recognizes pseudouridine itself. We therefore treat any YBX1–pseudouridine relationship as unestablished rather than merely under-studied.

Read together, these distinctions sharpen rather than broaden the hub model. YBX1’s epitranscriptomic function is mark-specific: it reads m5C, is recruited into m6A- and ac4C-nucleated scaffolds without reading those marks, and has no demonstrated relationship with pseudouridine. This specificity is itself a prediction of the framework, since a promiscuous, abundant RNA-binding protein would be expected to associate indiscriminately with modified transcripts of every class, whereas a state-dependent reader should show exactly the graded, mark-specific pattern observed. The framework would be weakened, not supported, by evidence that YBX1 reads every modification equally; that no such evidence exists is consistent with the selective-reader account developed here.

### 5.5 Non-coding RNA scaffolds: directing YBX1 to target transcripts

Where Section 5.3 examined ncRNAs that set YBX1’s abundance, this one turns to ncRNAs that influence where YBX1 acts, bringing it into proximity with specific transcripts, partners, or transcriptional programs rather than altering its half-life. CircRNAs illustrate this scaffold logic. In glioma, circEPHB4 binds YBX1 and enhances YBX1-mediated m5C modification of MRPS16 mRNA, while also competing with RBBP6 to limit YBX1 ubiquitination (70), a dual mechanism that bridges scaffold-style guidance and abundance control. In hepatocellular carcinoma, super-enhancer-driven circPVT1 recruits YBX1 to the nucleus, where it transcriptionally activates RRM2 to support growth and metastasis (71). CircPOLD1 in cervical lesions is associated with YBX1 and an AKT/mTOR/HIF-1α glycolytic program; here the evidence is strongest as a stage-specific biomarker (AUC 0.951 for LSIL+ detection), and the mechanism warrants further validation (72).

LncRNAs provide a parallel set of scaffolds. Under hypoxia, USP2-AS1 enhances YBX1-mediated HIF1-α protein translation in HCC and modulates sensitivity to lenvatinib (73). In glioblastoma stem-like cells, DARS1-AS1 interacts with YBX1 to stabilize target mRNAs including E2F1, CCND1, and FOXM1, sustaining G1–S progression, homologous-recombination repair, and radioresistance (74). In breast cancer, SBF2-AS1 binds YBX1 in a reciprocal positive feedback loop that engages PI3K/AKT/mTOR signaling and reduces tamoxifen sensitivity (75).

LINC02418 in colorectal cancer adds a further nuance: it is itself m6A-modified by METTL3, distinct from the m5C-on-mRNA reading discussed in Section 5.2, and interacts simultaneously with YBX1 (enhancing YBX1 binding to the CTNNB1 promoter) and IGF2BP1 (stabilizing CTNNB1 mRNA) (69). This is consistent with YBX1 participating in mixed epitranscriptomic scaffolds alongside other RNA-binding proteins.

Mechanistically, most reports include direct interaction and rescue evidence (69–71, 73–75), whereas circPOLD1 rests chiefly on biomarker-level data (72). These scaffolds differ by tumor type, RNA class, and assay depth, supporting a context-specific scaffold model rather than a unified rule. Together with m5C reading (Section 5.2) and ncRNA-mediated YBX1 stabilization (Section 5.3), scaffold ncRNAs help explain how YBX1-dependent RNA programs become tumor-context specific and generate the immune, metabolic, and therapy-resistance outputs examined in Layer 3.

### 5.6 From selective transcripts to survival modules (bridge to Layer 3)

The three selection rules examined so far, m5C writer–reader recognition (Section 5.2), ncRNA-mediated control of YBX1 abundance and activity (Section 5.3), and ncRNA scaffolds that direct YBX1 to specific targets (Section 5.5), are mechanistically distinct, but the transcripts they preserve are not. Read across the studies cited in this section, the YBX1-selected transcriptome collapses into a small number of survival modules whose outputs Layer 3 examines in detail.

The first is an immune-evasion module, in which YBX1 supports checkpoint expression and microenvironmental reshaping through transcripts including PD-L1, STAT1, PHGDH, and CCL2-coupled programs (31, 41, 62–64, 68, 72, 111). The second is a metabolic and migratory module, encompassing glycolytic and lipogenic rewiring, hypoxia adaptation, and migratory signaling through HIF1-α, TM4SF1, SCD, RRM2, and CTNNB1-linked outputs (69, 71, 73, 108, 109, 115, 116). The third is a genome-integrity and autophagy module, in which m5C-marked or scaffolded transcripts sustain homologous-recombination repair, selective autophagy, and damage-associated PARP1 activity (27, 61, 74, 114, 120). The fourth is a therapy-resistance module that integrates the previous three: stabilization of survival transcripts, scaffold-driven kinase circuits, and protection of YBX1 itself contribute to resistance against platinum, EGFR-TKIs, sorafenib, lenvatinib, 5-fluorouracil, proteasome inhibitors, and endocrine therapy (66, 73, 75, 111, 113, 114, 117).

These modules are not mutually exclusive; most tumors engage more than one — and the boundaries between them are softer than the headings suggest. What unifies them is selective preservation, not a shared downstream output. Sections 6–8 examine these modules in turn: the immune-ecological outputs that follow most directly from the RNA program described here (Section 6), the metabolic outputs with which they are coupled (Section 7), and the plasticity and resistance phenotypes that integrate both (Section 8). These four modules are not orthogonal categories but operational groupings: they reflect the four most consistently reported phenotypic outputs of the YBX1-selected transcriptome in this evidence base, while acknowledging that metabolic and immune programs can be coupled at the molecular level (for example, through lactate–lactylation feedback) and will be examined together where the underlying mechanism warrants.

### 5.7 Convergent architecture: what is shared across the layers

The three layers are presented sequentially, but the circuits within them are not independent, and four features recur across the evidence base in a way that a layer-by-layer reading obscures. The first is a shared funnel: mechanistically unrelated inputs converge on a single event, the appearance of YBX1 in the nucleus. The first input class is phosphorylation, which reaches this outcome through at least five kinase routes and three distinct residue sets, S102 by AKT, p70S6K1 and p90RSK (46, 47), S174 and S176 by PLK1 (48); S165 through RIOK1 (49), and further sites set by MASTL (131), MARK2 (40) and S6K1 (77, 132). The second is metabolite-driven covalent modification: O-GlcNAcylation at T57 (36); and arginine methylation that suppresses cytoplasmic phase separation as a prerequisite for nuclear entry (56). The third is the import machinery itself, engaged either directly through karyopherin-mediated transport (107); or indirectly through partner and scaffold proteins, Menin (76), paxillin (104) and circIPO7 (47), that deliver YBX1 to it. The counter-regulatory circuits are informative here precisely because they invert the same node: CXCL2 (133), ST7 (134), FLII (43) and CDKL1 (135) restrain nuclear YBX1 or its promoter occupancy, and each is associated with a tumor-suppressive rather than tumor-promoting phenotype. Nuclear access is therefore not one regulatory step among many but the point at which Layer 1 commits.

The second is convergence on a small number of AGC-family kinases that phosphorylate YBX1 at S102. These are reached from two distinct upstream cascades, PI3K/AKT/mTORC1 acting through p70S6K1, and RAS–RAF–MEK–ERK acting through p90RSK, which converge on the same modification. PI3K/AKT-linked circuits recur most frequently, appearing in every Layer 3 module, from ubiquitin-dependent turnover (58, 59) and deSUMOylation-coupled AKT activation (57) through antiestrogen (46, 75) and lenvatinib (49) resistance to immune-checkpoint induction (77) and cholesterol-mediated sorafenib tolerance (136). The dual routing to a single site is itself informative: it predicts that single-arm upstream inhibition will be escaped by compensatory activation of the other arm, as observed when RSK inhibition activates AKT in KRAS-mutant colorectal cancer (137), and it argues that state-directed intervention should be aimed at the modification or at nuclear access rather than at one upstream kinase (Section 10.3) (**Table 3**).

**Table 3:** Major signalling pathways engaged by YBX1: mechanism of engagement, representative circuits, and direction of effect.

Pathway	Mechanism of YBX1 engagement	Representative circuit (tumor type)	Direction of effect	Evidence	Ref.
3-A. Core proliferative and survival signaling					
PI3K/AKT/mTORC1	circIPO7 scaffolds cytoplasmic YBX1 for AKT-mediated S102 phosphorylation → nuclear entry	Nasopharyngeal carcinoma	↑ FGFR1/TNC/NTRK1 transcription; metastasis, cisplatin resistance	M	(47)
PI3K/AKT/mTORC1	S102 phosphorylation by AKT, p70S6K1 and p90RSK; suppressed by TAS0612 + everolimus	Breast (antiestrogen-resistant; TNBC)	↑ drug-resistance gene program; fulvestrant and tamoxifen resistance	M	(46)
PI3K/AKT/mTORC1	SENP1-mediated deSUMOylation at K26 preserves the YBX1–DDX5 interaction	Colorectal	↑ AKT phosphorylation; proliferation, migration; poor outcome	M	(57)
PI3K/AKT/mTORC1 ¶	TRIM29-mediated ubiquitination and proteasomal degradation of YBX1	Hepatocellular carcinoma	↓ PI3K/AKT; restores lenvatinib sensitivity; TRIM29-high = longer OS	M	(58)
PI3K/AKT/mTORC1 ¶	FBXW11 K48-linked polyubiquitination at the cold-shock domain → degradation	Hepatocellular carcinoma	↓ AKT/mTOR; suppresses proliferation and xenograft growth	M	(59)
PI3K/AKT/mTORC1	YBX1–SBF2-AS1 reciprocal positive feedback loop	Breast	↑ proliferation; reduced tamoxifen sensitivity	M (*in vitro* only)	(75)
PI3K/AKT/mTORC1	IRGM promotes S6K1-mediated phosphorylation and nuclear translocation	Hepatocellular carcinoma	↑ PD-L1 transcription; ↓ CD8+ CTL infiltration; anti-PD-1 synergy on inhibition	M	(77)
RAS–RAF–MEK–ERK (p90RSK)	EPS8L2 enhances the S6K1–YBX1 interaction → nuclear YBX1 → G3BP2 transactivation	Colorectal	↑ MAPK signalling; proliferation, migration	M	(132)
RAS–RAF–MEK–ERK (p90RSK) ‡	Oncogenic KRAS drives RSK-mediated YB-1 phosphorylation; RSK inhibition activates compensatory AKT	Colorectal (KRAS-mutant)	Cetuximab resistance; dual RSK + AKT blockade sensitizes to 5-FU	M	(137)
3-B. Inflammatory and immune signaling					
NF-κB	TRIM31-catalysed K63-linked polyubiquitination at K81 stabilizes YBX1; P65 transactivates *TRIM31* (feedback)	Colorectal	↑ EREG/GAS6/MAFG transcript stability; inflammation-linked carcinogenesis	M	(60)
NF-κB	S100A8/A9–BFAR degrades PRP19 → YBX1 stabilization → CXCL1/CXCL3 transactivation; neutrophil-derived S100A8/A9 re-induces BFAR (feedback)	Gastric	↑ neutrophil infiltration, T-cell exhaustion; poor anti-PD-1 response	M	(140)
NF-κB	RECQL4 acts as a scaffold enhancing the YBX1–G3BP1 interaction	Lung adenocarcinoma	↑ proliferation, migration, invasion	M	(145)
NF-κB	KIF14 binds the G3BP1/YBX1 complex and facilitates their interaction	Cholangiocarcinoma	↑ NF-κB promoter activity; lymphatic metastasis, gemcitabine resistance	M	(146)
JAK2/STAT3	RIOK1 induces Ser165 phosphorylation → nuclear localization	Hepatocellular carcinoma	↑ JAK2/STAT3 phosphorylation; lenvatinib resistance	M	(49)
JAK2/STAT3	PXN stabilizes YB-1 by inhibiting its ubiquitination, within a STAT3–PXN–SRC feedback loop	Glioblastoma, IDH-wildtype	↑ malignancy; poor prognosis	M	(104)
STAT1/interferon	YBX1 reads m5C on *STAT1* mRNA, sustaining its stability and translation	Intrahepatic cholangiocarcinoma	↑ STAT1/PD-L1 axis; suppressed antitumor T-cell responses	M	(41)
3-C. Developmental and growth-control signalling					
Wnt/β-catenin	LINC00665–YBX1 feed-forward loop; YBX1 regulates *Wnt3a* transcriptional activity	Gastric	↑ EMT, proliferation, pulmonary metastasis	M	(139)
TGF-β	RRM2 stabilizes YBX1, which upregulates TGFBR1	Pancreatic	↑ TGF-β pathway activation; liver metastasis	M	(144)
Hippo/YAP	NSUN2-deposited m5C on *YAP* mRNA bound first by ALYREF, then by YBX1, impeding AGO2; YAP–TEAD2 transactivates *NSUN2* (feedback)	Non-small-cell lung cancer	↑ YAP stability and translation; growth, EMT, invasion, metastasis	M	(63)
3-D. Metabolic and redox signaling					
HIF-1α/hypoxia	TROP2 drives lactate production through a YBX1–HIF-1α arm; H3K18la sustains *TROP2* (feedback)	Colorectal (liver metastasis)	↑ glycolysis, metastatic colonization; suppressed by acriflavine	M	(84)
Ferroptosis/system x_c_^−^	ODC1 binds YBX1 via its PLPDE_III_ODC domain; the complex occupies the *SLC7A11* promoter	Gastric adenocarcinoma	↑ SLC7A11; ↓ lipid peroxidation and glutathione depletion; 5-FU resistance reversed by erastin	M	(142)
Ferroptosis/system x_c_^−^ ‖	YBX3 normally promotes SLC7A11 degradation; USP5 redirects YBX3 to lysosomal degradation	Colorectal	Direction inverted at family level — YBX3 loss stabilizes SLC7A11 and suppresses ferroptosis	M	(143)
Lactate–lactylation feedback	Lactate induces YBX1 nuclear translocation; YBX1 transactivates and stabilizes *LDHB* mRNA	Intrahepatic cholangiocarcinoma	Lactate → pyruvate → TCA flux and ATP; proliferation	M	(82)
Lactate–lactylation feedback	H3K18 lactylation enriches at the *YBX1* (and *YY1*) promoter, driving their transcription	Bladder	Cisplatin resistance in a glycolytic cell subpopulation	M (single-cell)	(83)

Pathways engaged by YBX1, grouped into four thematic bands and, within each band, ordered by the number of independent circuits documented. For each entry the table states the molecular mechanism by which YBX1 is coupled to the pathway, the tumor context in which it was demonstrated, and the direction of the resulting effect. Evidence classification: M, mechanistic evidence supported by direct molecular interaction and functional perturbation with rescue and/or *in vivo* validation. ¶ denotes a counter-regulatory circuit, one in which YBX1 activity opposes the malignant/immune-evasive phenotype, or in which restraining YBX1 is protective; retained deliberately as evidence against a universal oncogenic role. ‡ denotes compensatory signaling, specifically compensatory AKT activation following RSK inhibition in the KRAS-mutant colorectal cancer circuit.

The third is a dominant writer. Although several enzymes deposit or shape the marks that YBX1 reads, NSUN2 accounts for the majority of the m5C circuits assembled here (27, 62–68), with NSUN5 (122), NSUN6 (123) and DNMT2 (124) acting in restricted contexts. The selection layer, like the state layer, has a hub of its own, which has implications for whether the writer or the reader is the more tractable target.

The fourth is architectural rather than molecular. Positive feedback recurs across otherwise unrelated circuits: lactate and H3K18 lactylation returning to YBX1 (36, 82–84); uridine-derived K137 lactylation stabilizing the protein (53); and reciprocal loops with STAT3 and paxillin (104), PSAT1 (40), MYC (138), SBF2-AS1 (75), LINC00665 (139), NF-κB/P65 (60), YAP (63) and neutrophil-derived NF-κB signalling (140). The recurrence of feedback across such heterogeneous settings suggests that recursion is a property of the hub rather than a feature of any single tumor context (Section 2).

One further convergence is worth noting because it cuts across the modality distinction drawn in Section 5.1. Several effectors are reached by more than one route: PD-L1 is induced transcriptionally through promoter occupancy (43, 77–79, 125, 135) and stabilized post-transcriptionally as an mRNA (41, 68, 141); SLC7A11 is regulated through YBX1 in gastric cancer (142) and through YBX3 in colorectal cancer (143); RRM2 is activated by acetylated YBX1 (54) and by scaffold-recruited YBX1 (71), and also stabilizes YBX1 in return (144). The same phenotypic endpoint is therefore reachable from different states, which is the practical reason why abundance-based measurement fails and why the stratification logic of Section 10.1 is state- rather than level-based.

Two partners deserve mention as connective tissue. G3BP1 and G3BP2 recur across circuits that are otherwise assigned to different modules, stress-granule formation (30), lactylation-dependent binding (51), NF-κB activation through RECQL4 (145) and KIF14 (146), and MAPK signaling through EPS8L2 (132), suggesting that the stress-granule literature and the kinase-signaling literature describe overlapping rather than separate biology.

## 6 Layer 3a: immune reprogramming: from PD-L1 RNA stability to immune-ecological remodeling

### 6.1 Interferon signaling, antigen presentation, and innate immune sensing

Checkpoint expression is the most frequently reported immune output of YBX1, but it is downstream, and several of the circuits assembled here engage interferon and innate-sensing pathways upstream of it. Read in that order, the evidence suggests that YBX1 acts on the machinery that determines whether a tumor is seen, not only on the ligands that determine whether it is attacked. The clearest interferon-proximal circuit operates at STAT1. In intrahepatic cholangiocarcinoma, YBX1 acts as an m5C reader that sustains STAT1 stability and translation, and PD-L1 expression follows as a consequence of preserved STAT1 output rather than through direct promoter engagement (41). Because STAT1 is an obligatory component of both the IFN-γ-activated GAF homodimer and the type I interferon ISGF3 complex (STAT1–STAT2–IRF9), this places YBX1 within the interferon response itself: a tumor with high reader activity does not silence interferon signaling but redirects its output toward checkpoint ligand expression. A second circuit runs in the opposite direction, with interferon acting as the input. In hepatocellular carcinoma, IFN-γ induces IRGM, which promotes S6K1-mediated phosphorylation and nuclear translocation of YBX1 and thereby PD-L1 transcription, with CD8+ cytotoxic T-lymphocyte infiltration falling as a result (77). Taken together, these two studies describe a feedback relationship in which interferon exposure sets the YBX1 state and YBX1 in turn shapes what the interferon response produces, although neither study was designed to test that relationship directly and the inference should be treated as a hypothesis.

Innate sensing is engaged through the DNA-damage axis. In gastric cancer, TAGLN2-driven AKT–YBX1 signaling promotes accumulation of single-stranded DNA and is accompanied by cGAS–STING activation and an interferon-stimulated gene signature (147). Because cGAS is a double-stranded-DNA sensor, ssDNA accumulation is unlikely to be the direct activating ligand; the intervening step, whether cytosolic dsDNA species, micronuclei, or an indirect route, is not resolved in that study, and the ISG induction should be read as correlated with, rather than mechanistically attributed to, ssDNA. Related evidence comes from viral contexts, where NSUN2 induction downstream of EBV LMP1 and NF-κB places the m5C writer–reader axis under the control of an innate signaling cascade (64), and where YBX1 is required as a host factor for oncolytic virus M1 replication, making its expression a candidate predictor of oncolytic efficacy rather than a target for inhibition (121).

Antigen presentation is the least developed of these axes and the most consequential. The strongest evidence is family-level: in glioma, CHK2, YBX1 and YBX3 form a reciprocally reinforcing hub whose pharmacological disruption derepresses pro-inflammatory gene expression, enhances antigen presentation, expands antigen-specific CD8+ T cells, and synergizes with checkpoint blockade in preclinical models (148). A complementary mechanism operates at the non-classical presentation molecule HLA-E, where PSAT1-driven MARK2-dependent phosphorylation promotes nuclear YBX1, which transactivates HLA-E and suppresses NK-cell killing (40). These two studies indicate that YBX1-dependent circuits can restrict both classical and non-classical presentation, but they remain the only direct evidence in this evidence base, and neither addresses the antigen-processing machinery itself. Whether YBX1 influences the expression of MHC class I components, the peptide-loading complex, or the immunoproteasome is, to our knowledge, untested, and we regard this as the single largest gap in the immunological account of YBX1.

A further gap should be stated plainly. We identified no study addressing YBX1 function in dendritic cells, either tumor-intrinsically through effects on antigen availability or cell-intrinsically within the dendritic-cell compartment. Given that YBX1 has documented functions in other immune lineages, including translational control of immunoglobulin transcripts in B cells (149), the absence of dendritic-cell data reflects the questions the field has asked rather than an established negative, and it is an obvious priority for work aimed at coupling YBX1 inhibition to checkpoint blockade.

### 6.2 PD-L1 and checkpoint control as RNA-stability phenotypes

PD-L1 provides a recognizable entry point into the immune layer of YBX1 biology, but the available evidence increasingly recasts PD-L1 expression in YBX1-active tumors not as a discrete checkpoint switch but as one phenotypic output of YBX1-dependent RNA-stability, localization, and stress-adaptive regulation; the immune-reprogramming circuits are mapped by tumor type, dominant circuit, phenotypic output, and therapeutic node in the immune module of **Table 4**. At the transcriptional level, YBX1 has been reported to bind a PD-L1 promoter motif directly and to drive PD-L1 induction under chemotherapeutic pressure in hepatocellular carcinoma; in this setting, YBX1 knockdown reverses chemoresistance by restoring effector CD8+ T-cell activity and reducing Treg and MDSC infiltration, illustrating that immune evasion and multidrug resistance can be coupled outputs of the same circuit (78). This promoter-level regulation can be gated upstream by phosphorylation and localization: IRGM has been shown to promote S6K1-mediated phosphorylation and nuclear translocation of YBX1, increasing PD-L1 transcription and CD8+ CTL suppression in HCC, an axis whose disruption appears to potentiate anti-PD-1 therapy (77). A complementary scenario emerges in prostate cancer, where FLII normally restrains YBX1 nuclear localization and PD-L1 transcription; FLII loss accompanying enzalutamide resistance activates the YBX1/PD-L1 axis, expands Tregs and MDSCs, and suppresses effector CD8+ T-cell responses (43). Negative-regulator evidence from lung cancer reinforces this picture: CDKL1 binds YBX1 and reduces its occupancy at the PD-L1 promoter, lowering PD-L1, activating CD8+ T-cells, and enhancing radioimmunotherapy (135).

**Table 4:** Layer 3a: tumor-by-circuit map of YBX1 dependency, immune module.

Tumor type	Dominant module	Key YBX1 circuit	Phenotypic output	Therapeutic node & agent	Evidence	Ref.
4-A. PD-L1 and checkpoint control as RNA-stability phenotypes						
Breast cancer (TNBC)	Immune (PD-L1 glycosylation)	YY1 → RPN1 → YBX1 (PD-L1 regulatory axis); RPN1 glycosylates/stabilizes PD-L1	PD-L1 stability → immune escape; growth/metastasis	RPN1 targeting + anti-PD-1	M (YBX1’s specific role within the YY1/RPN1 axis not resolved)	(150)
Diffuse large B-cell lymphoma	Immune (PD-L1 mRNA stability)	Exosomal NSUN2 → m5C → YBX1 → PD-L1 mRNA stabilization	Immune escape, M2 macrophage polarization	NSUN2–PD-L1 axis targeting	M (reader–target detail, **Table 2A**)	(68)
Hepatocellular carcinoma	Immune (PD-L1 transcription)	YBX1 → PD-L1 promoter (chemotherapy-induced)	PD-L1 ↑, immune evasion + multidrug resistance; ↓ CD8, ↑ MDSC/Treg	YBX1 knockdown (restores T-cell activation)	M	(78)
Hepatocellular carcinoma	Immune (PD-L1 transcription)	IFN-γ → IRGM → YBX1–S6K1 → YBX1 phosphorylation/nuclear entry → PD-L1	↓ CD8 CTL infiltration; immune evasion	IRGM inhibition + anti-PD-1	M (scRNA-seq)	(77)
Intrahepatic cholangiocarcinoma	Immune (PD-L1)	YBX1 (m5C reader) → STAT1 stability/translation → STAT1/PD-L1	Suppressed antitumor T-cell responses	YBX1 as immunotherapy target (no specific agent)	M (reader–target detail, **Table 2A**)	(41)
Lung cancer	Immune (PD-L1 transcription)	CDKL1 binds YBX1 → ↓ YBX1 affinity for PD-L1 promoter → ↓ PD-L1	CD8 activation; ↓ immune evasion; radiosensitization	CDKL1 restoration + radiotherapy + anti-PD-L1	M	(135)
Multiple solid tumors	Immune (PD-L1 mRNA stability)	MNX1 enhances YBX1 binding to PD-L1 mRNA (stabilization, not transcription)	PD-L1 ↑ → cytotoxic-T-cell evasion	MNX1 ablation — sensitizes CTLA-4 blockade	M	(141)
Prostate cancer	Immune (PD-L1 transcription)	Endocrine resistance → FLII ↓ → YBX1 nuclear entry → PD-L1	Immune evasion + enzalutamide resistance; FLII restoration → ↑ CD8, ↓ Treg/MDSC	FLII restoration + endocrine therapy	M	(43)
4-B. Myeloid reprogramming: MDSCs, macrophages, neutrophils						
Breast cancer (luminal)	Immune (TME correlation)	YBX1 expression ~ M2 macrophages, IDO1/CTLA4	M2 infiltration, T-cell exhaustion markers; poor prognosis	ICI + M1-polarization agents (proposed)	A (TIMER/database + co-culture)	(154)
Gastric cancer	Immune (neutrophil)	S100A8/A9–BFAR → K48-Ub degradation of PRP19 → YBX1 stabilization → CXCL1/CXCL3	Neutrophil infiltration, immunosuppressive reprogramming, T-cell exhaustion; poor anti-PD-1 response	BFAR inhibition + ICB	M	(140)
Hepatocellular carcinoma	Immune (neutrophil) ¶	Intracellular CXCL2 binds YBX1 → blocks YBX1 nuclear entry → ↓ SREBF2/cholesterol	Antitumor neutrophil polarization; CXCL2-low tumors are immune-cold	CXCL2 restoration (therapeutic target)	M (counter-regulatory: YBX1 restrained, not activated) ¶	(133)
Hepatocellular carcinoma	Immune (PD-L1/CD47)	METTL3-m6A → TUG1 → YBX1 → PD-L1 and CD47 transcription	↓ CD8 activation, ↓ macrophage phagocytosis; immune evasion	TUG1-siRNA + anti-PD-L1	M	(125)
Hepatocellular carcinoma	Immune (TAM signature)	YBX1 = 1 of 7 genes in an SPP1+ macrophage prognostic signature	Prognostic risk stratification (SPP1+ TAM context)	None (prognostic tool)	A (signature-level; no YBX1-specific mechanism)	(155)
Lung adenocarcinoma	Immune (IL6/CCL5) + metastasis	YBX1 → IL6 and CCL5; OGT-T271 glycosylation–mitophagy controls YBX1 stability	Immunosuppressive microenvironment + bone metastasis	Icaritin (potentiates YBX1 glyco-degradation) — ↓ bone metastasis, sensitizes immunotherapy	P (conference abstract)	(152)
Pancreatic ductal adenocarcinoma	Immune (MDSC)	TMBIM1 → YBX1 → ↑ affinity for PD-L1 and CCL2 promoters	MDSC infiltration; immunosuppressive microenvironment	TMBIM1 knockdown + anti-PD-1	M (scRNA-seq)	(79)
4-C. Lymphocyte, NK-cell, and immune-effector circuits						
Lung adenocarcinoma	Immune (CCL5/CD8 recruitment) ¶	MIR155HG → YBX1 stabilization (↓ ubiquitination) → CCL5; MIR155HG also ↑ PD-L1	Pro-immune — ↑ CD8 infiltration; high MIR155HG ~ better survival and immunotherapy response	MIR155HG-high + anti-PD-L1 (response biomarker)	M (context-dependent: this YBX1 axis is pro-immune) ¶	(151)
Prostate cancer	Immune (NK/HLA-E)	PSAT1 → MARK2 → YBX1 phosphorylation/nuclear entry → HLA-E (PSAT1 feedback loop)	NK-cell inactivation (immune-cold phenotype)	PSAT1 targeting + NK cytotherapy	M	(40)
4-D. YBX-family circuits and immunotherapy response						
Clear cell renal cell carcinoma	Immune + epigenetic (m5C)	NSUN5/circPPAP2A → YBX1 nuclear entry and phosphorylation → HIF-2α; ↓ CCL5, ↑ TGFB1	↓ CD8 infiltration, T-cell exhaustion; co-resistance to HIF-2α inhibitor + anti-PD-1	NSUN5/circPPAP2A knockdown (nanoparticle siRNA) + anti-PD-1	M (scRNA-seq + spatial + *in vivo*)	(122)
Glioblastoma/glioma	Immune (antigen presentation) ‖	CHK2–YBX1–YBX3 reciprocal hub (cooperative, family-level) ‖	Repressed pro-inflammatory genes; CD8 resistance; hub depletion → antigen presentation and CD8 expansion	YBX1 inhibitor (degrades the CHK2–YBX1/YBX3 hub) + ICB	M (YBX1 and YBX3 cooperative) ‖	(148)

Rows are grouped into four thematic bands corresponding to Sections 6.2, 6.4, 6.5 and 6.6 and, within each band, ordered alphabetically by tumor type. Evidence classification: M, mechanistic evidence supported by direct molecular interaction and functional perturbation with rescue and/or *in vivo* validation; A, associative evidence based on correlation, signatures, or database-derived inference without demonstrated directionality; P, preliminary evidence including conference abstracts, limited models, or non-peer-reviewed observations. ¶ denotes a counter-regulatory circuit, one in which YBX1 activity opposes the malignant/immune-evasive phenotype, or in which restraining YBX1 is protective; retained deliberately as evidence against a universal oncogenic role.

A distinct mode operates at the RNA level. In multiple tumor contexts, YBX1 appears less as a transcription factor and more as a transcript-stabilizing reader: MNX1 interacts with cytoplasmic YBX1 and enhances its binding to PD-L1 mRNA, stabilizing the transcript and reducing cytotoxic T-cell sensitivity, MNX1 ablation was additionally reported to sensitise tumours to CTLA-4 blockade (141), an effect not obviously predicted by PD-L1 destabilisation alone and one whose mechanism is not resolved in that study. Exosomal NSUN2 from diffuse large B-cell lymphoma cells stabilizes PD-L1 mRNA in a YBX1-dependent, m5C-dependent manner, supporting M2 macrophage polarization alongside immune escape (68). In intrahepatic cholangiocarcinoma, the m5C-reading function of YBX1 is engaged one step upstream, enhancing STAT1 translation/stability and sustaining the STAT1/PD-L1 pathway (41). A separate triple-negative breast cancer study implicates a YY1/RPN1/YBX1 regulatory axis in PD-L1 regulation and immune escape, although the precise YBX1-resolved mechanism in that axis remains to be fully defined and should be interpreted cautiously (150).

**Figure 3:**
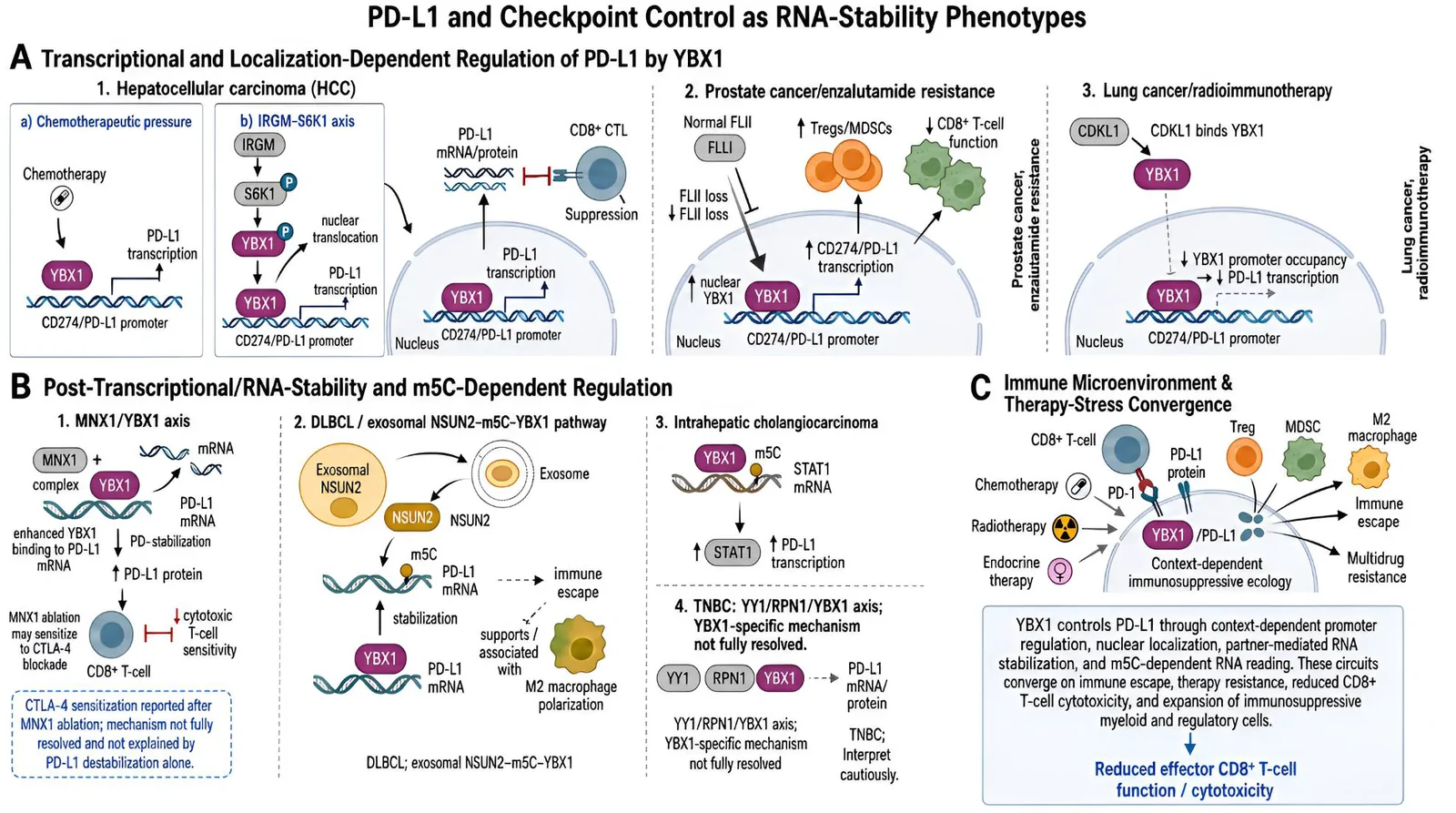
**(A)** YBX1 regulates PD-L1 through context-dependent transcriptional and localization mechanisms across different tumor types, including phosphorylation-driven nuclear translocation and promoter binding. **(B)** YBX1 controls PD-L1 expression at the post-transcriptional level via RNA stability programs, including MNX1-mediated mRNA stabilization, NSUN2/m5C-dependent regulation, and STAT1-linked pathways. **(C)** These integrated circuits converge on PD-L1–driven immune checkpoint activation, leading to immune evasion, therapy resistance, and establishment of an immunosuppressive tumor microenvironment.

Taken together, these observations are consistent with a model in which YBX1 can shape PD-L1 expression through promoter binding, phosphorylation-driven nuclear localization, partner-licensed mRNA stabilization, or m5C-mediated reading of intermediate transcripts, rather than implying a universal mechanism. Across these modes, the downstream ecology converges: chemotherapeutic, endocrine, and radiotherapeutic pressures appear to amplify YBX1-linked checkpoint output while simultaneously expanding Tregs, MDSCs, and M2 macrophages and constraining effector CD8+ T-cell function. YBX1-active tumors do not merely express immune checkpoints; they manufacture an immune-permissive ecology through RNA-stability, metabolic, and myeloid circuits (**Figure 3**).

### 6.3 YBX1 as a chemokine and cytokine regulator

The circuits described so far act on ligands displayed by the tumor cell. A second and largely separate body of evidence indicates that YBX1 also determines which soluble mediators the tumor releases, and it is through this output that the composition of the infiltrate is set. Read together, these studies describe YBX1 as a transcriptional regulator of a restricted chemokine repertoire rather than of cytokine output in general. The recruiting chemokines are the best represented. In pancreatic ductal adenocarcinoma, TMBIM1 increases YBX1 affinity for both the CCL2 and PD-L1 promoters, coupling monocytic recruitment to checkpoint expression within a single transcriptional program and driving MDSC infiltration (79). In gastric cancer, S100A8/A9–BFAR signaling stabilizes YBX1 through PRP19, and nuclear YBX1 transactivates CXCL1 and CXCL3 to recruit neutrophils, which then sustain BFAR through NF-κB in a self-reinforcing loop (140). In high-grade B-cell lymphoma, the NSUN2/YBX1-stabilized PHGDH transcript feeds both PD-L1 expression and CCL2-dependent macrophage recruitment (62). The direction is not uniform: in lung adenocarcinoma, MIR155HG-stabilized YBX1 drives CCL5 transcription and CD8+ T-cell recruitment, and high MIR155HG expression is associated with better survival and better immunotherapy response (151), while in clear cell renal cell carcinoma the NSUN5/circPPAP2A/YBX1 axis suppresses CCL5 and elevates TGFB1, reducing CD8+ infiltration and promoting exhaustion (122). The same chemokine is therefore induced in one setting and repressed in another, which is difficult to reconcile with a fixed transcriptional output and more consistent with the state-dependent model developed in Layers 1 and 2.

Interleukin and growth-factor outputs are less well covered but point in the same direction. In pancreatic cancer, GFPT2-driven O-GlcNAcylation places YBX1 in the nucleus where it acts as a transcription factor for IL-18, with downstream M2 macrophage polarization (52), an example in which a metabolic input is converted directly into a cytokine output. In bone-metastatic lung adenocarcinoma, YBX1 has been associated with IL6 and CCL5 signaling and an immunosuppressive niche, although this evidence remains at conference-abstract stage and should be weighted accordingly (152). A counter-regulatory case is provided by intracellular CXCL2, which binds YBX1 and blocks its nuclear entry, reducing SREBF2-driven cholesterol biosynthesis and favoring antitumor neutrophil polarization (133); here a chemokine acts on YBX1 rather than the reverse. Two observations follow. First, the chemokines under YBX1 control cluster tightly around myeloid recruitment (CCL2, CXCL1, CXCL3) and T-cell recruitment (CCL5), rather than spanning the cytokine repertoire, which is consistent with the transcript-selective logic of Layer 2 operating at the level of promoters as well as transcripts. Second, because several of these circuits are engaged by metabolic inputs- hexosamine flux at IL-18 (52), lactate at the H3K18la-coupled loops (84), arginine at NOS2 and ARG2 (153), the chemokine layer is where YBX1-dependent metabolism and YBX1-dependent immunity meet, and it is the most direct evidence available that the two are one program rather than two.

### 6.4 Myeloid reprogramming: MDSCs, macrophages, neutrophils, and immunosuppressive niches

The immune-modulatory reach of YBX1 extends well beyond PD-L1 expression on tumor cells: the available evidence supports a model in which YBX1 activity can be translated into stromal and myeloid niche construction through chemokine-driven recruitment, polarization, and metabolic suppression. A direct example is provided in pancreatic ductal adenocarcinoma, where TMBIM1 binds YBX1 and increases its affinity for both the CCL2 and PD-L1 promoters, simultaneously upregulating chemoattractant and checkpoint outputs to drive MDSC infiltration and reduce anti-PD-1 efficacy; here YBX1 appears to function as a coordinator that aligns recruitment and effector silencing into a single transcriptional program (79). A complementary metabolic mode operates in prostate cancer bone metastasis, where loss of the lncRNA SNHG18, which normally recruits TRIM21 to degrade YBX1, releases YBX1-mediated transcription of NOS2 and ARG2, depleting arginine, fostering infiltration of immunosuppressive cells, impairing effector T-cell function, and blunting anti-PD-1 responsiveness, illustrating how YBX1 can shape myeloid-skewed niches through nutrient depletion rather than chemokine output alone (153).

Neutrophil-centered reprogramming provides another distinct axis. In gastric cancer, the S100A8/A9–BFAR–PRP19–YBX1 cascade stabilizes YBX1, which transcriptionally upregulates CXCL1/CXCL3 to recruit neutrophils; infiltrating neutrophils then sustain BFAR through NF-κB, and BFAR-driven GM-CSF further induces neutrophil PD-L1, consistent with a self-reinforcing immunosuppressive loop and clinically with poor anti-PD-1 response (140). The direction of the YBX1–neutrophil relationship is not, however, uniform: in hepatocellular carcinoma, intracellular CXCL2 binds YBX1 and prevents its nuclear translocation, reducing SREBF2-driven cholesterol biosynthesis and favoring antitumor neutrophil polarization (133). This counter-regulatory finding suggests that restraining YBX1 nuclear access, rather than promoting it, can also reshape myeloid ecology, and should be interpreted as evidence against any universal pro-immunosuppressive role of YBX1.

**Figure 4:**
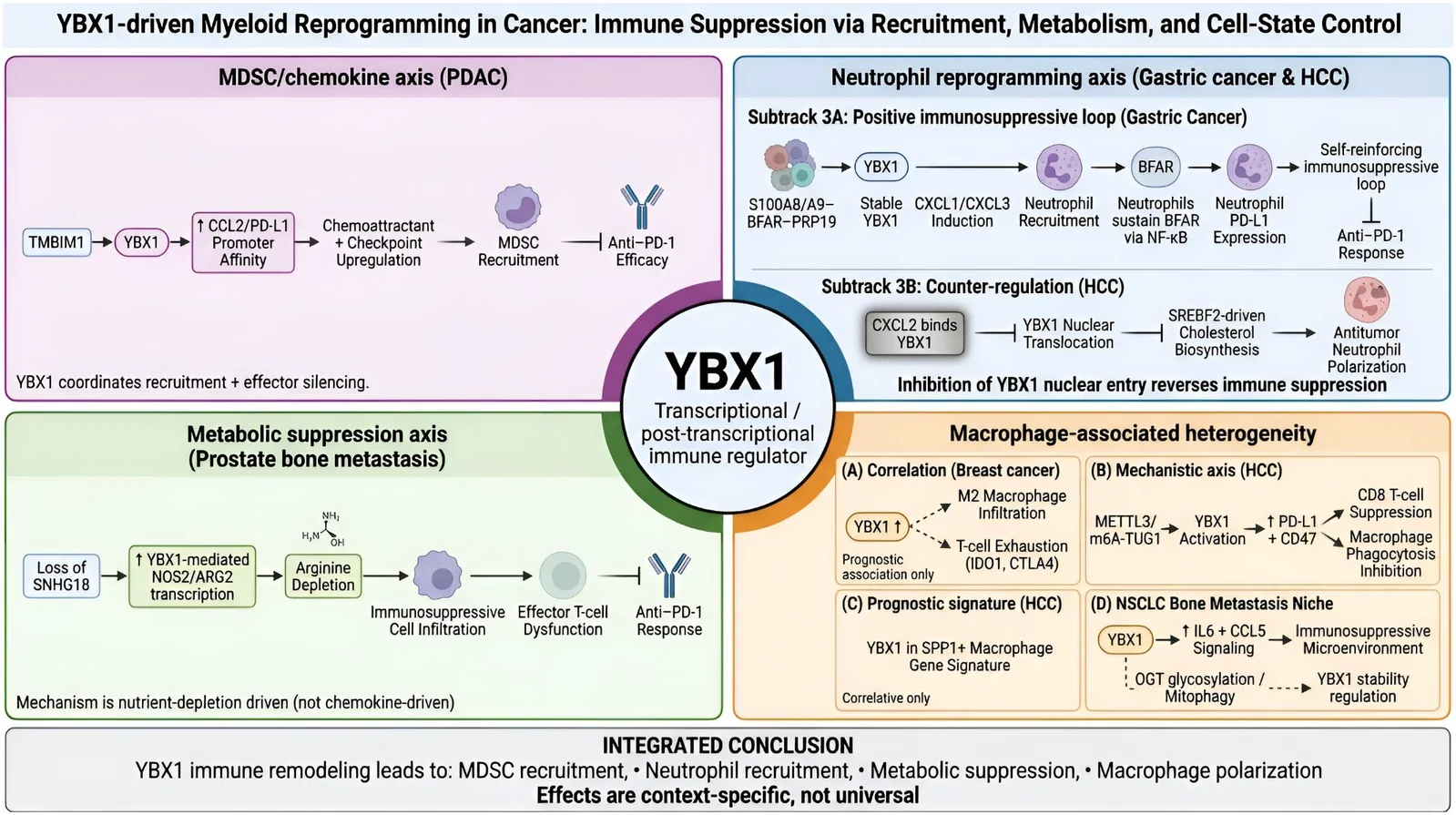
YBX1 orchestrates context-dependent myeloid reprogramming through distinct mechanisms involving chemokine-driven MDSC recruitment, metabolic suppression, neutrophil remodeling, and macrophage-associated immune niches across multiple tumor types. In pancreatic, gastric, hepatocellular, prostate, breast, and lung cancers, YBX1 regulates immune-cell recruitment, polarization, and effector dysfunction through transcriptional and post-transcriptional programs, whereas inhibitory circuits restricting YBX1 nuclear activity can promote antitumor phenotypes. Collectively, these findings indicate that YBX1-mediated immune remodeling is tumor-specific and operates through diverse, rather than universal, mechanisms of immunosuppression.

Macrophage-related evidence is more heterogeneous and warrants caution. In luminal breast cancer, high YBX1 mRNA correlates with M2 macrophage infiltration and T-cell exhaustion markers including IDO1 and CTLA4, but this association is prognostic rather than mechanistically resolved (154). In hepatocellular carcinoma, METTL3/m6A-mediated upregulation of lncRNA TUG1 engages YBX1 to activate PD-L1 and CD47 transcriptionally, simultaneously suppressing CD8+ T-cell activity and macrophage phagocytosis (125). A separate HCC analysis places YBX1 within a seven-gene SPP1+ macrophage-associated prognostic signature, which is correlative rather than mechanistic and should be interpreted accordingly (155). Wider niche evidence comes from bone-metastatic non-small cell lung adenocarcinoma, where YBX1 has been associated with immunosuppressive microenvironment formation through IL6 and CCL5 signaling and is itself regulated by OGT-dependent glycosylation and mitophagic degradation, supporting a context-specific rather than generalizable model (152). Together, these observations indicate that YBX1-linked immune remodeling extends from MDSC and neutrophil recruitment through metabolic suppression to macrophage polarization, in tumor-specific combinations rather than implying a universal mechanism (**Figure 4**).

### 6.5 Lymphocyte, NK-cell, and immune-effector circuits

Beyond checkpoint and myeloid axes, the available evidence suggests that YBX1-linked programs can shape how tumor cells are sensed, attacked, or tolerated by lymphoid and innate effectors, although the direction of this influence is context-dependent rather than uniformly suppressive. The clearest NK-evasion mechanism comes from prostate cancer, where PSAT1 enhances YBX1 phosphorylation through MARK2 and promotes its nuclear translocation; nuclear YBX1 in turn upregulates PSAT1, forming a positive feedback loop, and concurrently activates HLA-E transcription to suppress NK-cell killing, identifying the PSAT1–YBX1–HLA-E axis as a tractable target for NK cytotherapy in immune-cold tumors (40). The relationship between YBX1 and CD8+ T-cell biology is, however, not unidirectional. In lung adenocarcinoma, MIR155HG interacts with and stabilizes YBX1 by dampening its ubiquitination, enabling YBX1-dependent transcription of CCL5 and recruitment of CD8+ T cells; in the same setting, MIR155-associated PD-L1 induction may restrain those recruited cells, an interplay that should be interpreted as context-dependent rather than purely immunosuppressive, particularly since high MIR155HG expression correlates with better immunotherapy response and combines synergistically with PD-L1 blockade (151).

A more conventional dual-checkpoint mode is illustrated in hepatocellular carcinoma, where the lncRNA TUG1 engages YBX1 to transcriptionally upregulate both PD-L1 and CD47, simultaneously dampening CD8+ T-cell activity and macrophage phagocytic clearance, coupling adaptive escape with “don’t-eat-me” signaling within a single YBX1-anchored circuit (125). Comparator immune-ecology evidence from prostate cancer indicates that tRF-3017b can drive CTL resistance and M2 macrophage polarization through cytokine–STAT3 signaling, but this work does not establish a direct YBX1 mechanism and should be cited as parallel rather than YBX1-linked (119). Finally, immune-cell intrinsic data show that YBX1 binds immunoglobulin mRNAs in B cells and enhances their translation, supporting humoral responses; this is contextual evidence for YBX1 as a broader immunoregulatory RNA-binding protein rather than direct tumor escape biology (149). Together, these observations indicate that YBX1 may regulate effector immunity through RNA stability, transcriptional control, post-transcriptional translation, and checkpoint expression in tumor-specific combinations.

### 6.6 YBX-family circuits and immunotherapy response

Response to immune-checkpoint blockade in YBX1-active tumors appears to depend not only on YBX1-specific circuits but, in some contexts, on broader Y-box family logic that engages YBX1 and YBX3 as cooperative components. At the YBX1-specific end of this continuum, the available evidence indicates that YBX1 promoter occupancy at PD-L1 can be modulated either pharmacologically or by partner proteins: in lung cancer, CDKL1 binds YBX1 and reduces its affinity for the PD-L1 promoter, lowering PD-L1, activating CD8+ T cells, and potentiating radioimmunotherapy (135). A parallel YBX1-only axis operates under chemotherapy pressure in hepatocellular carcinoma, where YBX1/PD-L1 induction couples multidrug resistance with immune escape; YBX1 targeting restores cytotoxic CD8+ T-cell activity and reduces MDSC and Treg infiltration, supporting a model in which immune escape and chemoresistance can be jointly reversed by disrupting a single YBX1 node (78). In clear cell renal cell carcinoma, the NSUN5–circPPAP2A–YBX1 axis adds an RNA-modification layer: m5C-modified circPPAP2A is recognized and stabilized by YBX1, enhancing its phosphorylation and nuclear translocation, activating HIF-2α, suppressing CD8+ T-cell infiltration via CCL5 downregulation, and promoting T-cell exhaustion via TGFB1, with axis inhibition sensitizing tumors to anti-PD-1 therapy (122).

**Figure 5:**
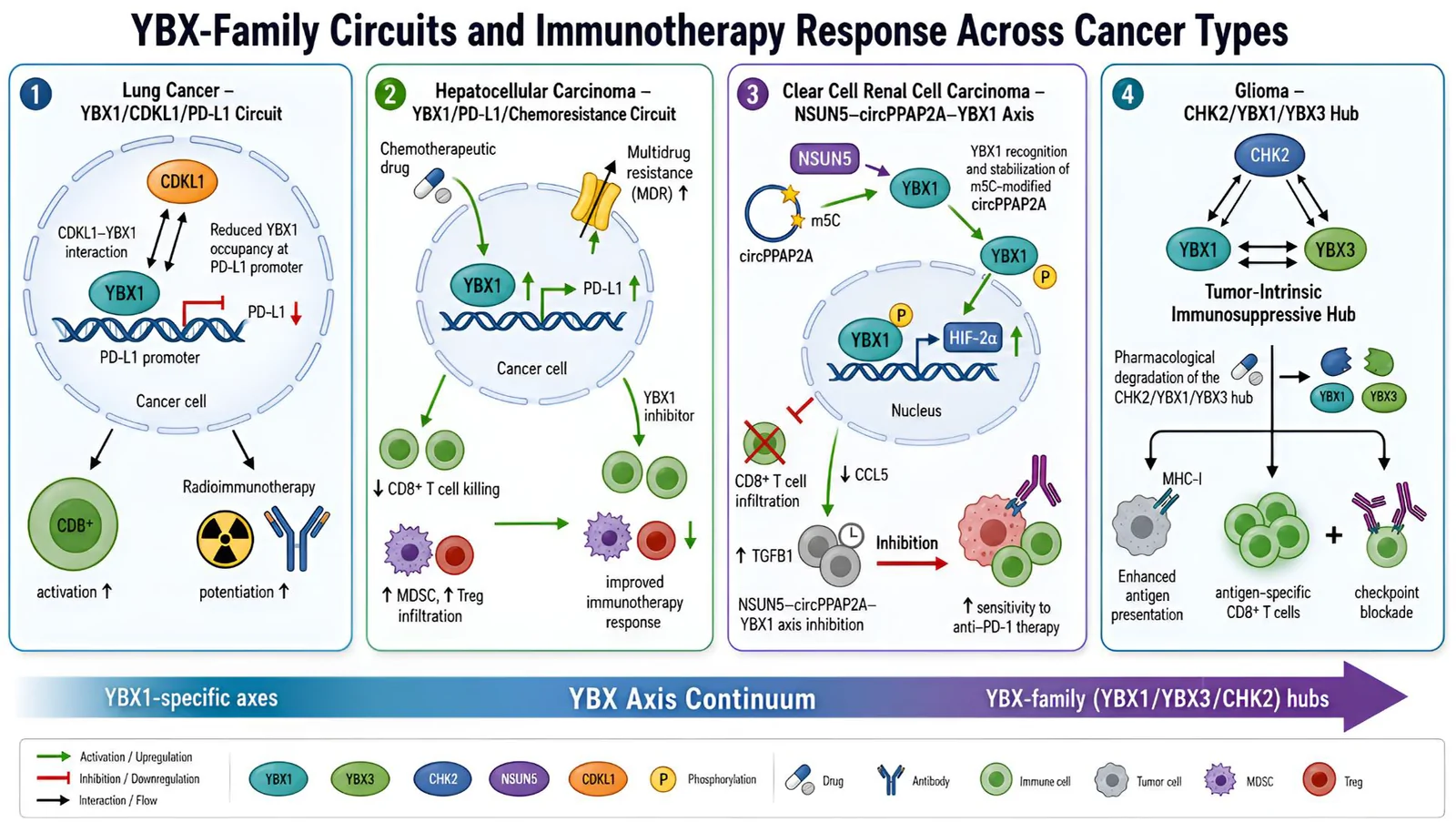
YBX-family circuits shaping immunotherapy response across cancer types. At the YBX1-specific end of the continuum, lung cancer, hepatocellular carcinoma, and clear cell renal cell carcinoma rely on distinct YBX1-centered mechanisms that regulate PD-L1 expression, immune escape, and sensitivity to anti-PD-1 therapy. In glioma, reciprocal interactions among CHK2, YBX1, and YBX3 form a tumor-intrinsic immunosuppressive hub whose pharmacological disruption enhances antigen presentation, expands antigen-specific CD8+ T cells, and potentiates checkpoint blockade, illustrating how tumor-specific YBX-family configurations may determine immunotherapy responsiveness.

The clearest family-level evidence comes from glioma, where CHK2, YBX1, and YBX3 reciprocally regulate one another and form a tumor-intrinsic immunosuppressive hub; pharmacological degradation of this hub enhances antigen presentation, expands antigen-specific CD8+ T cells, and synergizes with checkpoint blockade in preclinical models (148). These observations are consistent with a model in which immunotherapy responsiveness may be shaped by which YBX-family configuration a tumor relies on, although the evidence remains preclinical and tumor-type-specific and should be interpreted cautiously (**Figure 5**).

## 7 Layer 3b: metabolic coupling: lactate, glycolysis, ferroptosis, and nutrient circuits

### 7.1 Lactate–lactylation–glycolysis feedback: metabolism as a YBX1 state-setter

Lactate is the conceptual entry point linking metabolism to YBX1 state regulation, because the available evidence places it both upstream and downstream of YBX1 activity; the metabolic module is mapped by tumor type, dominant circuit, phenotypic output, and therapeutic node in **Table 5**. In intrahepatic cholangiocarcinoma, lactate induces YBX1 nuclear translocation; YBX1 then transcriptionally activates and stabilizes LDHB mRNA, and LDHB converts lactate to pyruvate to feed the TCA cycle, supporting ATP production and proliferation (82). This describes a self-reinforcing circuit in which the metabolite that activates YBX1 is also the substrate processed by the enzyme YBX1 induces, the clearest bidirectional lactate–YBX1 relationship in the literature reviewed here.

**Table 5:** Layer 3b: tumor-by-circuit map of YBX1 dependency, metabolic module.

Tumor type	Dominant module	Key YBX1 circuit	Phenotypic output	Therapeutic node & agent	Evidence	Ref.
5-A. Lactate and lactylation circuits: metabolism as a YBX1 state-setter						
Bladder cancer	Lactylation	H3K18la → YBX1 (and YY1) transcription	Cisplatin resistance in a glycolytic cell subpopulation	H3K18la/glycolysis inhibition (single-cell-defined subset)	M (single-cell)	(83)
Colorectal cancer	Lactate	TROP2 → YBX1–HIF-1α arm → lactate → H3K18la (feedback)	Lactate production; liver-metastatic progression	HIF-1α inhibition (acriflavine); glycolysis blockade	M (YBX1 = one arm)	(84)
Esophageal squamous cell carcinoma	Lactate	NIPAL1–HCK–p-LDHA(Y10)–lactate–p300–H3K18la loop	Immune evasion (CD8+ impairment); tumor growth	HCK or p300 inhibition (+ anti-PD-1 synergy)	M (no YBX1 node §)	(158)
Hepatocellular carcinoma	Lactate	lactate – H3K18la – KIF20A – c-Myc – PD-L1	PD-L1 ↑, immune evasion, anti-PD-1 resistance	Glycolysis inhibition + anti-PD-1 (synergy)	M (no YBX1 node §)	(157)
Intrahepatic cholangiocarcinoma	Lactate/glycolysis	YBX1–LDHB (lactate induces YBX1 nuclear entry; bidirectional)	Lactate → pyruvate → TCA flux/ATP; proliferation, initiation	YBX1–LDHB axis (preclinical target); LDHB knockout	M	(82)
5-B. Glycolytic and hypoxic nodes: LDHA, LDHB, HKDC1, PFKFB4, HIF1-α						
Clear cell renal cell carcinoma	Glycolysis	YBX1–LDHA–NF-κB	Glycolysis, NF-κB activation, proliferation	LDHA silencing/LDHA inhibitor	M	(80)
Gastric cancer	Glycolysis	YBX1/STC1/PI3K–Akt/HK2	Glycolysis; oxaliplatin resistance	Bufalin (direct YBX1 binder) — restores oxaliplatin sensitivity	M	(156)
Hepatocellular carcinoma	Hypoxia	lncRNA USP2-AS1 → YBX1 → HIF1-α mRNA translation	Hypoxic growth; modulates lenvatinib sensitivity	HIF1-α inhibitor + lenvatinib (combination)	M (scaffold detail, **Table 2C**)	(73)
Lung squamous cell carcinoma	Glycolysis	THOC3–YBX1–PFKFB4 (THOC3 exports mRNA; YBX1 stabilizes m5C-3′UTR)	Glycolysis; growth, migration	THOC3 (proposed target)	M	(159)
Pancreatic ductal adenocarcinoma	Glycolysis	Menin–YBX1–HKDC1 (Menin promotes YBX1 nuclear entry)	HKDC1-dependent glycolytic regulation	Menin–YBX1–HKDC1 axis (proposed)	M (localization detail, **Table 1C**)	(76)
Papillary thyroid carcinoma	Glycolysis	SOX12–YBX1–LDHA → TGF-β	Metastasis (migration/invasion)	SOX12–YBX1–LDHA node (proposed); LDHA knockdown	M	(81)
Prostate cancer	Hypoxia	HIF-1α/YBX1 → NonO/p54nrb upregulation (hypoxia-enhanced)	↑ NonO expression (more pronounced under hypoxia)	Non-specific (*in vitro* mechanistic)	M (*in vitro* only)	(160)
5-C. Ferroptosis, lipid/redox buffering, and therapy-tolerant metabolism						
Colorectal cancer	Ferroptosis	USP5 → YBX3 degradation → SLC7A11 stabilization	Ferroptosis resistance, survival	USP5 targeting; ferroptosis induction	M (YBX3, family-level)	(143)
Gastric cancer	Ferroptosis	ODC1–YBX1–SLC7A11	Suppressed lipid peroxidation; 5-FU resistance	Erastin (SLC7A11 inhibitor) + 5-FU; ODC1 inhibition	M	(142)
Hepatocellular carcinoma	Lipid/cholesterol	YBX1–SREBP2 (↑); ABCA1 (↓)	Cholesterol accumulation → RTK stabilization, PI3K/Akt/mTORC1 & EMT; sorafenib resistance	SU056/Betulin (YBX1 or SREBP2 inhibition) — restores sorafenib sensitivity	P (abstract)	(136)
Lung adenocarcinoma	Ferroptosis	YBX1 – PI3K/AKT/mTOR (GPX4, SLC7A11 ↓ on YBX1 silencing)	Ferroptosis-related changes; proliferation/migration	YBX1 silencing; LY294002 phenocopy (preclinical)	P (single line, A549)	(161)
Pancreatic ductal adenocarcinoma	Ferroptosis	SUZ12–YBX1 → H3K27me3 at NCOA4 promoter (silences NCOA4)	↓ labile iron, ↑ antioxidant capacity; gemcitabine resistance	SUZ12 knockout + gemcitabine (synergy)	M	(162)
5-D. Nutrient, nucleotide, and metabolic–epigenetic circuits						
Hepatocellular carcinoma	Metabolic–epigenetic	DNMT2–YBX1–m5C–ACLY → acetyl-CoA/histone acetylation → Notch	Lenvatinib resistance	ACLY inhibition + lenvatinib	M	(124)
Lung adenocarcinoma	Nutrient/nucleotide	Uridine metabolism → YBX1 K137 lactylation (stabilizes) → FBL	Ribosome biogenesis, proliferation, migration, stemness	Uridine-pathway targeting (proposed); YBX1/FBL knockdown	M	(53)
Pancreatic ductal adenocarcinoma	Glycolysis/stemness	circRREB1 → YBX1 nuclear entry → WNT7B (PGK1 glycolytic arm is YBX1-independent)	Stemness (Wnt/β-catenin); glycolysis via PGK1 (YBX1-independent)	circRREB1 (biomarker/target)	M (YBX1 arm = WNT7B/stemness only)	(163)
Prostate cancer (bone metastasis)	Arginine → immune	SNHG18 ↓ → loss of TRIM21-mediated YBX1 degradation → YBX1 → NOS2/ARG2	Arginine depletion; immunosuppressive niche; ↓ effector T cells; ↓ anti-PD-1 response	SNHG18 overexpression enhances anti-PD-1; SNHG18 as ICB-response biomarker	M (immune output; see Section 6.4)	(153)

Rows are grouped into four thematic bands corresponding to Sections 7.1–7.4 and, within each band, ordered alphabetically by tumor type. Evidence classification: M, mechanistic evidence supported by direct molecular interaction and functional perturbation with rescue and/or *in vivo* validation; A, associative evidence based on correlation, signatures, or database-derived inference without demonstrated directionality; P, preliminary evidence including conference abstracts, limited models, or non-peer-reviewed observations. § denotes a lactate–lactylation circuit reported without an explicit YBX1 node; these entries are included as contextual evidence for the same regulatory logic and should not be read as YBX1-dependent. Arrows denote direction of change (↑ increase, ↓ decrease) or sequential steps within a circuit.

Direct YBX1-dependent metabolic circuits diverge by tumor type and downstream consequence. In clear cell renal cell carcinoma, YBX1 interacts with and transcriptionally regulates LDHA, with downstream NF-κB activation supporting proliferation (80). In papillary thyroid carcinoma, SOX12 upregulates YBX1 and recruits it to the LDHA promoter, engaging TGF-β–dependent metastasis (81). In pancreatic ductal adenocarcinoma, Menin facilitates YBX1 nuclear translocation to activate HKDC1 transcription (76). In oxaliplatin-resistant gastric cancer, YBX1 activates STC1, engaging PI3K/Akt–HK2 signaling and glycolysis-linked chemoresistance reversible by bufalin (156). These reports support the possibility that YBX1 can be wired into glycolysis through several non-equivalent nodes, LDHA, LDHB, HKDC1, and STC1/HK2, with adaptive outputs that include proliferation, metastasis, and drug tolerance rather than a single canonical phenotype.

Lactylation studies provide contextual evidence that lactate-rich states can be encoded epigenetically, although their YBX1 relevance is uneven. In bladder cancer, H3K18 lactylation drives transcription of YBX1 (and YY1), which together support cisplatin resistance in a glycolysis-associated cell subpopulation (83). In colorectal cancer, a TROP2-induced H3K18la loop is sustained in part through a TROP2–YBX1–HIF-1α arm that drives lactate production (84). In contrast, the H3K18la–KIF20A–c-Myc–PD-L1 axis in hepatocellular carcinoma (157) and the NIPAL1–HCK–LDHA–lactate–p300–H3K18la loop in esophageal squamous carcinoma (158) describe metabolic-epigenetic feedback without an explicit YBX1 node and should not be read as YBX1-dependent, even though they share the same broader logic of lactate as an epigenetic signal.

Read together, direct YBX1–metabolic axes and broader lactate–lactylation feedback are consistent with a model in which lactate accumulation acts as an information carrier, sometimes through YBX1 directly, sometimes through histone lactylation that operates on YBX1 or on parallel transcriptional nodes, but tumor-type specificity must be preserved. In YBX1-dependent tumors, metabolism does not merely supply energy; it encodes stress states into RNA fate decisions.

### 7.2 Glycolytic and hypoxic RNA circuits: LDHA, LDHB, HKDC1, PFKFB4, and HIF1-α

Glycolytic and hypoxic adaptation in YBX1-dependent tumors is increasingly understood not as a generic metabolic phenotype but as a set of RNA- and transcription-level programs whose outputs are shaped by specific YBX1 states. Across the available studies, four distinct metabolic nodes, LDHB, LDHA, HKDC1 and PFKFB4, are engaged through mechanisms that converge in their pro-tumor consequences but differ in the layer at which YBX1 operates. The first three were established in Section 7.1 and are not restated here: LDHB in intrahepatic cholangiocarcinoma is dual-regulated, transcriptionally and post-transcriptional (82), LDHA in clear cell renal cell carcinoma and papillary thyroid carcinoma is transcriptional (80, 81); and HKDC1 in pancreatic ductal adenocarcinoma is gated by Menin-facilitated nuclear import, so that compartmental control of YBX1 itself functions as a metabolic switch (76).

The RNA-interface character of YBX1 is most clearly demonstrated at PFKFB4, where THOC3 supports nuclear export of PFKFB4 mRNA while YBX1 stabilizes the transcript by recognizing m5C sites in its 3′UTR, an explicit example of export-reader cooperation directing glycolytic capacity (159). Hypoxia further reshapes these outputs at the translational layer: under low oxygen in hepatocellular carcinoma, the lncRNA USP2-AS1 enhances YBX1 binding to HIF1-α mRNA, elevating HIF1-α protein levels (73), while in prostate cancer cells YBX1 augments NonO/p54nrb expression more strongly under hypoxic than normoxic conditions, in a setting where HIF-1α itself drives NonO transcription (160). Taken together, these observations are consistent with a framework in which YBX1 links metabolic and oxygen stress to selective RNA fate; the directionality and partner dependencies, however, are tumor-type-specific and should be interpreted cautiously rather than generalized.

### 7.3 Ferroptosis, lipid/redox buffering, and therapy-tolerant metabolism

Therapy-tolerant metabolism in YBX1-linked tumors appears to operate through a coordinated set of survival-buffering circuits that mitigate the oxidative, iron, and lipid-peroxidation stresses imposed by chemotherapy and targeted agents, rather than through a single anti-ferroptotic mechanism. At the lipid layer, the available evidence supports a model in which YBX1 drives cholesterol metabolic rewiring in hepatocellular carcinoma by transcriptionally activating SREBP2 and suppressing the cholesterol efflux transporter ABCA1; the resulting intracellular cholesterol accumulation appears to stabilize membrane receptor tyrosine kinases and sustain downstream PI3K/Akt/mTORC1 and EMT signaling, providing a membrane- and signaling-level buffer that contributes to sorafenib resistance (136). At the redox layer, a more direct ferroptosis-resistance mechanism has been characterized in gastric cancer, where ODC1 interacts with YBX1 through its PLPDE_III_ODC domain and the complex binds the SLC7A11 promoter to enhance SLC7A11 transcription; this axis suppresses lipid peroxidation and glutathione depletion and may contribute to 5-FU resistance, an interpretation supported by the observation that pharmacological SLC7A11 inhibition with erastin synergizes with 5-FU and restores chemosensitivity (142). Complementary observations in lung adenocarcinoma associate YBX1 with PI3K/AKT/mTOR signaling and ferroptosis-related changes, including GPX4 and SLC7A11 downregulation upon YBX1 silencing, but these findings derive predominantly from A549-based models and should be interpreted cautiously rather than generalized (161).

A distinct, epigenetic mode of ferroptosis avoidance is illustrated in pancreatic ductal adenocarcinoma, where SUZ12 recruits YBX1 to the NCOA4 promoter and deposits H3K27me3, silencing the ferritinophagy receptor NCOA4; the resulting limitation of labile iron release is consistent with enhanced antioxidant capacity and gemcitabine resistance (162). Contextually, Y-box family involvement extends beyond YBX1: in colorectal cancer, USP5-mediated lysosomal degradation of YBX3 stabilizes SLC7A11 and suppresses ferroptosis, an axis that should be read as Y-box family-level evidence rather than conflated with YBX1 biology (143). Together, these circuits are consistent with a framework in which YBX1-dependent tumors may engage lipid, redox, and epigenetic buffers in tumor-type-specific combinations, enabling persistence under therapeutic pressure rather than universally blocking ferroptosis.

### 7.4 Nutrient, nucleotide, and metabolic–epigenetic circuits

The metabolic involvement of YBX1 extends beyond glycolysis and lactate handling into nutrient-depletion, nucleotide–ribosome, metabolic–epigenetic, and stemness-linked circuits, although these programs remain tumor-type-specific rather than constituting a single integrated YBX1 metabolic axis. The available evidence supports a model in which YBX1 protein abundance is itself nutrient-tunable: in lung adenocarcinoma, uridine metabolism appears to stabilize YBX1 through K137 lactylation, enabling YBX1 to transactivate FBL and drive ribosome biogenesis, proliferation, migration, and stemness, coupling a nucleotide-derived metabolic input to a translation-capacity output (53). In prostate cancer bone metastasis, the regulatory direction is opposite: loss of the lncRNA SNHG18 removes a TRIM21-dependent brake on YBX1 degradation, permitting YBX1-mediated transcriptional activation of NOS2 and ARG2, arginine depletion, infiltration of immunosuppressive cells, impaired effector T-cell function, and reduced anti-PD-1 responsiveness; here YBX1-linked metabolism is most clearly read as an immune-microenvironmental program rather than a cell-intrinsic energetic one (153).

A metabolic–epigenetic feedback mode is illustrated in hepatocellular carcinoma, where DNMT2 and YBX1 cooperate to stabilize ACLY mRNA through m5C modification, raising intracellular acetyl-CoA and histone acetylation and activating Notch signaling in a circuit consistent with lenvatinib resistance (124). In pancreatic ductal adenocarcinoma, circRREB1 supports glycolysis chiefly through PGK1, a YBX1-independent arm, while a parallel YBX1-dependent arm involves circRREB1-facilitated YBX1 nuclear translocation and WNT7B transactivation that maintains stemness, and this distinction should be interpreted cautiously (163). Collectively, these observations indicate that YBX1 may contribute to tumor metabolism not only as a glycolytic regulator but as a node integrating nutrient sensing, translation capacity, epigenetic acetylation, and stemness in tumor-specific combinations.

### 7.5 A unified metabolic framework: YBX1 as a stress-conditional metabolic router

The circuits described in Sections 7.1–7.4 are usually reported one pathway at a time, but read together they describe a single logic rather than a catalog: YBX1 does not drive one metabolic program; it routes flux toward whichever adaptive program the prevailing stress demands. Four features organize this behavior, and two boundaries define its limits. The organizing input is oxygen-and-energy stress transduced largely through a shared axis. Hypoxia is the clearest case: under low oxygen, the lncRNA USP2-AS1 enhances YBX1-mediated HIF1-α translation (73), the NSUN5/circPPAP2A/YBX1 circuit activates HIF-2α (122), and YBX1 augments NonO expression more strongly under hypoxic than normoxic conditions (160). Each links an oxygen-sensing input to a YBX1-dependent output, and each feeds the same PI3K/AKT/mTOR axis that Section 5.7 identified as the dominant signaling backbone. Metabolic and oxygen stress are therefore not separate inputs to YBX1 but converge on the same state-setting machinery.

Glycolysis is the best-documented output, and the five non-equivalent entry points catalogued in Sections 7.1–7.2 are the substance of the router claim: a regulator with one output would be a driver, whereas YBX1 selects among entry points according to tumor and stress context. Crucially, where glycolytic flux terminates is also YBX1-shaped: in intrahepatic cholangiocarcinoma, YBX1-induced LDHB converts lactate to pyruvate to feed the TCA cycle and ATP production (82). This is the one point of contact between YBX1 and oxidative phosphorylation, and it is worth stating precisely: YBX1 sets the supply of pyruvate to the mitochondrion, but no evidence assembled here shows YBX1 regulating the electron transport chain or oxidative phosphorylation directly. YBX1 conditions the input to respiration without controlling respiration itself.

Lipid and redox buffering are the output that dominates under therapeutic rather than microenvironmental stress. The cholesterol arm, YBX1-driven SREBP2 activation with ABCA1 suppression, stabilizing membrane RTKs and sustaining PI3K/Akt/mTORC1 (136), and the redox arm, ODC1/YBX1 transactivation of SLC7A11, suppressing glutathione depletion and lipid peroxidation (142), with GPX4 and catalase contributions (123, 161), are mechanistically distinct but functionally convergent: both raise the threshold for therapy-induced ferroptosis. This is where YBX1-dependent metabolism becomes indistinguishable from YBX1-dependent survival, and it is why the ferroptosis-avoidance circuits are developed both here and in the resistance layer (Section 8.3).

The unifying property across all three outputs is closed-loop coupling to the metabolite itself, developed in Section 5.7 and criterion (iv) of **Box 1**: lactate promotes YBX1 nuclear entry and its own further production through H3K18 lactylation (82, 84), and uridine-derived K137 lactylation stabilises YBX1 (53), while lactate-derived H3K18 lactylation returns to its transcription (36, 83). Metabolism is therefore not merely an output of YBX1 but an input that sets its state, which is what distinguishes a stress-conditional router from a constitutive metabolic transcription factor.

**Figure 6:**
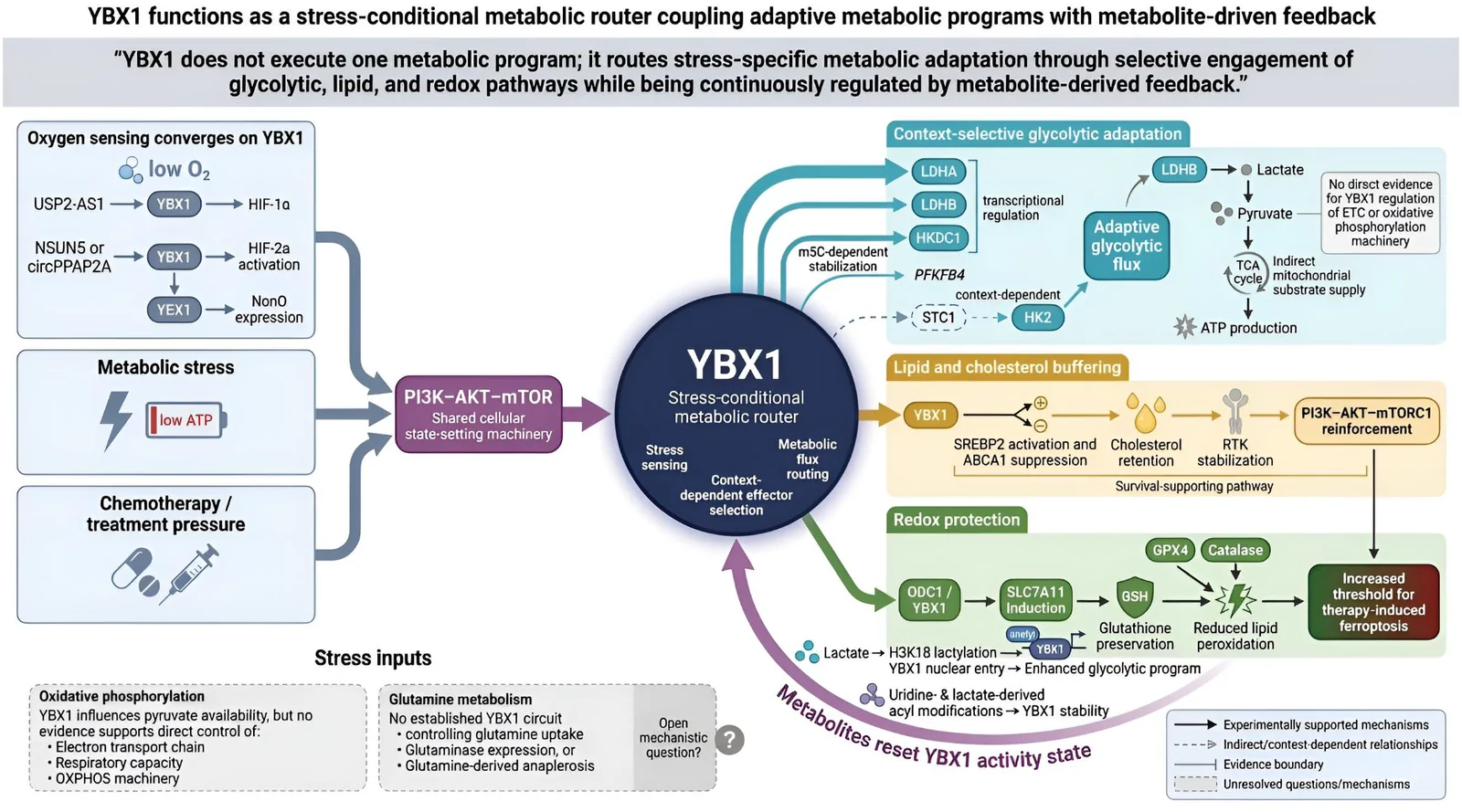
YBX1 as a stress-conditional metabolic router. YBX1 integrates oxygen/energy stress and therapy pressure, largely through the PI3K–AKT–mTOR axis, and selectively redirects metabolic flux toward context-appropriate adaptive programs, including glycolysis, lipid/cholesterol buffering, and redox protection. This routing is further reinforced by metabolite-driven feedback, while current evidence limits YBX1 control of oxidative phosphorylation to indirect pyruvate supply and leaves glutamine metabolism as an unresolved boundary.

Two boundaries should be stated as plainly as the framework. First, oxidative phosphorylation is engaged only indirectly, through pyruvate supply; there is no evidence that YBX1 regulates mitochondrial respiratory capacity, and the framework should not be read as claiming it does. Second, glutamine and glutaminolysis, prominent in the metabolic adaptation of many tumors, are essentially absent from the YBX1 literature assembled here; we identify no circuit in which YBX1 controls glutamine uptake, glutaminase expression, or glutamine-derived anaplerosis. Whether this reflects a genuine boundary of YBX1 biology or an unexamined question cannot be resolved from current evidence, but it is a specific and testable gap: if YBX1 is a general metabolic router, glutamine circuits should exist and have simply not been looked for; if it is a glycolysis-, hypoxia-, and redox-restricted one, their absence is a real feature of its selectivity. Either way, the honest framework is narrower than the full metabolic repertoire, and its edges are informative (**Figure 6**).

## 8 Layer 3c: plasticity and the architecture of therapy resistance

Because plasticity and resistance draw on the same metabolic and immune circuits developed in Layers 3a and 3b, this layer is organized to avoid restating them: where a resistance phenotype is driven by a mechanism already detailed as metabolic (ferroptosis, glycolysis, lipid buffering) or immune (checkpoint, myeloid), we cross-reference rather than repeat, and confine the discussion here to the plasticity- and resistance-specific logic, how these circuits are selected, combined, and deployed under therapeutic stress (**Table 6**).

**Table 6:** Layer 3c: tumor-by-circuit map of YBX1 dependency, plasticity and therapy-resistance module.

Tumor type	Dominant module	Key YBX1 circuit	Phenotypic output	Therapeutic node & agent	Evidence	Ref.
6-A. EMT, stemness, metastatic competence, and cell-cycle plasticity						
Breast cancer	Plasticity (EMT/ceRNA)	PRSS23-AS1 (miR-3176 sponge) → ↑ YBX1	EMT, invasion, proliferation, metastasis	PRSS23-AS1/miR-3176 axis	M	(167)
Breast cancer (TNBC)	Plasticity (Wnt)	YBX1 → Wnt/β-catenin	Proliferation, apoptosis suppression, paclitaxel resistance	YBX1 targeting	M	(164)
Cholangiocarcinoma	Plasticity (NF-κB) + resistance	KIF14 bridges G3BP1/YBX1 → NF-κB	Proliferation, lymphatic metastasis, gemcitabine resistance, immunosuppressive TME	KIF14 (HNF4A-regulated)	M	(146)
Colorectal cancer	Plasticity (NF-κB)	METTL3-m6A-stabilized lnc-CRAT40 recruits YBX1 → RelA promoter → NF-κB	Proliferation, metastasis	lnc-CRAT40	M	(168)
Colorectal cancer	Plasticity (MAPK)	EPS8L2 → S6K1 → pYBX1 nuclear entry → G3BP2 → MAPK	Proliferation, migration	EPS8L2/YBX1/G3BP2 axis	M	(132)
Esophageal squamous cell carcinoma	Plasticity (Wnt)	HOXC-AS3 recruits YBX1 → WNT3A promoter	Proliferation, migration	HOXC-AS3/YBX1 axis	M	(165)
Gastric cancer	Plasticity (Wnt)	LINC00665–YBX1 feed-forward loop → Wnt3a/β-catenin	EMT, proliferation, metastasis	LINC00665/YBX1 loop	M	(139)
Gastric cancer	Plasticity (senescence; direct-inhibition PoC)	YBX1 hub linking mTOR, ROS and DDR	Proliferation; senescence suppression	YBX1 inhibition (“one-two punch” + mTOR senolytic)	M	(171)
Lung adenocarcinoma	Plasticity (NF-κB)	RECQL4 scaffolds YBX1/G3BP1 → NF-κB	Proliferation, migration, invasion	RECQL4 (target)	M	(145)
Lung adenocarcinoma	Plasticity (stemness/metastasis)	YBX1 → MUC1 transcription	Stemness, metastasis	YBX1/MUC1 axis	M	(39)
Non-small-cell lung cancer	Plasticity (metastasis)	HOXC-AS3 suppresses MDM2-mediated YBX1 ubiquitination → HOXC8	Growth, metastasis	HOXC-AS3/YBX1/HOXC8	M	(166)
Non-small-cell lung cancer	Plasticity ¶	circRABL2B–YBX1 inhibits MUC5AC → ↓ stemness	↑ Erlotinib sensitivity (tumor-suppressive)	circRABL2B (exosome-delivered)	M (counter-regulatory) ¶	(170)
Non-small-cell lung cancer	Plasticity ¶	ST7 binds YBX1 and blocks nuclear translocation → ↓ MMP14	↓ EMT and metastasis (tumor-suppressive)	ST7/YBX1/MMP14	M (counter-regulatory) ¶	(134)
Ovarian cancer	Plasticity (cell cycle)	YBX1 → cyclin A1 → G2/M (subset of cell lines only)	Proliferation (cell-line-dependent)	YBX1/cyclin A1	M (cell-line heterogeneous)	(169)
Pancreatic cancer	Plasticity (metastasis)	RRM2 stabilizes YBX1 → TGFBR1/TGF-β	Liver metastasis	RRM2 (target)	M	(144)
Rhabdomyosarcoma	Plasticity (stemness)	MYC–YBX1 mutual regulatory circuit → CD133+	Vincristine-tolerant stem-like cells	MYC inhibitor MYC361i + vincristine	M (zebrafish model)	(138)
6-B. Cytotoxic stress tolerance: chemo-, radio-, platinum-, and DNA-damage resistance						
Bladder cancer	Resistance (cytotoxic)	GEM/DDP-induced NF-κB → OIP5 → TRIP12 degrades PPP1CB → ↑ YBX1 TF activity	Drug-resistance gene transcription; gemcitabine/cisplatin resistance (feedback loop)	OIP5 knockout (engineered CRISPR gene circuit) + chemotherapy	M	(177)
Breast cancer	Resistance (apoptosis)	TRIM25 → K48-Ub degradation of BRD7 → YB1/Bcl-2	Paclitaxel resistance (apoptosis suppression)	TRIM25/BRD7 axis	M	(178)
Breast cancer (MDR)	Resistance ¶	miR-375 (promoter-methylated) targets the YBX1 3′UTR	Multidrug resistance (↑ P-gp); 5-AZA-dC restores miR-375	Demethylation/miR-375 restoration	P (single cell line, MCF-7) ¶	(181)
Cervical adenocarcinoma	Resistance (radio)	CYP4A22-AS1 → YBX1 nuclear entry → PGK1 transactivation	Glycolytic radioresistance	CYP4A22-AS1/PGK1 axis	P (conference abstract)	(172)
Colon adenocarcinoma	Resistance (partner-dependent)	YBX1 oxaliplatin resistance requires NONO and RALY	Oxaliplatin resistance; NONO/RALY depletion reverses	NONO/RALY depletion	M	(182)
Colon cancer	Resistance ¶	miR-137 targets the YBX1 3′UTR	Oxaliplatin resistance; miR-137 restoration sensitizes	miR-137 restoration	M (counter-regulatory) ¶	(180)
Colorectal cancer	Resistance (apoptosis) ¶	MDM1 restrains YBX1 binding to the TP53 promoter → ↑ p53/apoptosis	Chemoradiotherapy sensitivity (MDM1-high = sensitive)	MDM1 (marker); apoptosis inducers if MDM1-low	M (counter-regulatory) ¶	(179)
Epithelial ovarian cancer	Resistance (ubiquitin) ¶	SIAH1 K304-ubiquitinates YBX1 → destabilizes m5C-target mRNAs	Restores cisplatin sensitivity	SIAH1 (sensitizer)	M (counter-regulatory) ¶	(176)
Gastric cancer	Resistance (DNA damage/ROS)	TAGLN2 → AKT–YBX1; ssDNA aggregation, cGAS-STING/ISG induction	Chemo- and radioresistance	Fisetin/MK2206 + cisplatin	M	(147)
High-grade serous ovarian carcinoma	Resistance (direct-inhibition PoC)	SU056 small-molecule YBX1 inhibitor → ↓ mRNA processing, G1 arrest, ↓ drug efflux	Synergy with cisplatin or paclitaxel	SU056 (direct YBX1 inhibitor)	M	(42)
Intrahepatic cholangiocarcinoma	Resistance (stemness)	YBX1 → AKT/β-catenin	Stemness, cisplatin insensitivity	AZD5363/KYA1979K (AKT/β-catenin)	M (tumor-specific stemness, not general cytotoxic tolerance)	(175)
Non-small-cell lung cancer	Resistance (cytotoxic)	IGFL2-AS1 recruits YBX1 → HSPA1A promoter → RAP1	Cisplatin and 5-FU resistance	HSPA1A inhibition	M	(173)
Osteosarcoma	Resistance (DNA damage/ROS)	NSUN6 m5C on PEX1/PEX3 read by YBX1 → peroxisome/catalase → ROS buffering; MARCH8 ubiquitinates NSUN6	Cisplatin resistance	NSUN6/YBX1/PEXs (MARCH8 axis)	M	(123)
Ovarian cancer (carboplatin-resistant)	Resistance (cytotoxic)	circRAD23B sponges miR-1287-5p → ↑ YBX1	Carboplatin resistance	circRAD23B/miR-1287-5p/YBX1	M (cell lines + organoids)	(174)
6-C. Autophagy, PANoptosis suppression, and survival buffering						
Gastric cancer	Resistance (autophagy)	MAZ → YBX1; YBX1 (m5C reader, NSUN2) stabilizes ATG9A	Autophagy; 5-FU resistance	YBX1/ATG9A axis	M (reader detail, **Table 2A**)	(27)
Gastric cancer	Resistance (PANoptosis)	YBX1 inhibits PANoptosis; PPM1B (S314 dephosphorylation) and USP10 (deubiquitination) tune it	Oxaliplatin resistance	PPM1B/USP10/YBX1 axis	M (PANoptosis context-specific)	(50)
Hepatocellular carcinoma	Resistance (autophagy)	circZNF79(5) → BRCC36 (K63-DUB) shields YBX1 from p62-dependent autophagy (AMPK/mTOR)	YBX1 stabilization; proliferation	circZNF79(5)–YBX1–BRCC36	M (also **Table 2B**)	(61)
Non-small-cell lung cancer	Resistance (autophagy)	YBX1 → p110β/Vps34/beclin1	Autophagy; cisplatin resistance	YBX1/p110β axis	M	(184)
6-D. Adaptive bypass in targeted and endocrine therapy resistance						
Breast cancer	Resistance (endocrine bypass)	YBX1 → accelerated ER proteasomal degradation + ERBB2 transactivation	Fulvestrant and tamoxifen resistance	YBX1 targeting	M	(186)
Breast cancer	Resistance (endocrine bypass)	NFIB + YBX1 bind ESR1/FOXA1; FGFR2 augments the interaction	Repressed estrogen responsiveness	FGFR2 inhibition	M	(187)
Breast cancer	Resistance (endocrine bypass)	YBX1–SBF2-AS1 positive feedback → PI3K/AKT/mTOR	Tamoxifen resistance	SBF2-AS1 axis	M (scaffold detail, **Table 2C**)	(75)
Breast cancer (antiestrogen-resistant; TNBC)	Resistance (state/endocrine)	AKT/p70S6K/p90RSK → S102 pYBX1 nuclear entry	Antiestrogen resistance, drug-resistance genes; pYBX1 enriched in TNBC	TAS0612 + everolimus (suppress pYBX1)	M (state detail, **Table 1C**)	(46)
EGFR-mutant NSCLC	Resistance (targeted bypass)	NSUN2 m5C on QSOX1 read by YBX1 → ↑ translation	Intrinsic gefitinib resistance	NSUN2/YBX1/QSOX1	M (reader detail, **Table 2A**)	(66)
Hepatocellular carcinoma	Resistance (targeted bypass)	RIOK1 → YBX1 Ser165 phosphorylation/nuclear entry → JAK2/STAT3	Lenvatinib resistance	RIOK1/YBX1 axis	M	(49)
Hepatocellular carcinoma	Resistance (targeted bypass)	STK24 → MASTL → pYBX1 → PAK4	Lenvatinib resistance	MASTL (MASTL/YBX1/PAK4)	M	(131)
Hepatocellular carcinoma	Resistance (targeted bypass)	circRNA-SORE binds YBX1 and blocks PRP19-mediated ubiquitination; exosome-spread	Sorafenib resistance	circRNA-SORE/YBX1	M (also **Table 2B**)	(117)
Pancreatic ductal adenocarcinoma	Resistance (targeted bypass)	YBX1 → LRP1 → β-catenin/TCF3 → RRM1	Gemcitabine resistance	SU056 + gemcitabine	M	(185)
Prostate cancer	Resistance (endocrine bypass) → immune	FLII ↓ → YBX1 nuclear entry → PD-L1	Enzalutamide resistance + immune evasion (↑ Treg/MDSC, ↓ CD8)	FLII restoration	M	(43)

Rows are grouped into four thematic bands corresponding to Sections 8.1–8.4 and, within each band, ordered alphabetically by tumor type. Evidence classification: M, mechanistic evidence supported by direct molecular interaction and functional perturbation with rescue and/or *in vivo* validation; P, preliminary evidence including conference abstracts, limited models, or non-peer-reviewed observations. ¶ denotes a counter-regulatory circuit, one in which YBX1 activity opposes the malignant/immune-evasive phenotype, or in which restraining YBX1 is protective; retained deliberately as evidence against a universal oncogenic role.

### 8.1 EMT, stemness, metastatic competence, and cell-cycle plasticity

Some of the EMT programs discussed here arise from metabolic rewiring. For example, the metabolic basis of SREBP2/cholesterol-driven EMT is detailed in Section 7.3; the focus here is therefore on how this program promotes phenotypic plasticity, stemness, and adaptation under therapeutic pressure. The available evidence supports a model in which YBX1 functions as an adaptive RNA and transcriptional hub that converts intracellular stress into the phenotypic plasticity from which therapy resistance ultimately emerges; the plasticity and therapy-resistance circuits are mapped by tumor type, dominant circuit, phenotypic output, and therapeutic node in **Table 7**. Rather than acting as a uniform driver of a single resistance program, YBX1 appears to license context-dependent transitions across epithelial–mesenchymal transition (EMT), stem-like states, metastatic competence, and stress-adapted cell-cycle behavior, with the dominant phenotype shaped by partner availability, post-translational state, and tumor type.

**Table 7:** Validated versus speculative status of YBX1-directed therapeutic concepts.

Therapeutic concept	Agent/approach	What has been shown (validated)	What remains inference (speculative)	Ref.
Direct inhibition				
SU056 (direct YBX1 inhibitor)	Small molecule; ovarian PDX/syngeneic	Induces G1 arrest; inhibits proliferation, apoptosis resistance, drug efflux, and MDR1; modulates RNA-processing pathways; restrains ovarian cancer progression *in vivo*; and enhances the antitumor effect of paclitaxel	Selectivity over YBX2/YBX3 untested; no clinical data; mechanism of engagement not fully resolved	(42, 183)
FKA-9i (Flavokawain A derivative)	Multi-target small molecule	Binds and disrupts LRPPRC–YBX1–RPN1; cell-cycle arrest, ROS, apoptosis; chemo/glycolysis synergy	Not a YBX1-selective probe — a multi-target scaffold; on-target contribution of YBX1 not isolated	(190)
Glycine (repurposed CSD binder)	Computational repurposing + validation	Low-toxicity, cold-shock-domain-interacting molecule; feasibility demonstrated	Low affinity; not potency-optimized; clinical readiness not established	(191)
Functional YBX1 suppression	Genetic knockdown, gastric cancer	↑ ROS, disrupted DNA-damage repair, mTOR modulation, senescence vulnerability	“One-two punch” combination is a proposed strategy, not a tested regimen	(171)
State disruption (target the active pool)				
Anti-phospho-YBX1 (S102 axis)	TAS0612 + everolimus, breast cancer	Suppresses pYBX1; overcomes antiestrogen resistance in preclinical models; pYBX1 IHC enriched in recurrent/TNBC tumors	pYBX1 not prospectively validated as a predictive biomarker; agents act on upstream kinases, not YBX1 directly	(46)
Upstream pathway suppression	BKM120 (PI3K), colorectal	Partially reverses YBX1-driven proliferation/migration	Indirect; effect not YBX1-specific; compensatory signaling not addressed	(192)
Dual kinase targeting	RSK + Akt, KRAS-mutant colorectal	Overcomes compensatory Akt activation; sensitizes to 5-FU; nuclear pYBX1 marks the resistant state	Combination not clinically tested; durability of dual blockade unknown	(137)
Localization disruption	Celecoxib/YAP–YBX1 shuttling, HCC	Triggers nucleocytoplasmic shuttling, ↓ COX-2, mitochondrial apoptosis	Mechanism is one of several celecoxib effects; YBX1-specificity not isolated	(193)
Circuit breaking (match the dominant dependency)				
Immune-axis co-targeting + ICB	NSUN5/circPPAP2A/YBX1; CDKL1/PD-L1; YBX1 KD	In founding studies, axis disruption restores CD8+ activity and synergizes with anti-PD-1/PD-L1	Predicted pharmacodynamic readouts (Treg/MDSC/M2 contraction) not yet reproduced in independent prospective cohorts	(78, 122)
Metabolic-axis targeting	Bufalin (STC1/HK2); erastin + 5-FU (ODC1/SLC7A11)	Direct YBX1 binding (bufalin); ferroptosis induction restores chemosensitivity in preclinical gastric models	Tumor-type-restricted; combination indices not established in patients	(142, 156)
Adaptive-bypass targeting	SU056 + gemcitabine; SU056/Betulin + sorafenib	Reverses LRP1/β-catenin/RRM1 and SREBP2-mediated resistance in preclinical models	Preclinical proof of concept only; no dosing, safety, or sequencing data	(136, 185)
Cross-cutting requirements (apply to all of the above)				
Intra-family selectivity	Any CSD-directed agent	Cold-shock domain conservation across YBX1/2/3 is documented	No agent shown to spare tumor-suppressive YBX3 (e.g. TTC36–YBX3–SPRED1)	(189)
Immunotoxicity control	Any systemic YBX1 inhibitor	YBX1 has defined roles in B-cell (Ig translation) and NK (HLA-E) biology	On-target immune impairment untested; requires immunocompetent-model assessment and tumor-restricted delivery	(40, 149)
Predictive biomarkers	pYBX1, N:C ratio, m5C-reader context, scaffold identity	Analytically measurable; associated with resistant states retrospectively	None prospectively validated against response to a YBX1-directed agent	—

Concepts are grouped by strategy (direct inhibition, state disruption, circuit breaking, and cross-cutting requirements). For each, the third column states what has been experimentally demonstrated and the fourth states what remains inference from mechanism. No YBX1-directed agent has entered clinical trials; all entries are preclinical. The distinction drawn is between what has been shown and what has been proposed, not between promising and unpromising approaches.

Several lncRNA–YBX1 circuits converge on Wnt/β-catenin-driven plasticity. In triple-negative breast cancer, YBX1 may contribute to proliferation, apoptosis suppression, and paclitaxel resistance through Wnt/β-catenin activation (164). In esophageal squamous carcinoma, HOXC-AS3 recruits YBX1 to the WNT3A promoter to support proliferation and migration (165), and a feed-forward LINC00665–YBX1 loop activating Wnt3a/β-catenin operates in gastric cancer to drive EMT and metastasis (139). A distinct HOXC-AS3–YBX1–HOXC8 axis in non-small-cell lung cancer (NSCLC) is consistent with lncRNA-mediated suppression of MDM2-dependent YBX1 ubiquitination, stabilizing YBX1 and amplifying its metastatic transcriptional output (166), while in breast cancer PRSS23-AS1 appears to act as a miR-3176 sponge to elevate YBX1 and promote EMT-associated invasion (167). In this context, YBX1 integrates ncRNA inputs into a transcriptional grammar of plasticity rather than enforcing a universal EMT program.

A parallel set of mechanisms routes adaptation through NF-κB and MAPK adaptive bypass. The RECQL4–YBX1/G3BP1 scaffold activates NF-κB in lung adenocarcinoma to support proliferation, migration, and invasion (145), and KIF14 similarly bridges G3BP1/YBX1 to drive NF-κB-mediated proliferation, lymphatic metastasis, and gemcitabine resistance with an attendant immunosuppressive microenvironment in cholangiocarcinoma (146). In colorectal cancer, lnc-CRAT40 recruits YBX1 to the RelA promoter to initiate NF-κB transcription (168), while EPS8L2 enhances S6K1-dependent YBX1 phosphorylation and nuclear translocation to activate G3BP2 transcription and MAPK signaling (132). Stemness and metastatic competence acquire transcriptional anchoring through YBX1-mediated MUC1 induction in lung adenocarcinoma (39) and through RRM2-driven YBX1 stabilization, which activates TGF-β signaling to support pancreatic cancer liver metastasis (144). The MYC–YBX1 mutual regulatory circuit further sustains CD133-positive vincristine-tolerant stem-like cells in rhabdomyosarcoma, illustrating a drug-tolerant stress state rather than a classical target–drug interaction (138). At the cell-cycle level, YBX1 supports G2/M progression via cyclin A1 in a subset of ovarian cancer cell lines but not in others, indicating that its proliferative contribution should be interpreted cautiously across tumor backgrounds (169).

Counter-regulatory observations argue against universal causality. In lung cancer, circRABL2B interacts with YBX1 to inhibit MUC5AC, reduce stemness, and enhance erlotinib sensitivity (170), and ST7 binds YBX1 to block its nuclear translocation, suppress MMP14, and restrain EMT and metastasis in NSCLC (134). These findings support a model in which the dominant phenotype depends on which partners and modifications are engaged, rather than implying a universal mechanism. Pharmacologic proof of concept comes from gastric cancer, where YBX1 inhibition increases ROS, disrupts DNA damage repair, promotes senescence, and modulates mTOR signaling, exposing stress vulnerabilities suitable for “one-two punch” senolytics strategies (171). Resistance is the clinical phenotype of the adaptive RNA hub.

### 8.2 Cytotoxic stress tolerance: chemo-, radio-, platinum-, and DNA-damage resistance

The available evidence supports a model in which YBX1 helps tumor cells survive acute therapeutic stress not through a single dominant mechanism but through partner- and context-dependent circuits that converge on four resistance logics: stress-transcript adaptation, DNA-damage and ROS buffering, ubiquitin–proteasome balance, and apoptosis pathway suppression.

A first logic operates through ncRNA-guided recruitment of YBX1 to stress-response transcripts. In cervical adenocarcinoma, CYP4A22-AS1 binds YBX1 and promotes its nuclear translocation, activating PGK1 transcription and a glycolytic adaptation that appears to reduce radiosensitivity (172). In non-small-cell lung cancer (NSCLC), IGFL2-AS1 recruits YBX1 to the HSPA1A promoter to engage a stress-response cascade through RAP1, contributing to cisplatin and 5-fluorouracil resistance that can be attenuated by pharmacologic HSPA1A inhibition (173). A circRNA–miRNA–YBX1 axis (circRAD23B/miR-1287-5p/YBX1) similarly underlies carboplatin resistance in ovarian cancer cell lines and patient-derived organoids (174). In intrahepatic cholangiocarcinoma, YBX1 promotes stem-like states and cisplatin insensitivity through an AKT/β-catenin axis, which should be interpreted as a tumor-specific stemness mechanism rather than a universal cytotoxic-tolerance pathway (175).

A second logic engages DNA damage and ROS buffering. In gastric cancer, TAGLN2 activates AKT–YBX1 signaling, enhances YBX1 phosphorylation and nuclear translocation, and promotes ssDNA aggregation with cGAS-STING/ISG-signature induction that supports chemo- and radioresistance (147). In osteosarcoma, NSUN6-mediated m5C marks on PEX1/PEX3 mRNAs are read by YBX1 to maintain peroxisome biogenesis and catalase output, buffering ROS and sustaining cisplatin resistance, with MARCH8-mediated NSUN6 ubiquitination dismantling the loop (123).

A third logic operates through ubiquitin–proteasome regulation of YBX1 or its restraining partners. SIAH1-mediated K304 ubiquitination of YBX1 destabilizes its m5C-target mRNAs and restores cisplatin sensitivity in epithelial ovarian cancer (176). Conversely, gemcitabine/cisplatin-induced NF-κB activation of OIP5 recruits TRIP12 to degrade PPP1CB and amplify YBX1 transcriptional activity in bladder cancer, forming a therapy-induced feedback loop (177). In breast cancer, TRIM25 degrades BRD7 via K48-linked ubiquitination and activates the YB1/Bcl-2 axis to support paclitaxel resistance through apoptosis suppression (178).

A fourth logic centers on apoptosis buffering and counter-regulatory restoration. MDM1 appears to restrain YBX1 binding to the TP53 promoter, supporting p53 expression and apoptosis to enhance chemoradiotherapy sensitivity in colorectal cancer (179). Restoring miRNA repression of YBX1, by miR-137 in colon cancer (oxaliplatin) (180) or by demethylation-restored miR-375 in breast cancer (multidrug resistance) (181), sensitizes resistant cells, and depletion of YBX1 partners NONO and RALY reverses YBX1-induced oxaliplatin resistance in colorectal lines (182). Pharmacologic proof of concept comes from the small-molecule YBX1 inhibitor SU056, which suppresses mRNA-processing programs, induces G1 arrest, restricts drug efflux, and synergizes with cisplatin or paclitaxel in high-grade serous ovarian carcinoma models with limited toxicity (42, 183). Together, these findings support YBX1 as a tunable adaptive node rather than implying a universal driver of cytotoxic stress tolerance across tumor types.

### 8.3 Autophagy, PANoptosis suppression, ferroptosis avoidance, and survival buffering

The available evidence supports a model in which YBX1 helps tumor cells endure therapy-induced metabolic, oxidative, and inflammatory stress by buffering several regulated cell-death pathways rather than by suppressing a single program. Autophagy-mediated tolerance is the most consistently reported buffering mechanism, though its molecular routing is tumor-specific. In NSCLC, YBX1 may engage the p110β/Vps34/beclin1 axis to sustain autophagy and reduce cisplatin sensitivity (184). In gastric cancer, YBX1 acts as an m5C reader that stabilizes ATG9A mRNA through NSUN2-mediated modification, enhancing autophagic flux and contributing to 5-fluorouracil resistance (27). A complementary layer operates on YBX1 itself: in hepatocellular carcinoma, circZNF79(5) recruits the K63 deubiquitinase BRCC36 to shield YBX1 from p62-mediated selective autophagic degradation through the AMPK/mTOR axis, illustrating how circRNA-controlled YBX1 stability feeds back on the autophagic machinery (61).

A distinct branch involves suppression of inflammatory-regulated cell death. In gastric cancer, YBX1 appears to inhibit PANoptosis, with its activity tuned by PPM1B-mediated dephosphorylation at S314 and USP10-mediated deubiquitination, contributing to oxaliplatin chemoresistance (50). This evidence should be interpreted cautiously, as PANoptosis findings remain context-specific. Ferroptosis is the third arm of this buffering architecture, alongside autophagy and PANoptosis suppression; its amino-acid (ODC1/SLC7A11) and cholesterol (SREBP2/ABCA1) mechanisms are fundamentally metabolic and are developed in Section 7.3 (136, 142).

### 8.4 Adaptive bypass in targeted and endocrine therapy resistance

Where a targeted- or endocrine-resistance phenotype rests on a metabolic mechanism already developed in Section 7.3 (SREBP2/cholesterol-driven sorafenib tolerance (136)), it is not restated here; the emphasis in this subsection is on the contribution of such circuits to therapeutic escape. The available evidence supports a model in which YBX1 functions as an adaptive bypass builder in targeted and endocrine therapy resistance, allowing tumor cells to maintain signaling continuity once the primary therapeutic dependency is blocked, rather than acting as a generic resistance marker.

Phosphorylation-dependent bypass is a recurring theme. In HCC, RIOK1 interacts with YBX1 to promote Ser165 phosphorylation and nuclear localization, activating JAK2/STAT3 signaling and elevating lenvatinib resistance (49). A parallel stress-activated route involves STK24-driven MASTL activation, which phosphorylates YBX1 and licenses transcriptional induction of PAK4, with disruption of the MASTL/YBX1/PAK4 axis restoring lenvatinib sensitivity (131). In antiestrogen-resistant breast cancer, AKT/p70S6K/p90RSK-mediated Ser102 phosphorylation supports nuclear YBX1 activity, and the multi-kinase inhibitor TAS0612 combined with everolimus suppresses this phosphorylation and overcomes resistance in preclinical models (46).

A second logic operates through RNA-level stabilization and m5C-mediated translation. In HCC, circRNA-SORE binds cytoplasmic YBX1, prevents PRP19-mediated ubiquitin-dependent degradation, and is exosomally transmitted to spread sorafenib resistance (117), consistent with broader implication of YBX1 in HCC sorafenib resistance whose mechanistic resolution remains partial (25). In EGFR-mutant non-small-cell lung cancer, NSUN2-deposited m5C marks on QSOX1 are read by YBX1 to enhance translation, driving intrinsic gefitinib resistance (66).

A third logic involves RTK/β-catenin and PI3K/AKT/mTOR rewiring. In pancreatic ductal adenocarcinoma, YBX1 transactivates LRP1 to redistribute β-catenin, which through TCF3 induces RRM1 and gemcitabine resistance; combining the YBX1 inhibitor SU056 with gemcitabine reverses this phenotype in preclinical models (185). In breast cancer, YBX1 accelerates proteasomal ER degradation, transactivates ERBB2, and contributes to fulvestrant and tamoxifen resistance (186), while YBX1 and NFIB jointly bind the ESR1/FOXA1 complex and FGFR2 signaling augments this interaction to repress estrogen responsiveness (187). A YBX1–lncRNA SBF2-AS1 positive feedback loop further sustains PI3K/AKT/mTOR signaling and tamoxifen resistance (75).

A fourth logic couples endocrine resistance to immune escape. In enzalutamide-resistant prostate cancer, FLII downregulation releases YBX1 to translocate into the nucleus and transactivate PD-L1, supporting enzalutamide resistance, suppressing CD8+ effector responses, and increasing Treg and MDSC infiltration; restoring FLII reverses both resistance and immune evasion (43). These observations should be interpreted cautiously as preclinical proof of concept rather than implying a universal bypass mechanism across tumor contexts.

Across the resistance logics described above, an emerging set of interventions indicates that this architecture is pharmacologically tractable rather than merely descriptive: the same circuits can in principle be cut at YBX1 itself, at its activating modifications and localization, at its RNA-modification context, or at the downstream metabolic and survival nodes it controls. Because these agents sit at the interface between resistance biology and therapeutic strategy, they are examined together in Section 10, direct inhibition (10.2), state disruption (10.3), and circuit breaking (10.4), rather than enumerated here. What matters for the present argument is the principle: if resistance is the clinical phenotype of an adaptive RNA hub, then every layer that sets the hub’s state, not only the hub itself, is a candidate point of intervention.

## 9 Family-level Y-box dependencies: tumor-specific allocation of YBX1, YBX2, and YBX3 circuits

### 9.1 Why YBX1 alone is not enough: the need for a Y-box dependency profile

The available evidence supports a model in which the biological relevance of YBX1 cannot be captured by total protein abundance alone, since each tumor appears to operate through a specific configuration of Y-box family members, post-translational modifications, subcellular localization, RNA-binding partners, and m5C-reader context. A comprehensive pan-cancer analysis across 28 cancer types found that YBX1, YBX2, and YBX3 collectively associate with prognosis, malignant pathways, tumor microenvironment features, treatment response, and drug resistance, and that a composite YBXs score derived from all three members predicts therapeutic efficacy, particularly for immunotherapy—more informatively than any single member considered alone (99). In this dataset, YBX2 emerged as a tissue-specific candidate biomarker in liver cancer, although this should be interpreted cautiously rather than as a universally predictive marker. Consistent with this family-level logic, an independent pan-cancer analysis identified the lncRNA-HEIH/YBX3 axis as an immune-oncogenic system in which YBX3 tracks with immune-cell infiltration and T-cell exclusion across tumor types and, in immune-checkpoint-blockade sub-cohorts, predicted response and survival more accurately than several standardized biomarkers, with the strongest signal in colon cancer (188). Together with the YBX2-weighted composite score (99), this reinforces that the predictive unit is the configured family rather than any single member, though both rest on retrospective, database-derived cohorts and require prospective validation.

Mechanistic work in NSCLC reinforces the same point at the circuit level. NSUN2 deposits m5C marks on YAP mRNA, and ALYREF, together with YBX1, reads these marks to stabilize and translate YAP, supporting growth, EMT, invasion, and metastasis (63). In this context, the functional output of YBX1 depends on its writer and adaptor partners and on the m5C-modified transcriptome it engages, rather than on its expression level alone. Therapy should not be designed against total YBX1 expression alone; it should be designed against the dominant Y-box family circuit active in that tumor.

### 9.2 Alternative and cooperative Y-box circuits: YBX3-dominant programs and YBX1/YBX3 immunotherapy co-dependency

The available evidence supports a model in which Y-box family biology is not reducible to YBX1, and tumor-specific dependency may be carried by YBX3-dominant circuits or by cooperative YBX1/YBX3 hubs, particularly in immune evasion, rather than implying functional equivalence between family members.

Several tumors appear to operate through YBX3-dominant m5C-reading or stability circuits. In Epstein-Barr virus-associated nasopharyngeal carcinoma, LMP1-driven NF-κB activation upregulates NSUN2, which deposits m5C on ICAM-1 mRNA; YBX3 recognizes the modified site and recruits PABPC1 to enhance ICAM-1 translation, supporting metastasis (64). In clear cell renal cell carcinoma, USP18 deubiquitinates and stabilizes YBX3, sustaining PI3K/AKT signaling and tumor progression (106). These contexts illustrate that the m5C-reader and pro-oncogenic outputs in some tumors are carried predominantly by YBX3 rather than YBX1.

The role of YBX3 is not uniformly oncogenic, however, and should be interpreted cautiously. In colorectal cancer, YBX3 normally promotes SLC7A11 degradation and ferroptosis susceptibility; USP5 redirects YBX3 to lysosomal degradation, which stabilizes SLC7A11 and supports ferroptosis-resistant survival (143). In hepatocellular carcinoma, TTC36 stabilizes YBX3 by masking K311/K350 ubiquitination sites, and stabilized YBX3 enhances SPRED1 mRNA to suppress Ras/MAPK-driven proliferation; this tumor-suppressive axis paradoxically induces sorafenib resistance through compensatory PI3K/Akt activation that is reversible by combined Akt inhibition (189). These paradoxical roles indicate that family-level profiling should consider how a specific Y-box protein is stabilized, which RNA modification it reads, and which downstream survival circuit it engages.

**Figure 7:**
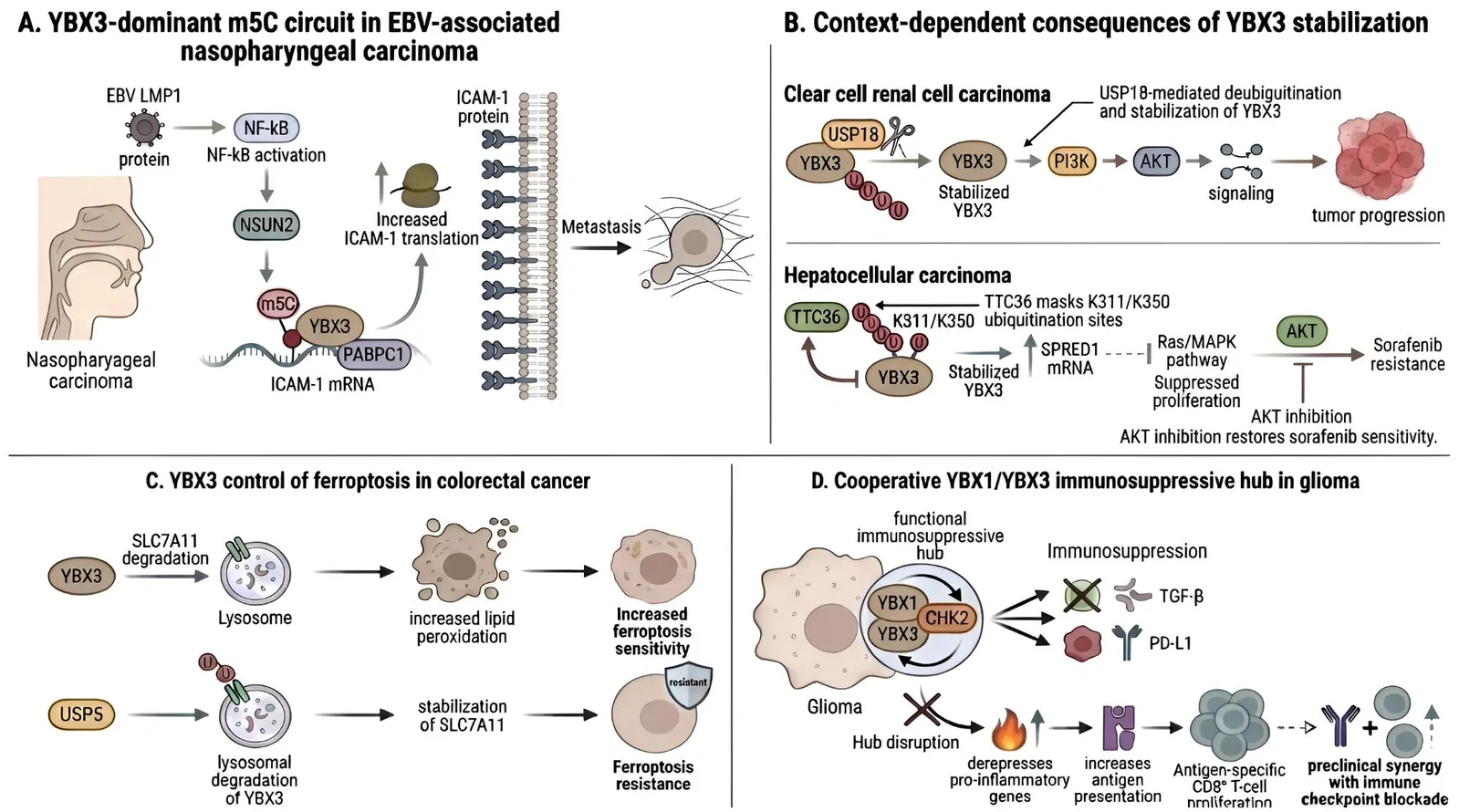
Alternative and cooperative Y-box-dependent oncogenic circuits: YBX3-dominant programs and YBX1/YBX3 co-dependency. **(A)** In EBV-associated nasopharyngeal carcinoma, the LMP1–NF-κB–NSUN2 axis promotes m5C-dependent YBX3/PABPC1-mediated ICAM-1 translation and metastasis. **(B)** YBX3 stabilization has context-dependent effects, supporting PI3K–AKT-driven progression in clear cell renal cell carcinoma while suppressing Ras/MAPK proliferation yet enabling compensatory AKT-dependent sorafenib resistance in hepatocellular carcinoma. **(C)** In colorectal cancer, YBX3 promotes SLC7A11 degradation and ferroptosis sensitivity, whereas USP5-mediated lysosomal degradation of YBX3 stabilizes SLC7A11 and confers ferroptosis resistance. **(D)** In glioma, a cooperative CHK2–YBX1–YBX3 hub sustains immunosuppression, while its disruption enhances inflammatory gene expression, antigen presentation, antigen-specific CD8+ T-cell proliferation, and responsiveness to immune checkpoint blockade.

In glioma, YBX1 and YBX3 act cooperatively rather than alternatively. CHK2, YBX1, and YBX3 form a reciprocally reinforcing immunosuppressive hub whose disruption derepresses pro-inflammatory gene expression, enhances antigen presentation and antigen-specific CD8+ T-cell proliferation, and synergizes with immune checkpoint blockade in preclinical models (148). This co-dependency provides a clear example of why family-level targeting may outperform single-member strategies in immunologically silenced tumors (**Figure 7**).

## 10 Therapeutic strategy and testable predictions

### 10.1 Patient stratification: first identify the active YBX circuit

The available evidence suggests that selecting a YBX-directed strategy should begin with stratification rather than inhibition, integrating several layers of information that no single readout captures alone. In hepatocellular carcinoma, YBX1 expression appears prognostic and correlates with immune infiltration and lipid-metabolism programs (95), and bulk and single-cell analyses link high YBX1 in liver hepatocellular carcinoma to T-cell dysfunction, exclusion phenotypes, and predicted poor immunotherapy response (100), yet expression alone may be insufficient for therapy selection.

A first stratification layer is family-level profiling. A pan-cancer YBXs score combining YBX1, YBX2, and YBX3 may help stratify malignant pathway activity, tumor microenvironment phenotype, and treatment response, including immunotherapy efficacy, more informatively than YBX1 alone, with YBX2 emerging as a tissue-restricted candidate biomarker in liver cancer that requires prospective validation (99).

A second layer defines the RNA-modification context. Pan-cancer analysis of m5C readers indicates that ALYREF and YBX1 status, together with their associations with eIF-related programs, immune infiltration, and drug-sensitivity patterns, should be interpreted jointly rather than separately (94). In pancreatic ductal adenocarcinoma, an m5C-regulator signature score correlates with the squamous/basal subtype, Ras/MAPK/PI3K activation, immune exclusion, low CD8+ infiltration, and higher PD-L1 expression, and may also forecast sensitivity to PARP, EGFR, AKT, HER2, or mTOR inhibitors (110).

A third layer is spatial and cell-type resolution. Single-cell and spatial analyses in glioblastoma show that YBX1 activity differs between neoplastic compartments and distinct myeloid populations, with cell-type-specific NF-κB and TNF programs reshaping the immunosuppressive microenvironment, supporting compartment-resolved stratification rather than bulk expression (98).

Integrating these layers- Y-box family state, m5C-reader and modification context, immune ecology, and dominant downstream circuit—could inform which therapeutic vulnerability is most actionable in each tumor, although prospective validation remains essential. The therapeutic problem is not whether YBX1 is high; it is which YBX-dependent circuit is active and where that circuit can be cut with the least adaptive escape.

### 10.2 Direct YBX1 inhibition: broad but potentially powerful

The available evidence supports a proof-of-concept model in which direct YBX1 inhibition could collapse multiple resistance circuits at once, since a single hub feeds RNA-processing, signaling, metabolic, and survival outputs. This breadth is both the central rationale and the central caveat, as broad activity raises specificity, delivery, and toxicity questions that current preclinical evidence does not fully resolve.

The most advanced direct YBX1 inhibitor candidate is SU056. In high-grade serous ovarian carcinoma patient-derived xenografts and syngeneic models, SU056 suppresses YBX1 and its mRNA-processing programs, delays tumor progression, and enhances cisplatin- and paclitaxel-mediated antitumor activity with extended survival (183). A complementary multi-target strategy is illustrated by FKA-9i, a Flavokawain A sulfonamide derivative that directly binds LRPPRC, YBX1, and RPN1 to destabilize their interactions and disrupt MAPK-linked tumor progression, inducing cell-cycle arrest, mitochondrial dysfunction, ROS accumulation, and apoptosis with synergy when combined with chemotherapy or glycolysis-directed agents; this should be interpreted as a multi-target chemical scaffold rather than a YBX1-selective probe (190). Early-stage drug repurposing in hepatocellular carcinoma identified candidate compounds predicted to interact with the cold-shock domain of YBX1, with glycine experimentally selected as a low-toxicity YBX1-suppressing molecule that supports feasibility but not clinical readiness (191). Functional inhibition studies in gastric cancer further indicate that suppressing YBX1 increases ROS, disrupts DNA-damage repair, modulates mTOR signaling, and exposes senescence-associated vulnerabilities suitable for “one-two punch” combinations (171).

Direct YBX1 inhibition appears most rational when tumors depend on nuclear or phosphorylated YBX1, RNA-processing programs, or multiple convergent YBX1 outputs, but specificity, delivery, compensatory-circuit risks, and patient-selection criteria require further validation. A further on-target risk is immunological: because YBX1 has documented roles in normal immune cells, enhancing immunoglobulin mRNA translation in B cells (149), and operating within the PSAT1–YBX1–HLA-E axis relevant to NK-cell biology (40), systemic inhibition, particularly in combination with checkpoint blockade, could impair normal humoral and innate immune function, so tumor-restricted delivery and preclinical immunotoxicity assessment in immunocompetent models would be prerequisites for translation.

A specific and underexamined dimension of this specificity problem is intra-family selectivity: because the cold-shock domain is highly conserved across YBX1, YBX2, and YBX3, it is not established that SU056, or other CSD-directed agents, discriminate YBX1 from its paralogs, and a pan-CSD inhibitor could disable tumor-suppressive family members such as YBX3 (for example, the TTC36–YBX3–SPRED1 axis (189)). Direct inhibition should therefore be operationalized within the family-level stratification logic of Section 9 and considered only where YBX1 is the dominant oncogenic family member and YBX2/YBX3 are not tumor-suppressive in that context.

### 10.3 State disruption: target the active YBX1 pool, not total YBX1

The available evidence supports a model in which the actionable unit of YBX1 may be its active state, defined by phosphorylation, nuclear localization, and partner engagement, rather than total protein abundance. Total YBX1 may be insufficient as a standalone biomarker, while state-resolved readouts could guide both patient selection and the choice of pathway to disrupt.

Phosphorylation at Ser102 by AKT, p70S6K, and p90RSK promotes YBX1 nuclear translocation and resistance-gene expression, and elevated phospho-YBX1 (pYBX1) accompanies acquired resistance to fulvestrant and tamoxifen; the multi-kinase inhibitor TAS0612 and the mTORC1 inhibitor everolimus suppress this phosphorylation and overcome antiestrogen resistance in preclinical models, with immunohistochemistry identifying pYBX1 enrichment in patients recurring during adjuvant endocrine therapy and in triple-negative breast cancer (46). A complementary strategy is upstream pathway suppression: in colorectal cancer, the PI3K inhibitor BKM120 partially reverses YBX1-driven proliferation and migration, consistent with a YBX1–PI3K/AKT regulatory axis amenable to state-level disruption rather than direct protein elimination (192). State-directed targeting must, however, contend with compensatory signaling. In KRAS-mutated colorectal cancer, RSK-mediated YBX1 phosphorylation supports cetuximab resistance, and inhibition of RSK activates compensatory Akt; dual RSK and Akt targeting suppresses proliferation and sensitizes cells to 5-fluorouracil, with phosphorylated and nuclear YBX1 identifying the resistant signaling state (137).

Beyond phosphorylation, partner-dependent shuttling may also be tractable. In hepatocellular carcinoma, celecoxib turns off the Hippo pathway, initiates YAP-driven nucleocytoplasmic shuttling of the YAP–YBX1 complex, and suppresses PTGS2/COX-2 transcription to trigger mitochondrial apoptosis (193). Together, these findings suggest that state-resolved YBX1 biomarkers and combined kinase- or localization-disrupting agents may be more informative than total YBX1, though prospective validation remains required.

### 10.4 Circuit breaking: collapse the dominant dependency

The available evidence supports a circuit-breaking model in which YBX1-directed therapy may be most effective when combinations are matched to the dominant dependency sustaining a given tumor state, rather than to total YBX1 abundance. The actionable target can be YBX1 itself, its activating state, its RNA-modification context, or a downstream node, and the choice should follow the active circuit identified by stratification.

Immune-silent ecologies call for co-targeting the YBX1–PD-L1/m5C–immune axis together with checkpoint blockade. The mechanistic basis for these axes is developed in Sections 6.2 and 6.6; the prediction that follows is specific. In tumors whose immune exclusion is YBX1-dependent, disrupting the operative node, the NSUN5/circPPAP2A/YBX1 circuit in clear cell renal cell carcinoma (122), CDKL1-mediated displacement of YBX1 from the PD-L1 promoter in lung cancer (135), or YBX1 knockdown under chemotherapy in hepatocellular carcinoma (78), should restore effector CD8+ activity and synergize with anti-PD-1/PD-L1, with contraction of Tregs, MDSCs, and M2 macrophages as the expected pharmacodynamic readout.

Metabolic dependencies provide a distinct vulnerability. In oxaliplatin-resistant gastric cancer, bufalin directly binds YBX1 and collapses the STC1/PI3K–Akt/HK2 glycolytic axis to restore chemosensitivity (156). A complementary ferroptosis-induction route operates through the ODC1/YBX1/SLC7A11 dependency in gastric adenocarcinoma, which can be broken by erastin combined with 5-fluorouracil to re-establish chemosensitivity (142).

Adaptive-bypass signaling represents a fourth circuit. In pancreatic ductal adenocarcinoma, combining the YBX1 inhibitor SU056 with gemcitabine reduces resistance driven by the LRP1/β-catenin/RRM1 axis (185). In hepatocellular carcinoma, dual pharmacologic inhibition of YBX1 or SREBP2 with SU056/Betulin destabilizes membrane RTKs, suppresses PI3K/Akt/mTORC1 and EMT signaling, and restores sorafenib sensitivity (136) (its EMT and plasticity consequences are developed in Section 8.1). These strategies should be interpreted cautiously as preclinical proof of concept rather than as clinically established regimens, but together they support combination therapy guided by circuit dominance, immune, metabolic, ferroptotic, or bypass, as a testable translational framework that requires prospective validation across tumor contexts.

### 10.5 Testable predictions and experimental roadmap

This framework generates two classes of tests. The first (predictions 1–3) are circuit-specific replications: they re-examine, in independent cohorts and with prospective design, whether disrupting a defined circuit reproduces the immune or resistance shifts reported in its founding study (78, 122, 148); failure to reproduce would weaken the corresponding circuit claim. The second class is the framework-discriminating test, which no single primary study supplies: across tumor types, state-resolved YBX1 readouts (phospho-S102, nuclear-to-cytoplasmic ratio, ALYREF/YBX1/YBX3 m5C-reader context, dominant ncRNA scaffold) should predict downstream circuit activity and therapy response more accurately than total YBX1 abundance. If they do not, the hub model is falsified in favor of YBX1 as a promiscuous, abundant RNA-binding protein. The remaining predictions elaborate the first class.

First, in gliomas displaying a CHK2–YBX1/YBX3 hub, disruption of any hub member should increase pro-inflammatory gene expression, antigen presentation, and antigen-specific CD8+ T-cell proliferation, and should improve immune checkpoint blockade (ICB) response relative to ICB alone (148); this prediction would be weakened if hub disruption fails to alter antigen presentation or extend survival.

Second, in clear cell renal cell carcinoma with high NSUN5/circPPAP2A/YBX1 activity, disrupting this m5C-modified circRNA–reader circuit should restore CCL5, reduce TGFB1, increase CD8+ infiltration, and synergize with anti-PD-1 therapy (122); failure to observe these immune shifts after axis disruption would weaken the prediction.

Third, in chemoresistant tumors driving PD-L1 expression through YBX1, combined cytotoxic and immune-restorative therapy should outperform either modality alone, with reductions in MDSCs and regulatory T cells and expansion of cytotoxic CD8+ T cells serving as the expected readouts (78).

Fourth, ALYREF/YBX1 m5C-reader profiles combined with TIDE scores, eIF-linked translation states, and immune-infiltration patterns may stratify response to drug and immunotherapy interventions; this is testable by showing distinct response distributions across reader-defined subgroups in retrospective cohorts and prospective trials (94).

Fifth, compartment-resolved analyses in glioblastoma predict that neoplastic and myeloid YBX1 states generate divergent NF-κB and TNF outputs; bulk-tissue assays should therefore underperform single-cell or spatial readouts in predicting immune phenotype and ICB response (98). These predictions remain preclinical and require prospective validation, but each is falsifiable and experimentally actionable, providing a roadmap that converts the YBX-circuit framework from concept into testable translational science.

### 10.6 Challenges: the gap between a tractable concept and a validated therapy

The strategies above describe where a YBX1-directed intervention could in principle be applied, not a body of validated therapeutics. Several obstacles separate the two, and because the hub framework is only useful if it is honest about its own translational distance, we state them explicitly rather than leaving them implicit in the evidence tiers of the preceding tables. The first is the difficulty of drugging an RNA-binding protein at all. YBX1 has no enzymatic active site and no deep hydrophobic pocket of the kind that small molecules exploit in kinases; its functionally critical surfaces are the cold-shock domain’s RNA-binding face and the intrinsically disordered C-terminal region that mediates phase separation, neither of which presents a conventional druggable cleft. This is a class problem for RNA-binding proteins, not a YBX1-specific one, and it explains why the agents discussed here act indirectly: SU056 through an allosteric or aggregation mechanism (183), FKA-9i by disrupting a protein–protein interaction rather than occupying an active site (190), and the repurposed glycine candidate through a low-affinity cold-shock-domain interaction that supports feasibility rather than potency (191). None is a validated, selective, high-affinity YBX1 inhibitor, and no YBX1-directed agent has entered clinical trials.

The second is selectivity, at two levels. Intra-family selectivity is unresolved: the cold-shock domain is highly conserved across YBX1, YBX2, and YBX3, so a cold-shock-domain-directed agent risks disabling tumor-suppressive family members, the TTC36–YBX3–SPRED1 axis being the clearest example (189), and it is not established that any current compound discriminates YBX1 from its paralogs. Inter-protein selectivity is equally uncertain, since the cold-shock fold is shared with other nucleic-acid-binding proteins. This is the molecular basis of the redundancy problem: the Y-box family and the wider set of m5C readers overlap functionally, so inhibiting YBX1 alone may be buffered by ALYREF or YBX3 in tumors where reading is distributed across several proteins, exactly the cooperative configurations documented in Sections 5.2 and 9.

The third is toxicity, and it is specifically an on-target immunological risk rather than a generic one. YBX1 has documented functions in normal immune cells, translational control of immunoglobulin transcripts in B cells (149) and participation in the PSAT1–YBX1–HLA-E axis relevant to NK biology (40), so systemic inhibition, particularly if combined with checkpoint blockade as several circuit-breaking strategies propose, could impair humoral and innate immunity in the very setting where it is meant to help. Tumor-restricted delivery and immunotoxicity assessment in immunocompetent rather than xenograft models are therefore prerequisites, not refinements.

The fourth is that context-dependence, the central claim of this review, is itself a therapeutic liability. A protein whose output depends on its state cannot be targeted by a strategy that ignores state: the same intervention that collapses an oncogenic circuit in one tumor may be inert, or may release a tumor-suppressive YBX3 function, in another. This is why the counter-regulatory circuits cataloged throughout Layer 3- CXCL2-restrained nuclear entry (133), the ST7 (134) and circRABL2B (170) tumor-suppressive axes, MDM1-restrained TP53 (179) binding- are not footnotes but design constraints: they mark the tumors in which YBX1 inhibition would be actively harmful.

The fifth is that the biomarkers required to navigate all of the above do not yet exist as validated assays. The stratification logic of Section 10.1 and the state-resolved readouts of Section 10.3, phospho-S102, nuclear-to-cytoplasmic ratio, m5C-reader context, and dominant scaffold, are analytically reasonable but none has been prospectively validated as predictive of response to a YBX1-directed agent, for the simple reason that no such agent has been tested prospectively. Biomarker development and therapeutic development are therefore mutually blocking, and neither can advance far without the other.

To keep these caveats from being lost in the narrative, **Table 7** separates the therapeutic concepts discussed in this review into those supported by mechanistic evidence (M), those supported primarily by associative evidence (A), and those that remain hypothesis-generating therapeutic inferences (P). The distinction is not between good and bad ideas but between what has been shown and what has been proposed, and the honest position of the field is that almost everything in the second column is currently larger than the first.

## 11 Emerging technologies and future directions

The framework advanced here reframes YBX1 biology as five unresolved questions—what YBX1 binds, how binding shifts between states, which cell populations depend on it, which circuits are active in a given tumor, and which patients will benefit from YBX1-directed therapy. Emerging technologies are most usefully judged by which of these they can resolve.

### 11.1 What RNA does YBX1 bind, and how does binding change between states?

The interactome is already well mapped: iCLIP in glioblastoma defined a UYAUC consensus and pre-miRNA loop binding (194), Fusion-CLIP/PAR-CLIP expanded the CAUC motif to UCUUUNNCAUC and recovered hundreds of novel targets (195), and PAR-CLIP in medulloblastoma linked occupancy to CBX5-dependent apoptosis programs (196). SELEX and crystallography resolved CA(U/C)C recognition through conserved π-stacking and a Tyr99/Asp105 dimer interface (19, 197), while m5C recognition was localized to the W65 indole ring, coupled to ELAVL1 recruitment (198). Recent studies extend this paradigm to m5C-dependent stabilization of transcripts including RNF115, ATF4 and ATG9A (27, 31, 199). This binding is in turn redirected by lncRNA and circRNA scaffolds that reposition YBX1 onto specific promoters and transcripts (74, 200, 201) and by small RNAs that bind or displace it (118, 202). Similarly, lncRNA-mediated recruitment provides an additional layer of context-dependent YBX1 regulation, exemplified by lincNMR/YBX1/RRM2 and related scaffold-dependent circuits that redirect YBX1 activity toward specific cancer programs (201). Crucially, S102 phosphorylation destabilizes the cold-shock domain and reduces binding (203), yet no interactome has been resolved by phosphorylation or localization state, and family redundancy, YBX3 substituting for YBX1 in global binding (204), remains unmodelled; state-resolved CLIP is the direct future test of the state code.

### 11.2 Which cell populations depend on YBX1?

Single-cell multiomics has begun to answer this: a scRNA-seq pipeline across 35,000 prostate cells nominated YBX1 as the malignant proliferative hub within an immune-excluded microenvironment marked by dendritic-cell and CD8+ depletion (205), and CRISPRi-coupled multiomics defined a DARS1-AS1/YBX1 circuit in glioblastoma stem-like cells (74). Beyond malignant-cell intrinsic programs, YBX1 also contributes to tumor–immune communication, with YBX1-dependent macrophage polarization and CXCL8 mRNA stabilization linking YBX1 activity to immune and metastatic niches (206). Because bulk assays obscure these compartment-specific states, as this Review emphasizes, extending them with spatial transcriptomics is a clear priority, though no dedicated spatial YBX1 study yet exists, making this an opportunity rather than an established capability.

### 11.3 Which circuits are active in a given tumor?

YBX1-centred network analysis has nominated context-specific partners such as PABPC1 in lung adenocarcinoma (207), providing the biological foundation on which AI could integrate interactome, m5C-landscape, single-cell and protein–RNA datasets. Although AI approaches have not yet predicted YBX1-specific regulatory circuits, existing YBX1 interactome maps and network analyses provide the biological substrate for future models integrating RNA binding, epitranscriptomic state and cellular context (208, 209), but none yet predicts tumor-specific YBX1 circuits; this integration is a future opportunity, not a current tool.

### 11.4 Which patients benefit, and through what intervention

Therapeutically, three modalities have preclinical YBX1 support: amido-bridged antisense oligonucleotides (210), RNA-interference silencing with cisplatin resensitization (211), and an integrative screen yielding small molecules that disrupt YBX1–mRNA binding in cells (212); genetic dependency studies indicate that selected tumor contexts may exhibit vulnerability to YBX1 loss (28), providing a rationale for future YBX1-centred synthetic-lethality screens (213) and broader RNA-therapeutic platforms (214). Targeted protein degradation and molecular-glue approaches against YBX1, by contrast, are unreported and remain conceptual. For patient selection, YBX1 overexpression predicts prognosis across cancers (32, 95, 99), associates with immune-related tumor features and immunotherapy-associated signatures (94, 206), and is detectable in circulating exosomes as a liquid-biopsy candidate (215). These measure abundance; the transition this framework demands is toward state-resolved biomarkers incorporating phosphorylation (216), localization and scaffold engagement (74), and m5C context (94), none yet clinically validated. The future of YBX1 research therefore lies not in confirming that YBX1 is present, but in resolving which state it occupies, in which cell, controlling which program, and whether that state predicts therapeutic vulnerability.

## 12 Counter-evidence, contradictions, and unresolved controversies

A framework built on context-dependence is obliged to take seriously the evidence that does not fit a simple oncogenic reading. We gather that evidence here rather than leaving it distributed, because its weight bears directly on how confidently the hub model can be held.

### 12.1 YBX1 activity is not uniformly tumor-promoting

In several settings, the tumor-suppressive phenotype follows from restraining YBX1 rather than from activating it. Intracellular CXCL2 binds YBX1 and blocks its nuclear entry, reducing SREBF2-driven cholesterol biosynthesis and favoring antitumor neutrophil polarization (133); ST7 binds YBX1 and prevents nuclear translocation, suppressing MMP14 and restraining EMT and metastasis (134); MDM1 restrains YBX1 occupancy at the TP53 promoter, supporting p53-dependent apoptosis and chemoradiotherapy sensitivity (179); and circRABL2B–YBX1 inhibits MUC5AC, reducing stemness and enhancing erlotinib sensitivity (170). In each, the same node that drives malignancy elsewhere is the node whose restraint is protective. These are not anomalies to be explained away: they are the clearest available evidence that YBX1’s output is set by state rather than by abundance.

### 12.2 Non-coding RNA interactions with YBX1 run in both directions

The piRNA piR-YBX1 binds YBX1 mRNA and downregulates both transcript and protein, suppressing MAPK signaling and tumor growth in triple-negative breast cancer (118), directly opposing the stabilizing circRNAs described in the same layer. Within a single tumor type, the direction can invert: colorectal cancer contains both an ncRNA that recruits the E3 ligase Nedd4L to degrade YBX1 (102) and one that suppresses its polyubiquitination to stabilize it (103). Ubiquitin biology shows the same duality, with SIAH1-mediated ubiquitination destabilizing YBX1 targets and restoring cisplatin sensitivity (176), and restored miRNA repression re-sensitizing resistant cells (180).

### 12.3 Family members can act oppositely to YBX1, and to each other

YBX3 is not a redundant paralogue. In hepatocellular carcinoma, TTC36-stabilized YBX3 enhances SPRED1 to suppress Ras/MAPK-driven proliferation, a tumor-suppressive axis that nonetheless induces sorafenib resistance through compensatory PI3K/Akt activation (189), a result that resists classification as either oncogenic or suppressive. In colorectal cancer, YBX3 normally promotes SLC7A11 degradation and ferroptosis susceptibility, and redirection of YBX3 to lysosomal degradation supports survival (143). Any therapeutic strategy directed at the conserved cold-shock domain must contend with these opposing family-level functions.

### 12.4 The same chemokine or effector can be regulated in opposite directions

CCL5 is induced by YBX1 in a lung adenocarcinoma circuit, where high expression is associated with better survival and immunotherapy response (151), and suppressed in a renal circuit that reduces CD8+ infiltration (122). This is difficult to reconcile with a fixed transcriptional output and is, we argue, precisely what a state-dependent regulator should produce, but we note that it could equally be read as evidence that the reported associations are context-specific artifacts rather than a coherent program.

### 12.5 Three controversies remain genuinely unresolved

First, whether YBX1 is a bona fide m5C reader in the biochemical sense: the functional evidence is strong, but no structural or biophysical measurement demonstrates preferential binding to modified over unmodified RNA. Second, whether the pan-cancer correlative literature reflects YBX1-specific biology or the proliferative state it marks, a distinction that at least one large pan-cancer analysis suggests may not be fully separable from proliferative state (32). Third, whether the absence of YBX1 circuits in certain domains, glutamine metabolism, dendritic-cell biology, pseudouridine recognition, reflects genuine boundaries of YBX1 function or simply questions the field has not asked. We have marked each of these as open rather than resolving them by assertion.

Taken together, this counter-evidence does not overturn the hub framework, but it constrains it: YBX1 cannot be described as an oncogene whose inhibition is uniformly desirable, and the circuits cataloged above identify the contexts in which YBX1-directed intervention would be ineffective or actively harmful.

## 13 Limitations

The nature of its underlying evidence constrains the synthesis advanced here, and several field-level weaknesses recur across the studies cited. First, a substantial fraction of the pan-cancer and immune-association claims rests on bulk-transcriptomic correlation and database-derived inference rather than directional, functional proof (94, 95, 99, 100, 110); where single-cell or spatial resolution is available, it reveals that bulk measurements can obscure compartment- and cell-type-specific YBX1 states (98), implying that much of the correlational literature may misassign activity that is in fact restricted to particular neoplastic or myeloid populations. Second, the mechanistic core of the model, that a defined state change reroutes a defined transcript set toward a defined phenotype, is unevenly supported: studies with direct m5C mapping, RIP/MeRIP, stability assays, mutagenesis, and rescue (62, 64–68) are far stronger than signature-level (110, 155) or expression-correlation reports (109, 154), and several conclusions derive from single cell lines, most notably A549-based ferroptosis data that should not be generalized (161). Third, a structural and biophysical account of how post-translational modifications and phase separation actually alter YBX1’s RNA-binding specificity is largely absent; modifications such as K92 lactylation (51) or arginine methylation (56) are shown to change binding behavior without a mechanistic, residue-level explanation of altered selectivity. The same structural gap applies to the m5C-reading axis itself: although m5C is presented here as the most tractable transcript-selection rule, no crystal structure, ITC, or SPR experiment has established how the YBX1 cold-shock domain discriminates m5C from unmodified cytosine, so the mechanistic tractability claimed for m5C-based selection in Section 5.2 faces the same unresolved structural question as PTM-altered binding specificity. The immunological account assembled here is uneven in a specific way. Checkpoint regulation and myeloid recruitment are well represented, whereas antigen-processing machinery and dendritic-cell biology are essentially unexamined, and the interferon-proximal circuits rest on two studies that were not designed to test the feedback relationship we infer between them.

Fourth, part of the most recent and conceptually pivotal evidence exists only as conference abstracts not yet peer-reviewed (62, 97, 111, 136, 152, 172, 183), and should be weighted accordingly. Finally, clinical validation is essentially absent: the pharmacologic agents discussed remain preclinical proofs of concept (136, 156, 171, 183, 185), no YBX1-directed therapy has entered trials, and no validated assay yet reports the active YBX1 state rather than its abundance. These constraints do not undermine the hub framework, but they define the evidentiary distance still to be closed.

## 14 Conclusion

The value of treating YBX1 as an adaptive RNA hub lies not in cataloging its functions but in converting their apparent incoherence into a single inferential problem: predicting a tumor’s phenotype from the state YBX1 occupies and the inputs that set it. The framework’s central strength is also its principal liability: a model flexible enough to accommodate context is flexible enough to accommodate almost any observation. The membership conditions of Section 2 are intended to constrain exactly this. A finding that does not engage a state code, a conditional repertoire, dual modality and closed-loop feedback is not evidence for the hub; it is merely evidence about YBX1. The hub thesis is therefore worth holding only if it is falsifiable, and its falsification is concrete: if state-resolved measurements (phosphorylation, localization, partner and m5C-reader context) do not predict downstream circuit activity better than total abundance (the discriminating test specified in Section 10.5), the hub collapses into a merely promiscuous, abundant RNA-binding protein whose correlations are epiphenomenal. For this test to be decisive rather than perpetually deferrable, “state-resolved measurement” must be specified in advance. We propose that a complete Layer 1 characterization for a given tumor type comprises, at minimum, phospho-S102 status, the nuclear-to-cytoplasmic ratio, m5C-reader context (ALYREF/YBX1/YBX3 co-expression), and the identity of the dominant ncRNA scaffold. A prediction that fails against this pre-specified panel must count as evidence against the hub model and cannot be rescued by invoking an unmeasured tumor-specific input. Tumor-type specificity, although biologically real, must therefore be treated as a testable variable rather than a *post-hoc* explanatory resource; operationally, this requires pre-registering each state-to-circuit prediction before it is tested. Without such pre-specification, a falsified prediction is indistinguishable from an incompletely measured one, and the framework’s falsifiability is nominal rather than genuine. The evidence assembled here favors the hub reading but does not yet establish it, because the field’s mechanistic spine, direct proof that a defined state change reroutes a defined transcript set toward a defined phenotype, remains thin and rests partly on correlational, single-cell-line, and not-yet-peer-reviewed data.
